# The Dolichopodidae (Diptera) of Montserrat, West Indies

**DOI:** 10.3897/zookeys.966.55192

**Published:** 2020-09-09

**Authors:** Justin B. Runyon

**Affiliations:** 1 Rocky Mountain Research Station, USDA Forest Service, 1648 S. 7th Avenue, Bozeman, Montana 59717, USA USDA Forest Service Bozeman United States of America; 2 Montana Entomology Collection, Montana State University, Room 50 Marsh Laboratory, Bozeman, Montana 59717, USA Montana State University Bozeman United States of America

**Keywords:** Biodiversity, Caribbean, checklist, inventory, Lesser Antilles, new species, West Indies

## Abstract

The long-legged flies (Dolichopodidae) of the island of Montserrat in the Lesser Antilles have been surveyed and include 63 species in 27 genera. The following eleven new species are described and illustrated: *Amblypsilopusmarskeae***sp. nov.**, *Medeteraiviei***sp. nov.**, *Medeteramontserratensis***sp. nov.**, *Systenusladonnae***sp. nov.**, *Thrypticusmediofuscus***sp. nov.**, *Chrysotusantillensis***sp. nov.**, *Chrysotuscallichromoides***sp. nov.**, *Chrysotusinterfrons***sp. nov.**, *Chrysotusmontserratensis***sp. nov.**, *Diaphorusrobinsoni***sp. nov.**, and *Sympycnusmontserratensis***sp. nov.** Six species have only been found on Montserrat (ca. 10% endemicity). Keys are provided to the genera and species on Montserrat, their known distribution summarized, and additional new island records provided for many species. *Asyndetuswirthi* Robinson is synonymized with *A.interruptus* (Loew) and *Achradoceraapicalis* (Aldrich) is removed from synonymy with *A.barbata* (Loew). *Diaphorusflavipes* Aldrich is transferred to *Chrysotus* as a new combination. A new replacement name, *Chrysotusmilvadu***nom. nov.**, is provided for the Nearctic *Chrysotusparvulus* Van Duzee. Lectotypes are designated for *Achradoceraapicalis* (Aldrich) and *Asyndetusfratellus* Aldrich. The fauna of Montserrat is summarized and compared with that of Dominica. Collecting methods are compared and threats to the dolichopodid fauna of Montserrat discussed.

## Introduction

Dolichopodidae, or long-legged flies, are one of the largest dipteran families with more than 7,500 described species worldwide (Bickel 2009). Adults are usually less than 6 mm in size and metallic green-blue to bronze in color, but fewer species are nonmetallic yellow to brown or black. Most dolichopodids can be easily recognized by their general habitus, slender build, long legs, metallic coloration, reduced wing venation, and hair-like arista. Long-legged flies can be found in all terrestrial habitats, but adults are most abundant in moist habitats and can be commonly found on coastal rocks, sand, moist ground, foliage, tree trunks, and rocks in or near running water. Larvae occur in mud, moist soil, leaf litter, moss, algal mats, decaying seaweed, under bark (usually associated with bark beetle galleries), in tree holes, and within plant tissues (Dyte 1959; Bickel 2009). Adults and larvae of most dolichopodid species are predators that feed on other small invertebrates (Ulrich 2004) and may play important roles controlling mosquitoes, bark beetles, and agricultural pests (e.g., Laing and Welch 1963; Nicolai 1995; Kautz and Gardiner 2019). An exception to this predatory lifestyle is *Thrypticus*, whose larvae are phytophagous and feed within stems of plants (Bickel and Hernandez 2004). Because of species’ high habitat specificity and putative sensitivity to disturbance (e.g., pesticides; Regan et al. 2017; Kautz and Gardiner 2019), Dolichopodidae show promise in serving as bioindicators of habitat quality and environmental change (Pollet 2009).

Like many fly families, the number of species of Dolichopodidae reaches its maximum in the New World tropics (Brown 2009; Borkent et al. 2018; Brown et al. 2018). However, dolichopodids in the New World tropics remain undersampled and understudied, with described faunas available for just a few areas (e.g., Robinson 1975; Pollet et al. 2018) and many regions having few or no recorded species at all. One region in which dolichopodids have received limited attention is the Lesser Antilles, part of the Caribbean Island Hotspot for biodiversity due to the high numbers of species and level of endemism for the available land area (Smith et al. 2005). The first report of Dolichopodidae in the Lesser Antilles came near the turn of the twentieth century, when J.M. Aldrich reported 46 species from St. Vincent (Aldrich 1896) and 55 species from Grenada (Aldrich 1902) based on material collected by H. H. Smith from 1889–1895 as part of a Royal Society project coordinated by the West India Exploration Committee. The dolichopodid fauna of Dominica is the most completely known in the Lesser Antilles due to the Bredin-Archbold-Smithsonian Biological Survey of Dominica (1960–1966). Several dipterists participated in this survey (e.g., R.J. Gagné, H. Robinson, G.C. Steyskal W.W. Wirth) and the Dolichopodidae were treated by Robinson (1975) and included 113 species in 30 genera. Since Robinson (1975), two genera and two species of Dolichopodidae have been added to the fauna of Dominica (Runyon 2015; Brooks and Cumming 2018), combined with the one genus and four species added herein brings Dominica’s count to 119 species in 33 genera. Aside from Grenada, St. Vincent, and Dominica, very few published records exist of dolichopodids on other Lesser Antillean islands (Van Duzee 1933a; Robinson 1975; Capellari 2015; Runyon and Capellari 2018). In fact, the islands with the next highest numbers of recorded dolichopodid species are Antigua (Robinson 1975) and St. Lucia (Van Duzee 1933a; Bickel 2002; Capellari 2015), each with just three documented species.

**Figure 1.**
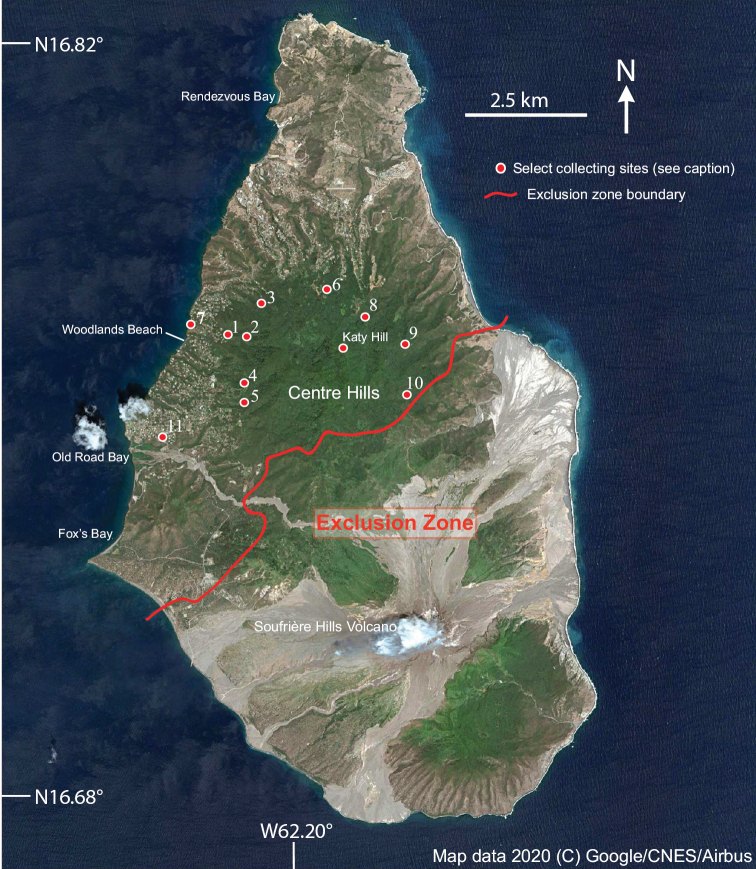
Map of Montserrat showing exclusion zone and primary collecting sites: 1–Cassava Ghaut, Beattie House; 2–Cassava Ghaut, canopy fogging site; 3–Fogarty Ghaut; 4–Gun Hill; 5–Hope Ghaut; 6–Underwood Ghaut; 7–Woodlands, Riverside House; 8– Bottomless Ghaut; 9–Jack Boy Hill; 10–Fairy Walk River; 11– Old Towne.

**Figure 2.**
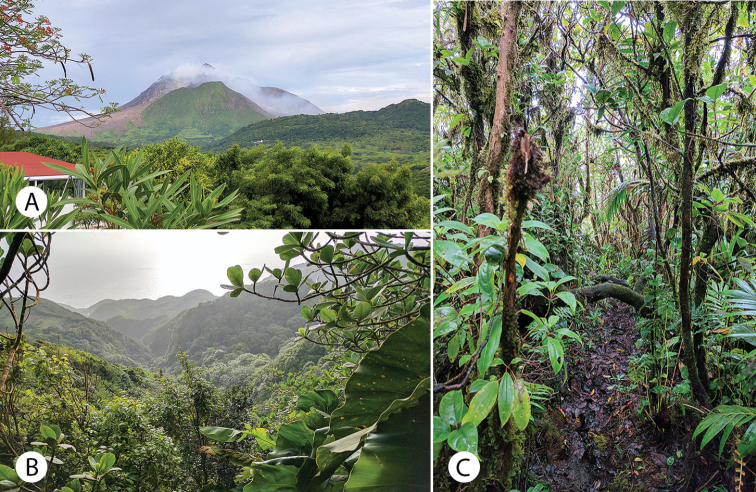
Some Montserrat landscapes and habitats **A** Soufrière Hills Volcano, view from Old Towne, June 2017 **B** northeast view from Katy Hill into Bottomless Ghaut **C** elfin woodland on top of Katy Hill, at the highest elevations of the Centre Hills. Photographs by Justin Runyon.

Montserrat (Figs 1, 2) is a small volcanic island near the northern end of the Lesser Antilles in the eastern Caribbean. It lies between 16°40' to 16°49'N latitude and 62°08' to 62°14'W longitude, with the closest large islands being Antigua (39 km to the northeast), Nevis (50 km to the northwest) and Basse-Terre (in the Guadeloupe archipelago, 55 km to the southeast). Montserrat is ca. 100 km^2^ in area and composed of three volcanic regions. These are (from North to South, with maximum elevation): the more arid Silver Hills (ca. 380 m), the Centre Hills (741 m) containing most of Montserrat’s high-quality and mature forests, and the Soufrière Hills Volcano (SHV)–South Soufrière Hills complex (ca. 1,050 m) (Harford et al. 2002). It is unclear when Montserrat emerged as an aerial land mass available for colonization, but estimates suggest between 4.3–2.6 Ma (Rea 1974; Harford et al. 2002). Montserrat has a moist tropical maritime climate with total annual rainfall varying from 1,100 mm at the coast to 2,100 mm at higher elevations (Young 2008; Hemmings et al. 2015). Geography is mostly steep with deeply incised radial valleys (locally called *ghauts*) that drain the higher elevations. However, despite high rainfall, there are no permanent rivers and streams and springs are predominantly ephemeral, due to the small size of the island and porous nature of the bedrock (Hemmings et al. 2015). Vegetation types transition from dry forest, mesic forest, wet forest, to elfin woodland as elevation increases (Young 2008). In 1995, the long-dormant Soufrière Hills volcano erupted, and activity has continued episodically since (Druitt and Kokelaar 2002). This volcanic activity destroyed a large proportion of vegetation on the southern half of the island including the highest elevations, resulting in establishment of an exclusion zone that rendered greater than half of Montserrat inaccessible (Fig. 1).

This study is based on material collected during the Centre Hills Biodiversity Assessment conducted from 2001–2005 (Young 2008) and material collected by the author in June 2017. Prior to this survey there were no published records of Dolichopodidae from Montserrat. A very preliminary list of dolichopodids from early work of this project was provided in the report by Ivie et al. (2008). Two dolichopodid species were recently reported from Montserrat, using material collected for this project: *Chimerothalassiusrunyoni* Brooks and Cumming (Brooks and Cumming 2018) and *Chrysotusxiphostoma* Robinson (Runyon and Capellari 2018). The purpose of this paper is to treat the dolichopodid fauna of Montserrat. Montserrat’s fauna is contrasted with Dominica’s, the only other island in the Lesser Antilles in which the dolichopodids have been intensively sampled and described. Lastly, collecting methods are compared and threats to the dolichopodid fauna of Montserrat discussed.

## Materials and methods

Specimens were collected during the Centre Hills Biodiversity Assessment (2001–2005) using Malaise traps, ultraviolet light traps, canopy fogging, and pan traps. Details of canopy fogging are given in Marske (2004). Several sites, mostly in mid-elevation forests of the Centre Hills, were intensively and repeatedly sampled, especially in 2002–2003 (Table 1; see Marske 2004; Ivie et al. 2008). In 2005, sampling focused on wetter (e.g., ghauts) and drier (e.g., coastal) habitats using Malaise traps, ultraviolet light traps, and pan traps. In June 2017, targeted sampling was done mostly with a net, but also using Malaise traps and pan traps.

Holotypes are deposited in the National Museum of Natural History, Smithsonian Institution, Washington, D.C. (**USNM**). Representatives of as many species as possible are also deposited in the USNM. All other specimens are deposited in the Montana Entomology Collection, Montana State University, Bozeman (**MTEC**), unless noted otherwise. Label data for primary types of new species are cited verbatim in quotation marks with lines separated by a slash (“/”), labels separated by a semicolon (“;”), and annotations in square brackets (“[]”). Label data for other material is presented in a standardized format, utilizing locality names for major sites presented in Table 1. Nearly all material derived from the Centre Hills Biodiversity Assessment from 2001–2005 (Young 2008) and material collected by the author in June 2017. In addition, several large collections (California Academy of Sciences, San Francisco; Canadian National Collection of Insects, Ottawa; Natural History Museum, London; National Museum of Natural History, Smithsonian Institution, Washington, D.C.) were searched for dolichopodid specimens from Montserrat. The only ones found were one specimen each of *Condylostyluslongicornis* (Fabricius) and *Thrypticusviolaceus* Van Duzee, both collected in 1910, in the USNM.

**Table 1.** Information on major Montserrat sampling localities during the Centre Hills Biodiversity Assessment (2001–2005). Locality names are used in Material examined sections.

Locality name	Latitude (N) / Longitude (W)	Elevation (m)	Habitat type
Cassava Ghaut, Beattie House	16°45.91'N, 62°12.95'W	193	dry forest
Cassava Ghaut, canopy fogging site	16°45.75'N, 62°12.47'W	263	mesic forest
Fogarty	16°46.235'N, 62°12.529'W	367	mesic forest
Gun Hill	16°45.4'N, 62°12.7'W	260	mesic forest
Hope Ghaut	16°45.169'N, 62°12.736'W	315	mesic forest
Underwood Ghaut	16°46.327'N, 62°11.734'W	369	mesic forest
Woodlands, Riverside House	16°45.985'N, 62°13.341'W	43	dry forest

To examine male terminalia using a compound microscope, for larger species the tip of the abdomen was cut off or for small species the entire specimen was removed from their pins by soaking in an approximately 50:50 mixture of 95% ethanol and ethyl acetate to dissolve shellac gel. These specimens were subsequently macerated in 85% lactic acid by heating in a microwave oven for one to three 15-second intervals, prior to being transferred to glycerin. Potassium hydroxide (20%) was additionally used to clear tergites of species in which terminalia are enclosed in tip of abdomen (e.g., most Diaphorinae). These specimens/genitalia were then transferred to plastic micro-tubes and placed on a pin or attached to the corresponding specimens.

Terminology used for adult structures follows McAlpine (1981) and Cumming and Wood (2009). In descriptions, the position of features on elongate structures, such as leg segments, is given as numerical fractions of the total length, starting from the base (e.g., seta at 1/3), but spelled out as words for proportions (e.g., “brown on apical two-thirds”). The male postabdomen on intact specimens is rotated approximately 180° and lateroflexed to the right, but in descriptions “dorsal” and “ventral” refer to the true morphological positions prior to genitalic rotation and flexion (e.g., in lateral view, top of the page is ventral while the bottom is dorsal). The relative lengths of the podomeres are representative ratios and not measurements. The following abbreviations and terms are used:

**ad** anterodorsal(ly);

**av** anteroventral(ly);

**pd** posterodorsal(ly);

**pv** posteroventral(ly).

Body segments are denoted using Roman numerals (e.g., tergite VI). Legs are designated by Roman numerals, tarsomeres by bracketed Arabic numerals (e.g., tarsus III(4) = 4^th^ tarsomere of hindleg).

Identification of species was accomplished using published keys and descriptions, especially Aldrich (1896), Aldrich (1902), and Robinson (1975), followed by: (1) examination of primary types; (2) comparison with material from Dominica and other islands; and (3) sending specimens to experts of particular groups: Achalcinae verified by Marc Pollet; Neurigoninae by Stefan Naglis; some *Chrysotus* by Renato Capellari; and *Chimerothalassius* by Scott Brooks and Jeff Cumming. Subfamilies are presented in order following Robinson (1975). Rarefaction was performed using iNext Online (Chao et al. 2016) a procedure that equalizes the sampling effort based on the number of specimens.

## Results

**Figure 3.**
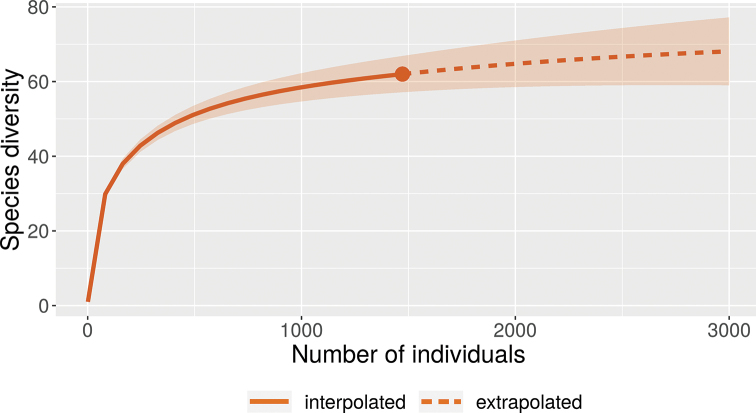
Rarefaction of species accumulation curve for Montserrat Dolichopodidae with 95% confidence interval and extrapolation to 2 × the numbers of individuals sampled in this study.

Approximately 1,500 dolichopodid specimens were collected during this study, representing 63 species in 27 genera. A list of Dolichopodidae species from Montserrat and current understanding of their distributions are presented in Table 2. A sampling curve illustrating the accumulation of unique species with the number of specimens collected approaches but does not reach an asymptote (Fig. 3), indicating that most of Montserrat’s dolichopodid species were discovered during this project.

**Table 2.** Species list of Dolichopodidae (Diptera) known from Montserrat, current understanding of distributional status, and occurrence on Dominica. Distribution status codes: IE – Island Endemic, Montserrat only; LAE – Lesser Antilles Endemic, Sombrero to Grenada including local endemics recorded from just a few islands; WIE – West Indian Endemic, not on mainland or only south Florida; WN – Widespread Native, West Indies and mainland (rankings follow Ivie et al. 2008).

Species	Distributional status	Dominica
**Parathalassiinae**		
*Chimerothalassiusrunyoni* Brooks & Cumming	LAE	x
**Sciapodinae**		
*Amblypsilopusluteus* (Robinson)	LAE	x
*Amblypsilopusmarskeae* sp. nov.	IE	
*Condylostylusalbiciliatus* (Van Duzee)	WIE	x
*Condylostyluslongicornis* (Fabricius)	WN	x
*Condylostylusnigripilosus* Robinson	LAE	x
*Condylostylusquadricolor* (Walker)	WN	x
**Neurigoninae**		
*Coeloglutusconcavus* Aldrich	WN	x
*Dactylomyiadecora* (Aldrich)	LAE	
*Neurigonafuscicosta* Robinson	LAE	x
*Viridigonathoracica* (Van Duzee)	WN	x
**Medeterinae**		
*Cryptopygiellamusaphila* Robinson	LAE	x
*Medeteracrassicauda* Robinson	WIE	x
*Medeteradominicensis* Robinson	LAE	x
*Medeteraiviei* sp. nov.	IE	
*Medeteramontserratensis* sp. nov.	IE	
*Medeterapseudonigripes* Robinson	LAE	x
*Systenusladonnae* sp. nov.	LAE	x
*Thrypticusabdominalis* (Say)	WN	x
*Thrypticusaequalis* Robinson	LAE	x
*Thrypticusmediofuscus* sp. nov.	LAE	x
*Thrypticusparvulus* Van Duzee	LAE	x
*Thrypticusviolaceus* Van Duzee	WN	x
**Achalcinae**		
*Xanthinarubromarginata* Robinson	LAE	x
**Enliniinae**		
*Enliniapatellitarsis* Robinson	LAE	x
*Harmstoniasimplex* Robinson	LAE	x
**Peloropeodinae**		
*Micromorphusalbipes* (Zetterstedt)	WN, Oriental, Palearctic	x
*Peloropeodesfrater* (Aldrich)	LAE	x
**Diaphorinae**		
*Achradoceraapicalis* (Aldrich)	WN	x
*Asyndetusinterruptus* (Loew)	WN	x
*Asyndetusfratellus* Aldrich	WIE	
*Chrysotusacutus* Aldrich	LAE	x
*Chrysotusalbihirtipes* Robinson	LAE	x
*Chrysotusangustifrons* (Robinson)	LAE	x
*Chrysotusantillensis* sp. nov.	WIE	x
*Chrysotusbrevicornis* Van Duzee	WN	x
*Chrysotuscallichromoides* sp. nov.	LAE	x
*Chrysotuscallichromus* Robinson	LAE	x
*Chrysotushirsutus* Aldrich	WN	x
*Chrysotusinterfrons* sp. nov.	IE	
*Chrysotusinteger* Robinson	LAE	x
*Chrysotuslamellicaudatus* Robinson	LAE	x
*Chrysotusmediocaudatus* Robinson	LAE	x
*Chrysotusmicrotatus* Meuffels & Grootaert	LAE	x
*Chrysotusmontserratensis* sp. nov.	IE	
*Chrysotusorichalceus* Gosseries	LAE	x
*Chrysotusparvulus* (Aldrich)	WIE	x
*Chrysotusproximus* Aldrich	LAE	x
*Chrysotuspseudoniger* Robinson	LAE	x
*Chrysotusspectabilis* (Loew)	WN	x
*Chrysotusspinipes* Van Duzee	WIE	x
*Chrysotusxiphostoma* Robinson	LAE	x
*Diaphoruscontiguus* Aldrich	WN	x
*Diaphorusrobinsoni* sp. nov.	LAE	x
*Symbolialinearis* (Aldrich)	LAE	x
**Plagioneurinae**		
*Plagioneurusunivittatus* Loew	WN	x
**Sympycninae**		
*Sympycnusmontserratensis* sp. nov.	IE	
*Sympycnuspentachaetus* Robinson	LAE	x
**Dolichopodinae**		
*Paracliusmegalocerus* Robinson	LAE	x
*Paraclius* sp. (female)	?	
*Tachytrechusperornatus* Robinson	LAE	x
**Hydrophorinae**		
*Cymatopusbredini* Robinson	LAE	x
*Thinophilusochrifacies* Van Duzee	WN	x

### Key to the genera of Dolichopodidae of Montserrat^1^

**Table:** 

1	Antenna with single articled arista-like stylus (Brooks and Cumming 2018: figs 3, 4); wing with crossvein dm-cu absent (Brooks and Cumming 2018: figs 8, 9); body size ca. 1.0 mm; rocky coastlines; PARATHALASSIINAE	***Chimerothalassius* Shamshev & Grootaert**
–	Antenna with two articled arista-like stylus; wing with crossvein dm-cu present; body size and habitat various	**2**
2	Vertex strongly excavated on either side of ocellar tubercle; vein M distinctly branched, with M_2_ present at least as a fold on membrane (as in Fig. 4D); Sciapodinae	3
–	Vertex not or scarcely excavated; vein M not branched, M_2_ absent	**4**
3	Frons with raised mound bearing strong vertical seta subtended by numerous shorter hairs; both pairs of scutellar setae long; antenna black	***Condylostylus* Bigot**
–	Vertical seta not arising on setose mound; lateral scutellar setae reduced and hair-like; antenna with some segments yellow	***Amblypsilopus* Bigot**
4	Veins R_4+5_ and M diverging from base to tip, with vein M ending distinctly behind wing tip (in some males vein M arches greatly backwards in apical third of wing); body size ca. 1.0 mm; ENLINIINAE	**5**
–	Veins R_4+5_ and M subparallel or converging beyond crossvein dm-cu (but diverging in males of *Systenusladonnae*, Fig. 14B); body size usually greater than 2.0 mm	**6**
5	Acrostichal setae absent; male wing and legs unmodified; females with small setae present on face above mouth	***Harmstonia* Robinson**
–	Acrostichal setae biseriate; males with tarsus I modified; female face without setae	***Enlinia* Aldrich**
6	Scape with setae on dorsal surface; male hypopygium enlarged and pedunculated, and projecting forward beneath abdomen; femur II and III with strong anterior preapical setae; all tibia with strong setae; posterior mesonotum not flattened; DOLICHOPODINAE	**7**
–	Without the above combination of characters	**9**
7	Dorsal and ventral hairs of arista-like stylus much longer than lateral hairs; vein M distinctly bent midway beyond dm-cu crossvein and joining margin near R_4+5_	***Pelastoneurus* Loew [not yet recorded from Montserrat]**
–	Arista-like stylus with all hairs subequal, or bare; venation various	**8**
8	Lower margin of face rounded, projecting medially and extending below eye level; vein M beyond dm-cu crossvein gradually approaching R_4+5_	***Tachytrechus* Haliday**
–	Lower margin of face more or less straight and not reaching lower eye margin; vein M abruptly approaching R_4+5_ beyond dm-cu crossvein	***Paraclius* Loew**
9	Posterior mesonotum distinctly flattened and slight depressed, from one-third to one-half of surface between dorsocentral setae, and distinct from concave anterior mesonotum	**10**
–	Posterior mesonotum not flattened, or at most only slightly or apparently flattened immediately anteriad of scutellum	**21**
10	Femur II and III without major anterior preapical seta; dorsal postcranium usually distinctly concave	**11**
–	Femur II and III with distinct anterior or anterodorsal preapical seta; dorsal postcranium various	**19**
11	Body and legs covered with dense gray tomentum, usually obscuring cuticle; mesonotum strongly arched with posterior slope flattened but not concave and with weak margin; acrostichal setae absent; scutellum with 2 (rarely 3) slender setae per side lateral to pair of larger setae; male forefemur with row of stout anteroventral setae on basal half; rocky coastlines; HYDROPHORINAE, in part	***Cymatopus* Kertész**
–	Body tomentum usually not dense, and underlying cuticle visible; mesonotum usually strongly flattened to slightly concave with distinct margin; scutellum with 0 or 1 hair or seta per side lateral to pair of larger setae; not restricted to intertidal areas; other features various	**12**
12	First flagellomere globular, nearly round in anterior view; hypopygium completely enclosed by abdomen (Fig. 6B)	***Cryptopygiella* Robinson**
–	First flagellomere compressed laterally, subrectangular or flattened in anterior view; hypopygium external and usually distinctly pedunculated, at most partially hidden	**13**
13	Arista-like stylus apical; male genitalic capsule ovate to pyriform on a peduncle formed by exserted haired segment VII, and not encapsulated or enfolded by preceding abdominal segments; male abdominal segments IV and V unmodified; vein M various; face often metallic; MEDETERINAE	**14**
–	Arista-like stylus dorsal or subapical; male genitalic usually globular, on a peduncle formed by short bare segment VII, and sometimes enfolded by preceding abdominal segments; male abdominal segments IV and/or V sometimes with ventral modifications; vein M beyond dm-cu crossvein with flexion or depression (*bosse alaire*) in membrane; face with dense pruinosity; NEURIGONINAE	**16**
14	Male antenna with first flagellomere abruptly narrowed to elongate tapering point in distal half (Fig. 14C); female first flagellomere subovate (Fig. 14D); proepisternum with single pale seta; M beyond dm-cu crossvein with flexion; 6 strong dorsocentral setae	***Systenus* Loew**
–	Postpedicel of male and female similar, not elongate; proepisternum bare; M beyond dm-cu crossvein without flexion; dorsocentral setae usually 5 or fewer	**15**
15	Wing vein M distinctly curving towards R_4+5_ beyond dm-cu crossvein; vein A_1_ weak but distinct; hind coxa with 1 lateral seta; 2 supra-alar setae present, posterior seta stronger than anterior; femur II without strong posterior subapical seta; female oviscapt not forming a sclerotized, bladelike piercing structure	***Medetera* Fischer von Waldheim**
–	Wing with veins M and R_4+5_ subparallel to apex; vein A_1_ absent; hind coxa with 2 lateral setae; only 1 supra-alar seta present; femur II with strong posterior subapical seta; female oviscapt sclerotized, bladelike and laterally compressed	***Thrypticus* Gerstäcker**
16	Thorax strongly elongated; abdomen approximately as long as thorax, dorsoventrally flattened; vertex excavated dorsally laterad of ocellar tubercle; tarsus I(5) with ventral comb of short spines; arista-like stylus subapical; hypopygium small, partially enclosed by segment V or VI	***Coeloglutus* Aldrich**
–	Thorax not elongated; abdomen usually longer than thorax, cylindrical; vertex not excavated; tarsus I(5) unmodified; arista-like stylus dorsal; hypopygium large, external	**17**
17	Vein M S-shaped, joining costa before wing apex and close to R_4+5_, with costal difference between veins less than half length of crossvein dm-cu; tibiae II and III bare of major setae; hypopygium yellow; female oviscapt with cercus rounded and free from tergites IX+X	***Dactylomyia* Loew**
–	Vein M straight or slightly bent, joining costa near or behind wing apex, with costal difference between vein M and R_4+5_ greater than half length of crossvein dm-cu; tibia II and/or III with major setae; hypopygium black; female oviscapt with cercus digitiform and fused to tergites IX+X	**18**
18	Thorax metallic green-blue; tarsus I(4) slightly compressed; wing hyaline	***Viridigona* Naglis**
–	Thorax mostly yellowish, metallic blue-green on only mesonotal depression and scutellum; tarsus I(4) not compressed; wing brownish anteriorly, especially in males	***Neurigona* Rondani**
19	Acrostichal setae biseriate; body mostly yellow; pedicel overlapping first flagellomere medially; male palpi modified with reddish apical margin; hypopygium not enlarged; ACHALCINAE	***Xanthina* Aldrich**
–	Acrostichal setae uniseriate or totally absent; body usually dark colored; pedicel truncate, not overlapping first flagellomere; male palpus unmodified; hypopygium various but often enlarged; PELOROPEODINAE	**20**
20	Acrostichal setae totally absent; hypopygium subrectangular, free from abdomen; body size 1.0–1.5 mm	***Micromorphus* Mik**
–	Acrostichal setae uniseriate; hypopygium swollen and globular, encapsulated at abdominal apex; tarsus I(5) with one slightly enlarged, appressed claw; body size 2.0–2.5 mm	***Peloropeodes* Wheeler**
21	Pair of large postvertical setae present on dorsal postcranium, out of line with postorbital series; abdomen dorsoventrally flattened; face and enlarged subquadrate palpi golden; coastal areas; HYDROPHORINAE, in part	***Thinophilus* Wahlberg**
–	Postvertical setae, if present, near vertex; abdomen usually ovate, and rarely dorsoventrally flattened; palpus usually small, but sometimes enlarged in male only; male face often narrowed	**22**
22	Face with vertical median furrow; crossvein dm-cu oblique, parallel to last part of M; male abdominal sternites III and IV with strong submarginal setae; acrostichal setae absent; thorax metallic green with coppery band; arista-like stylus dorsal and first flagellomere pointed triangular; PLAGIONEURINAE	***Plagioneurus* Loew**
–	Face without median furrow; crossvein dm-cu not parallel to last part of M; abdominal sternites III and IV without obvious large setae; other characters various	**23**
23	Femur II and/or III with distinct anterior or anterodorsal preapical seta; SYMPYCNINAE	***Sympycnus* Loew**
–	Femur II and/or III without distinct anterior preapical seta (but sometimes with larger av setae near apex), or such apparent preapicals indistinct from background setal field; DIAPHORINAE	**24**
24	Scape with dorsal setae	***Symbolia* Becker**
–	Scape without dorsal setae	**25**
25	Upper part of proepisternum with 1 or more small setae; male face parallel-sided; female with narrowest part of face subequal in width to widest part of frons; males often with enlarged pulvilli that are fused with claws on foreleg; male tergite VI bare, mostly or completely hidden; male sternite VIII with 4 strong projecting setae	**26**
–	Upper part of proepisternum bare; male face narrowed below or parallel-sided; female with narrowest part of face narrower than widest part of frons; males rarely with enlarged pulvilli fused with claws; male tergite VI setose or at least with 1 distolateral seta at lower margin, mostly exposed; setae on male sternite VIII not or scarcely stronger than those on tergite VI	**27**
26	Costa not extending beyond tip of R_4+5_; distal vein M weakened or broken, usually with distal section displaced; calypter with pale setae; male frons wide, eyes not dorsally holoptic	***Asyndetus* Loew**
–	Costa ending at apex of vein M; vein M unbroken; calypter with black setae; male eyes dorsally holoptic	***Diaphorus* Meigen**
27	Male first flagellomere with slender apical projection bearing essentially apical arista-like stylus; lower postocular surface with many flattened pale setae; femur III wholly brown, femora I and II mostly yellow and usually narrowly brownish along dorsal edge	***Achradocera* Becker**
–	Male first flagellomere with arista-like stylus subapical in notch or to side of tip; lower postocular surface with fine pale setae, not flattened; color of femora various	***Chrysotus* Meigen**

## Systematic List

### Subfamily Parathalassiinae

#### Genus *Chimerothalassius* Shamshev & Grootaert

##### Chimerothalassius runyoni

Taxon classificationAnimaliaDipteraDolichopodidae

Brooks & Cumming

63B83532-34CF-5591-8654-1A2A0B05529B

Chimerothalassius runyoni Brooks & Cumming, 2018: 513.

###### Material examined.

**Montserrat**: 4 ♂, 13 ♀, Woodlands Beach, rocks in intertidal zone, 16°45.817'N, 62°13.384'W, 20–22 June 2017, JB Runyon. Specimens deposited in the Canadian National Collection of Insects, Ottawa.

###### Distribution.

Dominica, Montserrat.

###### Remarks.

*Chimerothalassius* belongs to the subfamily Parathalassiinae which is considered the sister group to the Dolichopodidae*sensu stricto* (Sinclair and Cumming 2006; Brooks and Cumming 2011). *Chimerothalassiusrunyoni* was known from one female specimen and a slide-mounted wing collected in Dominica during the Bredin-Archbold-Smithsonian Biological Survey and was formally described after males were collected on Montserrat in 2017 (Brooks and Cumming 2018). This species occurs on rocky or stony intertidal zones.

### Subfamily Sciapodinae

#### *Amblypsilopus* Bigot

##### Key to the species of *Amblypsilopus* in Montserrat

**Table:** 

1	Thorax mostly yellow with mid-dorsal metallic blue-green stripe; male costa with long S-shaped cilia	***A.luteus* (Robinson)**
–	Thorax entirely metallic blue-green; male costa without long cilia	***A.marskeae* sp. nov.**

##### Amblypsilopus luteus

Taxon classificationAnimaliaDipteraDolichopodidae

(Robinson)

F430420C-BB9A-55B3-93C7-FCF0ED49718C

Sciapus luteus Robinson, 1975: 16.

###### Material examined.

**Dominica: *Holotype*** ♂, Clarke Hall, 17 February 1964, H. Robinson (USNM). **Montserrat**: 5 ♂, 5 ♀, Woodlands, Riverside House, 10–12 January 2002, Malaise trap, Ivie, Marske & Puliafico; 1 ♀, same as previous, 5–7 January 2002; 15 ♂, 2 ♀, Cassava Ghaut, Beattie House, 14–21 January 2002, Malaise trap, A. Krakower; 2 ♂, 1 ♀, same as previous, 21 January–5 February 2002; 2 ♂, 1 ♀, same as previous, 5–15 February 2002; 1 ♂, same as previous, 18 March–4 April 2002; 4 ♂, 5 ♀, same as previous, 14–30 June 2002, M.A. Ivie; 1 ♂, same as previous, 30 June–4 July 2002; 3 ♂, Cassava Ghaut, canopy fogging at dawn, 4 February 2003, L. Martin & J. Boatswain; 1 ♂, Gun Hill, 18–30 May 2002, Malaise trap, K.A. Marske; 1 ♂, 2 ♀, same as previous, 30 May–7 June 2002; 2 ♂, 2 ♀, same as previous, 2–19 June 2002; 2 ♂, 1 ♀, Cassava St., Burty House, 13–14 January 2002, UV light trap, M.A. Ivie & K. Marske; 2 ♂, Fogarty, 20–22 June 2002, Malaise trap, K.A. Marske; 3 ♂, Hope Ghaut, canopy fogging at dawn, 4 December 2002, J. Boatswain & J. Martin; 1 ♀, Hope Ghaut, 8–10 January 2002, yellow pan trap, K.A. Markse; 1 ♂, Underwood Ghaut, canopy fogging at dawn, 23 May 2002, K. Marske & J. Boatswain; 1 ♂, trail to Fairy Walk, 15 August 2005, yellow pan, V.G. Martinson; 1 ♂, on roadside vegetation, 16°46.06'N, 62°13.10'W, 19 June 2017, J.B. Runyon; 1 ♀, Woodlands Beach, 16°45.75'N, 62°13.42'W, 20 June 2017, J.B. Runyon; 5 ♂, Hope Ghaut, 300 m, 16°45.108'N, 62°12.695'W, 20 June 2017, J.B. Runyon; 2 ♂, 1 ♀, Fogarty Ghaut (Soldiers), 16°46.41'N, 62°12.44'W, 21 June 2017, J.B. Runyon; 1 ♀, Jack Boy Hill (top), 480 m, 16°45.797'N, 62°10.886'W, 25 June 2017, J.B. Runyon; 2 ♂, Fairy Walk River, 260 m, 16°45.162'N, 62°10.854'W, 26 June 2017, J.B. Runyon (MTEC, USNM).

###### Distribution.

Dominica, Montserrat.

###### Remarks.

Adults occur on vegetation but were also commonly found hopping around on dead leaves and rocks on the forest floor.

##### Amblypsilopus marskeae

Taxon classificationAnimaliaDipteraDolichopodidae

sp. nov .

607E8D92-8C3B-5DF2-89F0-9EC8572E78B7

http://zoobank.org/92E7384D-FB10-4479-AE9C-0AC3737E9E6B

Figs 4 , 5

###### Type material.

***Holotype***, ♂ labelled: “WEST INDIES: MONTSERRAT/ rental house in Old Town/ 16°44.795'N, 62°13.711'W/ 19 JUNE 2017, JB Runyon”; “HOLOTYPE/ ♂ *Amblypsilopus*/ *marskeae*/ Runyon [red label]” (USNM, type number USNMENT01350607). ***Paratypes*: Montserrat**: 4 ♂, 5 ♀, same data as holotype; 1 ♂, Woodlands, Riverside House, 5–7 January 2002, Malaise trap, Ivie, Marske & Puliafico; 1 ♂, same as previous, 10–12 January 2002 (MTEC).

###### Description.

**Figure 4.**
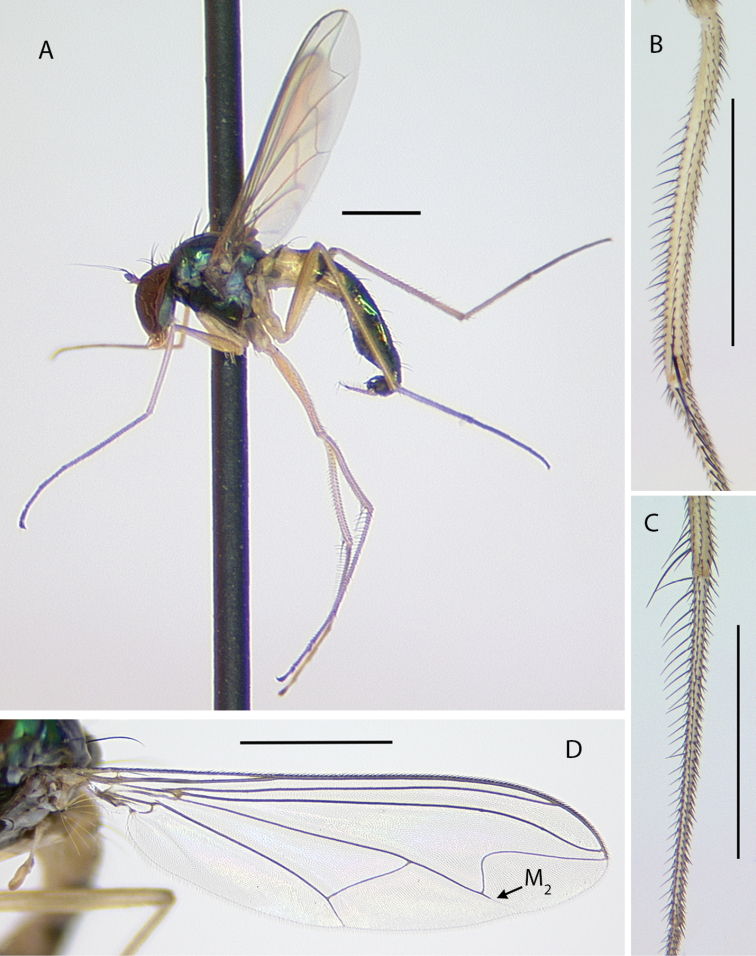
*Amblypsilopusmarskeae* sp. nov. males **A** habitus of holotype, left lateral **B** tibia II and base of tarsus II(1), anterodorsal view **C** tip of tibia II and tarsus II(1), dorsal **D** wing, dorsal. Scale bars: 1 mm.

**Figure 5.**
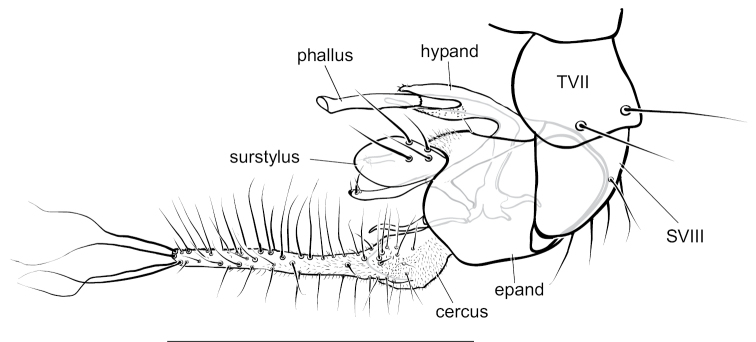
*Amblypsilopusmarskeae* sp. nov. male terminalia, left lateral. Abbreviations: epand-epandrium; hypand-hypandrium; TVII-tergite 7; SVIII-sternite 8. Scale bar: 0.5 mm.

**Male** (Fig. 4). Body length 4.5–5.0 mm, wing length 4.0–4.5 × width 1.1–1.3 mm. ***Head***: Face somewhat narrowed below, three-fifths as wide at frontoclypeal suture as at antenna; face and frons metallic blue-green with violet reflections, with white pruinosity that is coarser and denser on lower face. Palpus yellow, rather narrow and pointed apically, with short yellow setae and two black setae (near 1/2 and at apex). Proboscis yellow, projecting anteriorly, with a few yellow hairs. Antenna with scape and pedicel yellow; first flagellomere brown, ovate-triangular, a little longer than wide, with arista-like stylus inserted near middle of dorsal edge. Lower postocular setae white. ***Thorax***: Scutum and scutellum metallic green with some violet reflections, covered with slight white pruinosity; five pairs of biseriate acrostichal setae, the posterior-most pair much larger (subequal in size to largest dorsocentral setae); five or six pairs of dorsocentral setae, posterior two pairs largest; scutellum with one pair of large marginal setae and one pair of very small lateral setae. Pleuron metallic green with dense white pruinosity. ***Legs***: Coxa I entirely yellow with yellow hairs and three strong yellow distolateral setae; coxa II brown on outer surface with yellow anterior hairs and yellow apical setae, without lateral seta; coxa III entirely yellow with large yellow dorsal seta near 1/2 and a few yellow hairs. Remainder of legs yellow, except distal tarsomeres brownish. Femora unmodified, except femur II has slightly longer rows of black setae pd and posteriorly on apical half; femora lacking anterior preapical setae. Tibia I with small ad seta at 1/5; tibia II (Fig. 4B, C) slightly sinuous, with row of 4–5 ad setae near apex rapidly increasing in size distally (longest 3 × width of tibia), with row of erect av setae on distal 2/3, longest setae in this row near middle and slightly longer than width of tibia, with pv row of very short erect setae, with 3–4 outstanding ventral setae on basal one-fourth (longest subequal to width of tibia); tibia III with ad seta near 1/5. Tarsus II(1–3) with ad row of erect setae, those at base of tarsus II(1) longest (length ca. 3 × width of tibia) and gradually decreasing in size distally (Fig. 4C), tarsus II(2–3) with 2–3 slightly longer setae in this row near apex and with rather long black distally directed posterior seta at apex; tarsus II (3–4) very slightly swollen at base bearing small tuft of short black posterior setae; tarsus III(1) with 2–3 black setae ventrally at base. Ratios of tibia:tarsomeres: leg I: 14–18–5–4–2–1; leg II: 20–22–6–5–3–2; leg III: 26–14–6–4–2–1. ***Wing*** (Fig. 4D): Hyaline, narrowly oblong-elliptical; costa without obvious special cilia or setae, but with very short (length subequal to width of costa), erect, fine cilia scattered along costa from just before apex of R_1_ to near apex of R_2+3_, the position of these minute cilia seems to correspond to the longer, curved costal cilia found in other *Amblypsilopus* species (e.g., *A.bredini* Robinson); vein M branched, M_1_ strongly arched anteriorly just beyond branch and ending in wing apex; M_2_ fading and not reaching wing margin; crossvein dm-cu nearly 3 × as long as last part of CuA_1_. Calypter yellow with edge narrowly black, with fan of long yellow setae. Halter yellow. ***Abdomen***: Narrow, cylindrical. Tergite I yellow with posterolateral edge narrowly brown, with yellow hairs and longer black setae on posterior margin; remainder of tergites with black hairs and setae, those near posterior margins larger; tergite II entirely yellow on basal one-fourth and on most of lateral surface, brown with metallic green reflections dorsally and along posterior margin; tergites III and IV mostly yellow laterally and brown with metallic green reflections dorsally and along posterior margins; tergites V–VII entirely metallic green; tergite VII mostly bare except four large setae along posterior margin. Sternites I–IV yellow with sparse yellow hairs; sternites V and VI brown with mostly dark hairs and setae; sternite VII reduced to a sclerotized band attached to ovate setose sternite VIII which covers the hypopygial foramen. Hypopygium (Fig. 5) rather small, on a short, broad peduncle formed by tergite VII. Hypopygial foramen at base on left side. Epandrium brown, a little longer than wide, rather square apically, covered with white microtrichia that is densest at ventroapical corner. Surstylus shining dark brown, bilobed, with large oval ventral lobe bearing four large setae and small digitiform medial lobe with three small setae near apex; dorsal lobe of surstylus slender, finger-like with three ventral setae near apex. Cercus yellow, elongate cylindrical but broader basally, covered with abundant setae (color of setae varies from yellow to dark brown) especially along ventral surface, and with four long black wavy setae at apex. Phallus broad, slightly flared at apex with large opening, with sclerotized articulation near 2/3, near where phallus emerges from hypandrium. Hypandrium sclerotized dark brown, asymmetrical, with broad ventral hood bearing hairs at pointed apex and left lateral lobe arising near base that crosses dorsally to right side of phallus, this lobe has minute scale-like setae on apical half. Subepandrial sclerite with sharply pointed, slightly hooked process emerging just ventral to base of cercus.

**Female.** Body length 4.5–5.0 mm, wing length 4.0–4.5 × width 1.3–1.5 mm. Similar to male, but clypeus more distinct and bulging; three yellow distolateral setae on coxa I stronger than in male; leg II unmodified, tibia II with small ad and pv seta near 1/5 and smaller pv seta near 1/2; wing noticeably broader.

###### Etymology.

This species is named to honor Dr. Katharine A. Marske (University of Oklahoma). Many specimens used in this study were collected as part of Katie’s Master’s thesis at Montana State University examining the effects of volcanic ash on Montserrat forest insects (Marske 2004), a component of the Centre Hills Biodiversity Assessment.

###### Distribution.

Montserrat.

###### Remarks.

*Amblypsilopusmarskeae* belongs to the New World group of *Amblypsilopus* species that possess costal cilia (which are poorly developed in *A.marskeae*) and three strong distolateral setae on coxa I that are more strongly developed in females. *Amblypsilopusmarskeae* is closely related to *A.bredini* (Robinson) from Dominica which has a similarly sinuous tibia II but differs most notably in color of the thorax and males lacking long, hooked cilia on the costa. Specimens were collected at the type locality from a shaded, vertical surface of a roadside concrete wall and on low vegetation in an adjacent small ghaut.

#### Genus *Condylostylus* Bigot

##### Key to the species of *Condylostylus* in Montserrat

**Table:** 

1	Wing clear or slightly clouded along anterior margin	**2**
–	Wing with 2 transverse brown bands that are joined anteriorly	**3**
2	Tibia III with only distal one-third brown; tibiae I and II with long setae; femur III with long dense wavy-tipped white hairs posteriorly; tibia II and tarsus II(1) without row of distinctive setae	***C.albiciliatus* (Van Duzee)**
–	Tibia III wholly brown; tibiae I and II with longest setae only twice as long as tibial width; femur III with straight white hairs posteriorly only a little longer than femoral width; tibia II with ca. 12 short setae in av row; tarsus II(1) with series of slender erect cilia along anterior surface	***C.longicornis* (Fabricius)**
3	Clypeus as wide or wider than long; male with numerous black hairs along middle of scutum; hypopygium and cerci large; female femora black; knob of halter dark brown in both sexes	***C.nigripilosus* Robinson**
–	Clypeus width less than length; male without black hairs on scutum; hypopygium and cerci small; female femora yellow; knob of halter yellow brown in both sexes	***C.quadricolor* (Walker)**

##### Condylostylus albiciliatus

Taxon classificationAnimaliaDipteraDolichopodidae

(Van Duzee)

F3EB32BC-4D2F-527E-8D6D-FF26DFD452FB

Psilopus albiciliatus Van Duzee, 1927: 9.
Condylostylus perpilosus Robinson, 1975: 8.

###### Material examined.

**Dominica: *Holotype*** ♂ of *Condylostylusperpilosus*, Clarke Hall, 11–20 January 1965, W.W. Wirth (USNM). **Montserrat**: 3 ♂, 3 ♀, Woodlands, Riverside House, 5–7 January 2002, Malaise trap, Ivie, Marske & Puliafico; 2 ♂, 7 ♀, same as previous, 10–12 January 2002; 3 ♂, same as previous, yellow pan, 8–10 January 2002, K. Marske & K. Puliafico; 8 ♂, 1 ♀, Cassava Ghaut, Beattie House, 15–18 February 2002, light trap, A. Krakower; 1 ♂, same as previous, 11–23 March 2002, UV light; 2 ♂, same as previous, 6–12 June 2002; 3 ♂, 1 ♀, same as previous, 21–30 June 2002, M.A. Ivie; 4 ♂, 3 ♀, Cassava Ghaut, Beattie House, 14–21 January 2002, Malaise trap, A. Krakower; 5 ♂, 1 ♀, same as previous, 21 January–5 February 2002; 1 ♂, 2 ♀, same as previous, 5–15 February 2002; 2 ♂, same as previous, 4–23 March 2002; 2 ♂, same as previous, 18 March–4 April 2002; 1 ♂, 1 ♀, same as previous, 8–17 April 2002; 1 ♂, 1 ♀, same as previous, 17–30 May 2002; 1 ♂, 4 ♀, same as previous, 14–30 June 2002, M.A. Ivie; 1 ♂, Cassava Ghaut, canopy fogging at dawn, 21 May 2002, K. Marske & J. Boatswain; 4 ♂, trail to Fairy Walk, 15 August 2005, yellow pan trap, V.G. Martinson; 1 ♂, Hope Ghaut, canopy fogging at dawn, 4 December 2002, J. Boatswain & J. Martin; 1 ♂, Fogarty, canopy fogging at dawn, 10 October 2002, J. Daley & J. Martin; 1 ♂, Sweetwater Ghaut, 1 August 2005, yellow pans, V.G. Martinson; 1 ♂, Cassava St., Burty House, 13–14 January 2002, UV light trap, M.A. Ivie & K. Marske; 1 ♂, Killiekranke, 3 August 2005, yellow pan trap, V.G. Martinson; 2 ♂, on roadside vegetation, 16°46.06'N, 62°13.10'W, 19 June 2017, J.B. Runyon; 1 ♂, Hope Ghaut, 280 m, 16°45.101'N, 62°12.760'W, 20 June 2017, J.B. Runyon (MTEC, USNM).

###### Distribution.

West Indies (Dominica, Jamaica, Montserrat, Puerto Rico, St. Lucia, Virgin Islands).

###### Remarks.

This species is widespread in the West Indies, being described using material from Jamaica, Puerto Rico, and the Virgin Islands (Van Duzee 1927) and later reported from Dominica (Robinson 1975) and St. Lucia (Bickel 2002). Robinson (1975) described this species as *Condylostylusperpilosus* which was synonymized with *C.albiciliatus* by Bickel (2002). Adults of *C.albiciliatus* appear to be active year-round on Montserrat.

##### Condylostylus longicornis

Taxon classificationAnimaliaDipteraDolichopodidae

(Fabricius)

44E2C8AD-DA2A-500E-B498-C2F8EC1BBAAA

Musca longicornis Fabricius, 1775: 783.
Psilopus radians Macquart, 1834: 450.
Psilopus nigripes Macquart, 1842: 181.
Psilopus flavimanus Macquart, 1842: 182.
Psilopus chrysoprasi Walker, 1848–1849: 646.
Psilopus metallifer Walker, 1848–1849: 647.
Psilopus zonatulus Thomson, 1869: 509.
Psilopus trichosoma Bigot, 1890: 285.
Psilopus ciliipes Aldrich, 1901: 355.
Condylostylus dentaticauda Van Duzee, 1933b: 66.

###### Material examined.

**Dominica**: 3 ♂, 2 ♀, near Layou, 27 January–12 February 1964, H. Robinson (USNM); 2 ♂, 1 ♀, Springfield Estate, yellow pans, 1–3 June 2011, M.A. & L.L. Ivie. **Montserrat**: 13 ♂, 9 ♀, Woodlands, Riverside House, 10–12 January 2002, Malaise trap, Ivie, Marske & Puliafico; 4 ♂, 5 ♀, same as previous, 5–7 January 2002; 5 ♂, 4 ♀, trail to Fairy Walk, 15 August 2005, yellow pan trap, V.G. Martinson; 1 ♂, Sweetwater Ghaut, 1 August 2005, yellow pans, V.G. Martinson; 1 ♂, 1910, HAB [remainder of label illegible] (MTEC, USNM).

###### Distribution.

*Condylostyluslongicornis* is widespread in the New World tropics and subtropics and has been readily transported by humans to other biogeographic realms. Recorded from the southeastern USA, Caribbean, Central America, tropical South America (including Galápagos Islands), and introduced to French Polynesia and Hawaii (Bickel 2002), Australia, China, India, Indonesia, Papua New Guinea, Philippines, and Sri Lanka (Yang et al. 2006), and United Arab Emirates (Naglis and Bickel 2017).

###### Remarks.

Robinson (1975) treated this species as *Condylostyluschrysoprasi* (Walker). The synonymy is from Bickel (2002).

##### Condylostylus nigripilosus

Taxon classificationAnimaliaDipteraDolichopodidae

Robinson

0C921632-3303-5AD3-9FB0-114E70508853

Condylostylus nigripilosus Robinson, 1975: 11.

###### Material examined.

**Dominica: *Holotype*** ♂, Clarke Hall, 15–19 April 1966, R.J. Gagné (USNM). **Montserrat**: 1 ♂, ♀, Cassava Ghaut, yellow pan trap, 24 July 2005, V.G. Martinson; 1 ♀, Cedar Ghaut, yellow pan trap, 4 August 2005, V.G. Martinson & D. Hughley (MTEC, USNM).

###### Distribution.

Dominica, Montserrat.

###### Remarks.

The male specimen from Montserrat has more of the hairs on the frons and femora white (which are mostly black in Dominica specimens), but otherwise it matches the holotype of *C.nigripilosus*.

##### Condylostylus quadricolor

Taxon classificationAnimaliaDipteraDolichopodidae

(Walker)

8C194886-6F22-5B8A-8425-FBE60BEA4EBB

Psilopus quadricolor Walker, 1848–1849: 649.
Psilopus jucundus Loew, 1861: 87, 88; 1864: 258﻿–260.
Psilopodinus astequinus Bigot, 1888: xxx.
Psilopus similis Aldrich, 1901: 359.
Sciapus digitatus Van Duzee, 1914: 391.
Condylostylus nigritibia Van Duzee, 1932: 183.

###### Material examined.

**Dominica**: 2 ♂, Springfield Estate, yellow pans, 1–3 June 2011, M.A. & L.L. Ivie. **Montserrat**: 2 ♂, Cassava Ghaut, 25 July 2005, yellow pan trap, V.G. Martinson; 1 ♂, trail to Fairy Walk, 15 August 2005, yellow pan trap, V.G. Martinson; 1 ♀, Cassava Ghaut, Beattie House, Malaise, 21 January–5 February 2002, A. Krakower; 1 ♀, same as previous, 17–30 May 2002; 1 ♂, 3 ♀, Woodlands, Riverside House, 10–12 January 2002, Malaise trap, Ivie, Marske & Puliafico (MTEC, USNM).

###### Distribution.

Widespread in the Neotropics.

###### Remarks.

Robinson (1975) treated this species as *Condylostylussimilis* (Aldrich), synonymized by Bickel (2002).

### Subfamily Neurigoninae

#### Genus *Coeloglutus* Aldrich

##### Coeloglutus concavus

Taxon classificationAnimaliaDipteraDolichopodidae

Aldrich

6EBCA9A1-68FC-5232-AC50-8D46D0A59108

Coeloglutus concavus Aldrich, 1896: 338.
Medetera sinuata Parent, 1928: 159.
Coeloglutus bicoloripes Van Duzee, 1933a: 15.

###### Material examined.

**Montserrat**: 2 ♂, 1 ♀, Cassava Ghaut, canopy fogging at dawn, 21 May 2002, K. Marske & J. Boatswain; 1 ♀, Cassava Ghaut, Malaise trap, 4–23 March 2002, A. Krakower; 1 ♂, 2 ♀, Fogarty Ghaut, canopy fogging, 6 December 2002, J. Daley & L. Aymer (MTEC, USNM).

###### Distribution.

West Indies (Puerto Rico, Dominica, Montserrat, St. Vincent) and from Guatemala south to Bolivia (Naglis 2001).

###### Remarks.

Naglis (2001) provided a re-description and illustration of *C.concavus*.

#### Genus *Dactylomyia* Loew

##### Dactylomyia decora

Taxon classificationAnimaliaDipteraDolichopodidae

(Aldrich)

9C20336C-CFCB-54E6-8F39-5A2A87E49360

Neurigona decora Aldrich, 1902: 83.

###### Material examined.

**Montserrat**: 1 ♂, Cassava Ghaut, Beattie House, 11–23 March 2002, UV light, A. Krakower; 1 ♂, same as previous, 21–30 June 2002, M.A. Ivie; 1 ♂, same as previous, Malaise trap, 8–17 April 2002, A. Krakower; 1 ♂, same as previous, 4–11 March 2002, M.A. Ivie & K.A. Marske; 2 ♀, rental house in Old Town, 16°44.795'N, 62°13.711'W, 19 June 2017, J.B. Runyon (MTEC, USNM).

###### Distribution.

Lesser Antilles (Barbados, Grenada, Montserrat, St. Vincent).

###### Remarks.

*Dactylomyiadecora* was re-described and illustrated by Naglis (2002). The 2017 specimens were collected from the trunk of an ornamental flame tree, *Delonixregia*, in a residential yard.

#### Genus *Neurigona* Rondani

##### Neurigona fuscicosta

Taxon classificationAnimaliaDipteraDolichopodidae

Robinson

B6D905D8-5093-5C23-B537-CE089467F4AE

Neurigona fuscicosta Robinson, 1975: 23.

###### Material examined.

**Montserrat**: 1 ♂, Cassava Ghaut, Beattie House, 18 March–4 April 2002, Malaise trap, A. Krakower; 1 ♂, same as previous, 8–17 April 2002; 2 ♂, same as previous, 17–30 May 2002; 2 ♂, same as previous, 14–30 June 2002, M.A. Ivie; 1 ♂, Gun Hill, 30 May–7 June 2002, Malaise trap, K.A. Marske (MTEC, USNM).

###### Distribution.

Dominica, Montserrat.

###### Remarks.

*Neurigonafuscicosta* was re-described and illustrated by Naglis (2003).

#### Genus *Viridigona* Naglis

##### Viridigona thoracica

Taxon classificationAnimaliaDipteraDolichopodidae

(Van Duzee)

6B15D589-294B-515A-AE52-8308AD700D3B

Neurigona thoracica Van Duzee, 1931a: 178.

###### Material examined.

**Dominica**: 1 ♂, St. David Parish, ca. 1 km NE Ponte Casse, Waitukubuli National Trail, 15.381490N, 61.340138W, Malaise trap, 31 May–5 June 2011. **Montserrat**: 1 ♂, Gun Hill, 18–30 May 2002, Malaise trap, K.A. Marske; 1 ♂, same as previous, 30 May–7 June 2002; 1 ♀, Cassava Ghaut, Beattie House, 4–23 March 2002, Malaise trap, A. Krakower; 1 ♂, same as previous, 17–30 May 2002; 1 ♂, same as previous, 14–30 June 2002, M.A. Ivie; 1 ♀, ghaut above Montserrat Volcano Observatory, 330 m, 16°45.130'N, 62°12.487'W, 27 June 2017, J.B. Runyon (MTEC, USNM).

###### Distribution.

Widely distributed in the American tropics (Dominica, Ecuador, Montserrat, Panama, Peru, and Venezuela).

###### Remarks.

See Naglis (2003) for a revision of the genus *Viridigona* and re-description of *V.thoracica*.

### Subfamily Medeterinae

#### Genus *Cryptopygiella* Robinson

##### Cryptopygiella musaphila

Taxon classificationAnimaliaDipteraDolichopodidae

Robinson

6C8702CF-4835-5864-B3A1-12F578B191A7

Fig. 6

Cryptopygiella musaphila Robinson, 1975: 41.

###### Material examined.

**Dominica: *Holotype*** ♂, La Ronde River, 15 February 1964, H. Robinson (USNM). **Montserrat**: 1 ♀, Bottomless Ghaut, 5 August 2005, yellow pan trap, V.G. Martinson; 1 ♀, Big River, 450 m, 16°45.690'N, 62°11.174'W, 28 June 2017, J.B. Runyon; 1 ♂, small ghaut, on *Heliconia*, 16°45.844'N, 62°11.402'W, 28 June 2017, J.B. Runyon (MTEC, USNM).

###### Distribution.

Dominica, Montserrat.

###### Remarks.

**Figure 6.**
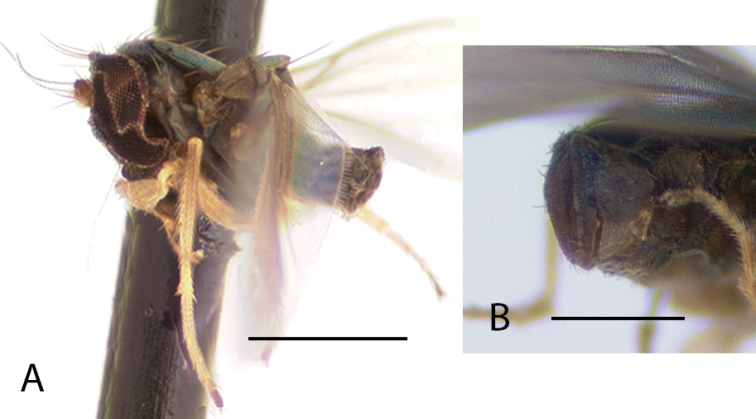
*Cryptopygiellamusaphila* Robinson, male **A** habitus, left lateral **B** tip of abdomen showing opening that encloses postabdomen, right lateral. Scale bars: 0.5 mm (**A**), 0.25 mm (**B**).

This is the first report of the monotypic genus *Cryptopygiella* outside of Dominica. Specimens seem to be restricted to the deepest ghauts on Montserrat. The male antenna and genitalia were illustrated by Bickel (2009). The male postabdomen is unique in being completely enclosed within an opening at apex of the preabdomen (Fig. 6B). Adults collected in 2017 were found on large *Heliconia* leaves, a similar habitat to where they were found on Dominica “running over the surface of banana leaves” (Robinson 1975).

#### Genus *Medetera* Fischer von Waldheim

##### Key to the species of *Medetera* in Montserrat

**Table:** 

1	Body length ca. 3.0 mm; thorax with 3 pairs of large dorsocentral setae and 4 large scutellar setae; femur III with 2–3 large setae on anterior surface; crossvein dm-cu slightly longer than last part of CuA_1_; male tarsus I with some segments flattened (Fig. 11)	***M.montserratensis* sp. nov.**
–	Body length ca. 2.2 mm or less; thorax with 2 pairs of large dorsocentral setae and 2 large scutellar setae (lateral pair small of lacking); femur III without large setae on anterior surface that are distinct from background setal field; crossvein dm-cu shorter than last part of CuA_1_; male tarsus I plain	**2**
2	Antenna with scape and pedicel yellow; femora yellow	***M.dominicensis* Robinson**
–	Antenna dark brown to black; femora mostly to wholly dark brown	**3**
3	Hypopygium of male not extending posteriorly beyond preabdomen; crossvein dm-cu ca. half as long as last part of CuA_1_; calypter with brown setae; body length ca. 1.5 mm	***M.pseudonigripes* Robinson**
–	Hypopygium of male extending posteriorly beyond preabdomen (most obvious in right lateral view); crossvein dm-cu two-thirds as long as last part of CuA_1_; calypter with yellow setae; body length ca. 2.0 mm	**4**
4	Face metallic dark blue, with little to no pruinosity (Fig. 8B); male cercus approximately as long as wide, square apically; male surstylus without thin translucent ventral lobe	***M.crassicauda* Robinson**
–	Face covered with dense golden-brown pruinosity (Fig. 8A); male cercus 2.5 × as long as wide, pointed apically; male surstylus with wide, thin translucent ventral lobe (Fig. 9)	***M.iviei* sp. nov.**

##### Medetera crassicauda

Taxon classificationAnimaliaDipteraDolichopodidae

Robinson

6DF2A08A-9F65-5B08-B46D-99096AE1D578

Medetera crassicauda Robinson, 1975: 27.

###### Material examined.

**Dominica: *Holotype*** ♂, South Chiltern, 26 March 1964, H. Robinson (USNM). **Montserrat**: 2 ♂, Cassava Ghaut, Beattie House, 8–17 April 2002, Malaise trap, A. Krakower (MTEC, USNM).

###### Distribution.

Dominica, Montserrat, Puerto Rico.

##### Medetera dominicensis

Taxon classificationAnimaliaDipteraDolichopodidae

Robinson

13CDEC00-DFD1-528B-85E5-07D349908B27

Medetera dominicensis Robinson, 1975: 26.

###### Material examined.

**Dominica: *Holotype*** ♂, Springfield Estate, 9 March 1964, H. Robinson (USNM). **Montserrat**: 9 ♂, 4 ♀, Cassava Ghaut, Beattie House, 14–21 January 2002, Malaise trap, A. Krakower; 5 ♂, 3 ♀, same as previous, 21 January–05 February 2002; 3 ♂, same as previous 5–15 February 2002; 8 ♂, same as previous, 4–23 March 2002; 4 ♂, 5 ♀, same as previous, 23 March–8 April 2002; 8 ♂, 1 ♀, same as previous, 18 March–4 April 2002; 12 ♂, 1 ♀, same as previous, 8–17 April 2002; 5 ♂, 1 ♀, same as previous, 17 April–1 May 2002; 4 ♂, same as previous, 17–30 May 2002; 4 ♂, 9 ♀, same as previous, 14–30 June 2002, M.A. Ivie; 1 ♂, same as previous, 30 June–4 July 2002; 5 ♂, Cassava Ghaut, 16°45.749'N, 62°12.473'W, canopy fogging at dawn, 5 December 2002, J. Boatswain & L. Martin; 1 ♂, same as previous, 4 February 2003, L. Martin & J. Boatswain; 19 ♂, 7 ♀, Underwood Ghaut, canopy fogging at dawn, 23 May 2002, K. Marske & J. Boatswain; 3 ♂, 2 ♀, Gun Hill, 18–30 May 2002, Malaise trap, K.A. Marske; 4 ♂, 1 ♀, same as previous, 2–19 June 2002; 2 ♂, 1 ♀, Woodlands, Riverside House, 5–7 January 2002, Malaise trap, Ivie, Marske & Puliafico; 1 ♂, same as previous, Malaise trap in lawn, 17–28 July 2005; 1 ♂, 2 ♀, Fogarty, 21 June 2002, canopy fogging at dawn, K.A. Marske & Forestry staff; 1 ♂, same as previous, 6 December 2002, J. Daley & L. Aymer; 1 ♂, 2 ♀, rental house in Old Town, 16°44.795'N, 62°13.711'W, 19 June 2017, J.B. Runyon; 1 ♂, 1 ♀, Hope Ghaut, 280 m, 16°45.101'N, 62°12.760'W, 20 June 2017, J.B. Runyon; 3 ♂, 1 ♀, Hope Ghaut, 300 m, 16°45.108'N, 62°12.695'W, 20 June 2017, J.B. Runyon; 5 ♂, Fogarty Ghaut, 16°46.41'N, 62°12.44'W, 21 June 2017, J.B. Runyon; 1 ♂, Woodlands Beach, 16°45.75'N, 62°13.42'W, 22 June 2017, J.B. Runyon; 2 ♂, 1 ♀, Runaway Ghaut, 175 m, 16°45.43'N, 62°12.89'W, 23 June 2017, J.B. Runyon; 2 ♂, Jack Boy Hill (top), 480 m, 16°45.797'N, 62°10.886'W, 25 June 2017, J.B. Runyon; 2 ♂, Fairy Walk River, 260 m, 16°45.162'N, 62°10.854'W, 26 June 2017, J.B. Runyon; 1 ♂, 2 ♀, ghaut above Montserrat Volcano Observatory, 330 m, 16°45.130'N, 62°12.487'W, 27 June 2017, J.B. Runyon (MTEC, USNM).

###### Distribution.

Dominica, Montserrat.

###### Remarks.

Adults of *M.dominicensis* were common in fogging samples and Malaise traps, and in 2017 were commonly seen on and collected from trunks of a wide variety tree species. Numerous males taken by fogging and in Malaise traps, presumably teneral, have the hypopygium yellowish rather than brown. Bickel (1985) indicated that *M.dominicensis* is closely related and could be conspecific with *M.nova* Van Duzee from eastern North America. Comparison of male specimens of *M.nova* (Pennsylvania, Virginia) with specimens of *M.dominicensis* reveals substantial and consistent differences in the form of the cercus and surstylus, notably the presence of branched hairs on the surstylus of *M.dominicensis*.

##### Medetera iviei

Taxon classificationAnimaliaDipteraDolichopodidae

sp. nov .

74AD2328-FBBA-5562-AE52-527A5955BDB4

http://zoobank.org/BB00592E-B1DA-4183-9313-1977BDAE94EC

Figs 7 , 8 , 9

###### Type material.

***Holotype***, ♂ labelled: “MONTSERRAT: Woodlands/ Riverside House, 140 ft/ 16°45.985'N, 62°13.341'W/ 10–12JAN2002, Malaise/ Ivie, Marske, Puliafico” “HOLOTYPE/ ♂ *Medetera*/ *iviei*/ Runyon [red label]” (USNM, type number USNMENT01350608).

###### Description.

**Figure 7.**
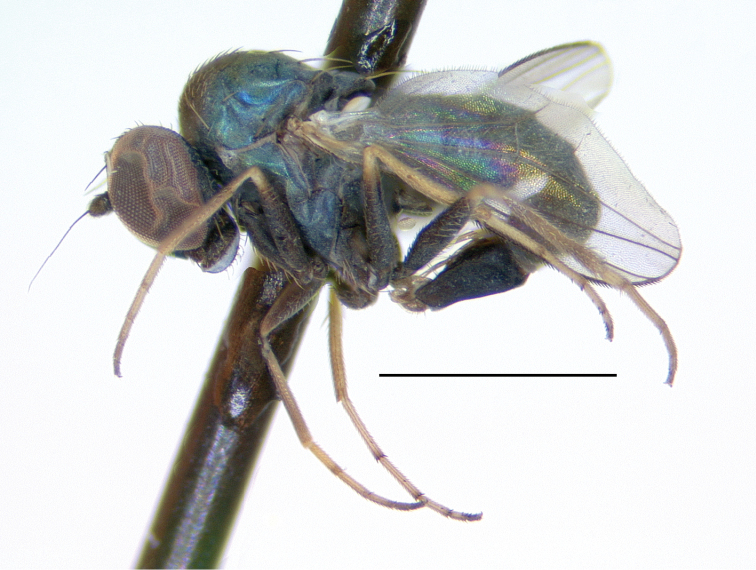
*Medeteraiviei* sp. nov. habitus of male holotype, left lateral. Scale bar: 1.0 mm.

**Figure 8.**
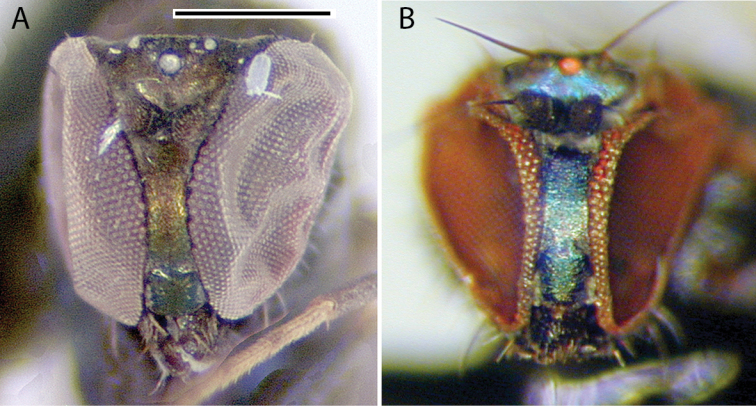
Heads of males, showing color differences of face and frons, anterior views **A***Medeteraiviei* sp. nov. **B***M.crassicauda* Robinson (Dominica, holotype). Scale bar: 0.5 mm.

**Figure 9.**
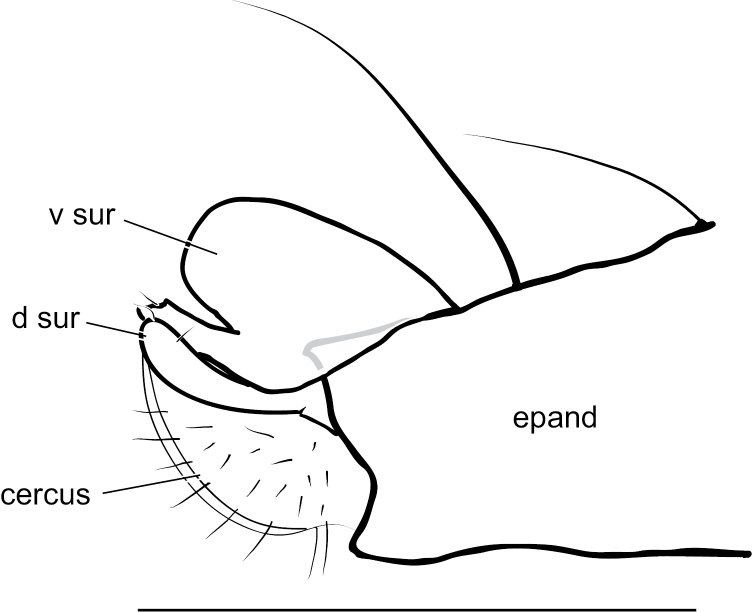
*Medeteraiviei* sp. nov. apex of male hypopygium, left lateral. Abbreviations: epand-epandrium; d sur-dorsal lobe of surstylus; v sur-ventral lobe of surstylus. Scale bar: 0.25 mm.

**Male** (Fig. 7). Body length 2.2 mm, wing length 1.8 × width 0.6 mm. ***Head***: Face (Fig. 8A) slightly narrowed near middle, covered with dense golden-brown pruinosity; clypeus with less dense golden-brown pruinosity revealing dark metallic blue-green cuticle. Frons with dense golden-brown pruinosity. Palpus ovate, shining black with a few small pale setae. Proboscis shining black. Antenna black; first flagellomere small, oval; arista-like stylus inserted just dorsal of apex. Lower postocular setae pale yellow to white. ***Thorax***: Scutum dorsally dark metallic green with brown pruinosity that is densest between acrostichal and dorsocentral rows of setae creating two indistinct brown stripes, scutum laterally metallic blue-green with gray pruinosity; setae on thorax light yellow; 8–10 pairs of small biseriate acrostichal setae; only posterior two pairs of dorsocentral setae noticeably enlarged; scutellum with one pair of large marginal setae and one pair of very small lateral setae. Pleuron dark metallic bluish green with gray pruinosity; with three small whitish setae on lower proepisternum. ***Legs***: Setae and setulae pale yellow to white except setae on coxa I and lateral setae on coxae II and III light brown. Coxae wholly dark brown. Femora dark brown with tips yellow, legs otherwise yellow. Femora and tibiae I and III without distinct setae. Tibia II with paired dorsal setae near 1/3 (ad seta slightly larger than pd seta), a ventral seta at apex; tibia III with two small black hooked pd spurs at apex subtended by small yellow concavity. Tarsi plain, tarsus II with some black spicules ventrally. Ratios of tibia:tarsomeres: leg I: 24–8–8–6–4–3; leg II: 26–14–12–7–3–2; leg III: 34–7–16–9–5–4. ***Wing***: Hyaline with brown veins, narrowly oval. R_2+3_ arching slightly backward; R_4+5_ curving more strongly backward than R_2+3_, approaching and becoming parallel with M near wing tip; crossvein dm-cu ca. two-thirds as long as last part of CuA_1_. Calypter white with white setae. Halter stem yellow-brown, knob white. ***Abdomen***: Dark brown with dark metallic green-blue reflections and slight gray pruinosity, with small yellowish setae. Hypopygium (Figs 7, 9) very large (slightly smaller than remainder of abdomen in right lateral view), fusiform, dark brown to almost black, projecting posteriorly beyond attachment to preabdomen and on a short peduncle formed by tergite VI. Hypopygial foramen left lateral near base. Epandrium dark brown to black, blunt apically, ventrally with large yellow erect seta basal to surstylus and smaller yellow seta basal to larger seta. Ventral lobe of surstylus bilobed, light brown, overlapping corner of epandrium at base, basoventral lobe large, thin, translucent, broadly rounded, and apicodorsal lobe smaller with a couple small lobes at tip and one or two small yellow apical hairs. Dorsal lobe of surstylus yellow, spatulate, with a couple small yellow hairs. Cercus brown, evenly rounded dorsally, pointed apically, with thin translucent membrane along dorsal edge, sparsely covered with short pale setae.

**Female.** Unknown.

###### Etymology.

This species is named for the coleopterist Michael A. Ivie (Montana State University) who led the invertebrate component of the Centre Hills Biodiversity Assessment project (2000–2005) and made this material available for study.

###### Distribution.

Montserrat.

###### Remarks.

*Medeteraiviei* is most similar to *M.crassicauda* Robinson to which it keys in Robinson (1975), most notably in males of both possessing an extremely large hypopygial capsule that extends backwards beyond the end of the preabdomen. Males of *M.iviei* differ from those of *M.crassicauda* in the color of the face (Fig. 8) and in the form of the cercus (in *M.iviei* the cercus is pointed apically with length 2.5 × width, in *M.crassicauda* the cercus is blunt apically and nearly square) and the surstylus (in *M.iviei* the surstylus has a wide, thin translucent ventral lobe that is absent in *M.crassicauda*).

##### Medetera montserratensis sp. nov.

Taxon classificationAnimaliaDipteraDolichopodidae

B8208346-F9D4-5A17-955E-F3EA3488C7A4

http://zoobank.org/46F07ADF-90AB-46F4-859C-F4A9D1CC6CD2

**Figure 10.**
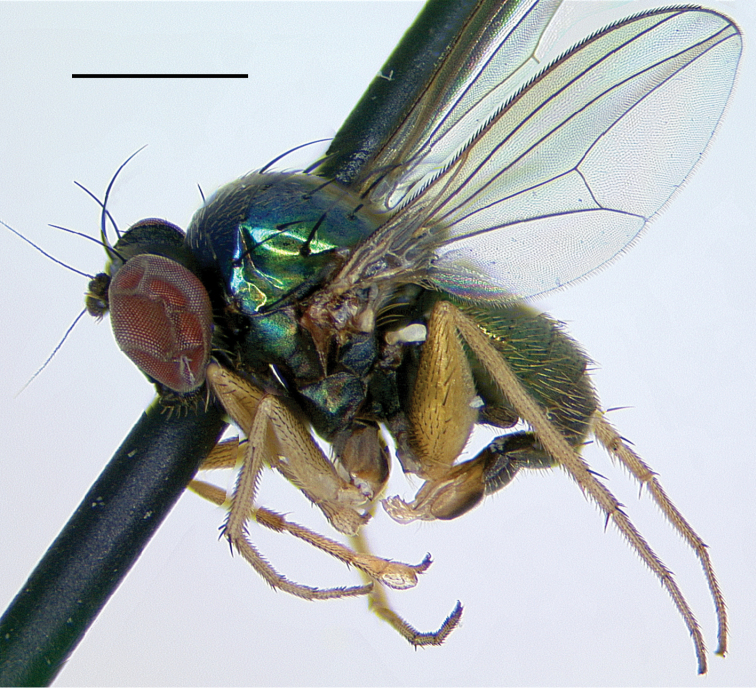
*Medeteramontserratensis* sp. nov. habitus of male holotype, left lateral. Scale bar: 1.0 mm.

**Figure 11.**
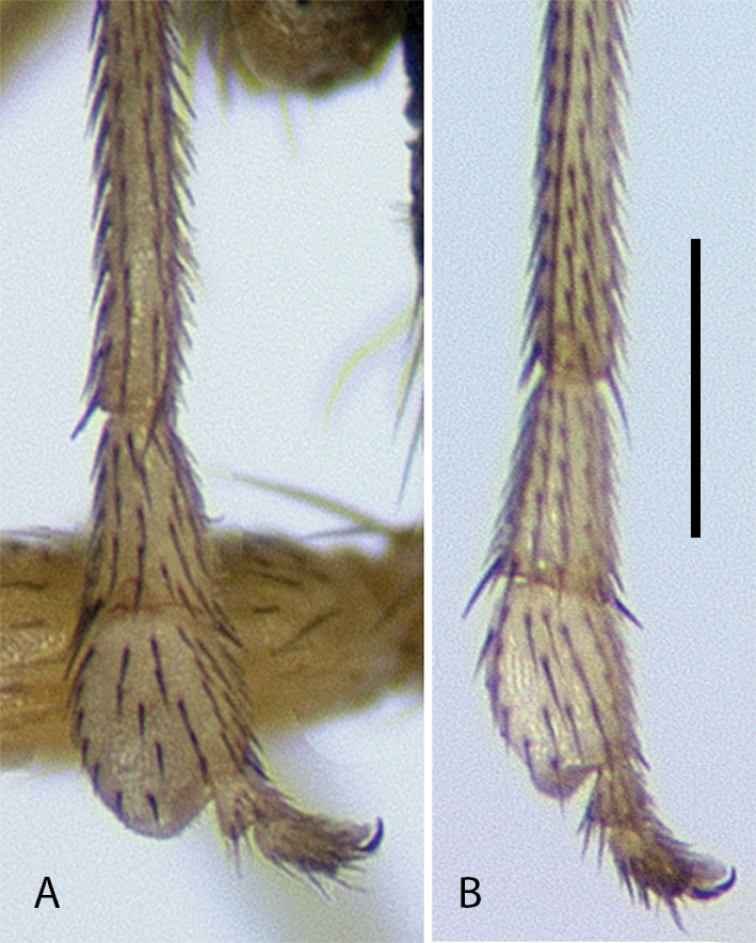
Tarsus I of males, posterior view **A***Medeteramontserratensis* sp. nov. **B***Medeterasteyskali* Robinson (Dominica). Scale bar: 0.25 mm.

**Figure 12.**
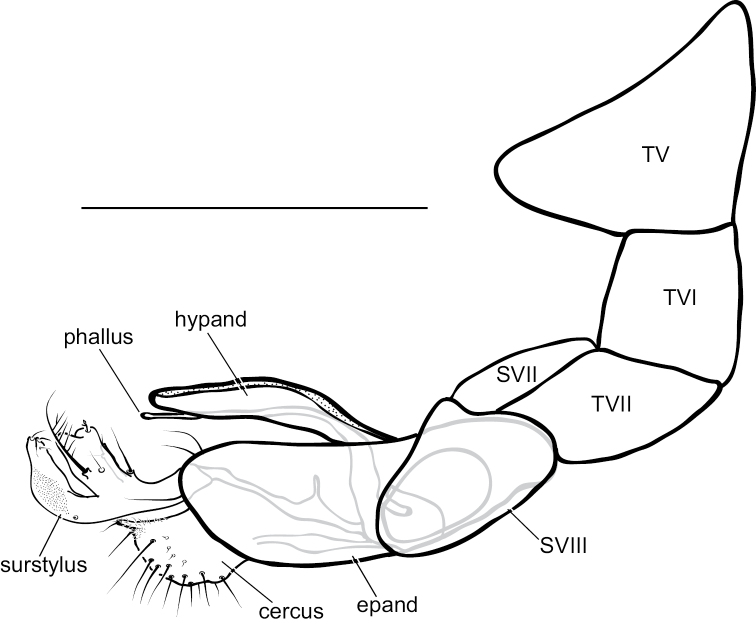
*Medeteramontserratensis* sp. nov. male terminalia, left lateral view. Abbreviations: epand-epandrium; hypand-hypandrium; T-tergite; S-sernite. Scale bar: 0.5 mm.

**Figure 13.**
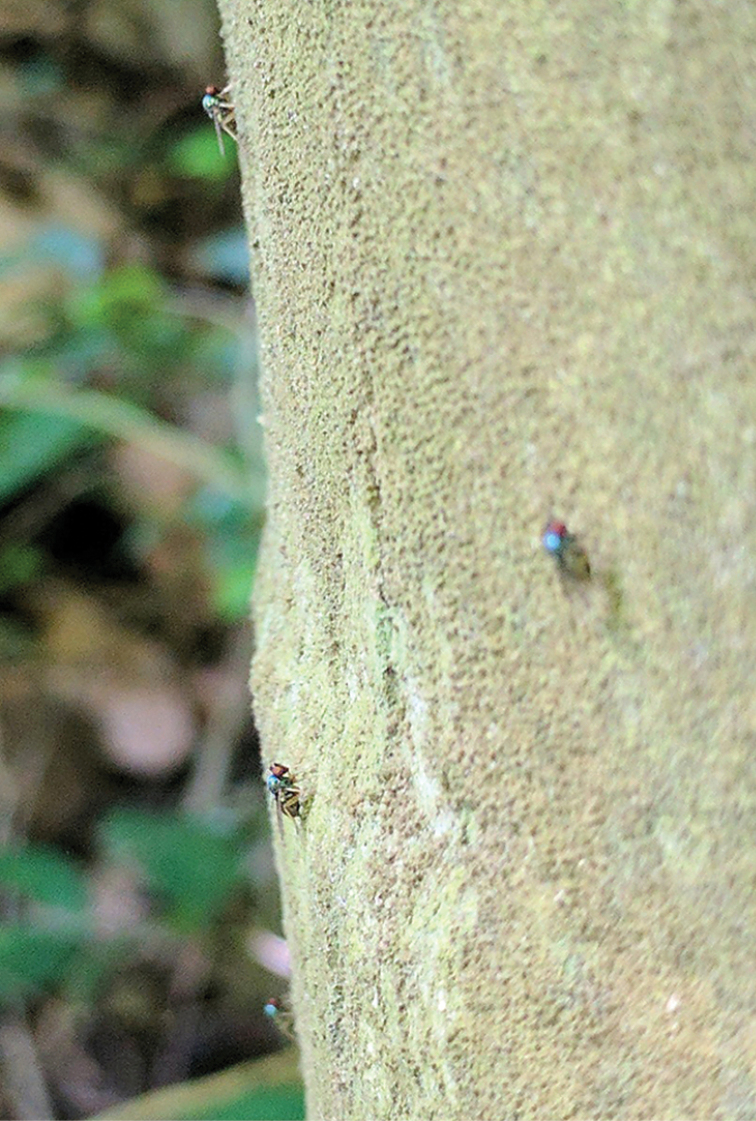
*Medeteramontserratensis* sp. nov., group of four males on palm trunk in Runaway Ghaut, Montserrat, 23 June 2017. Photograph by Justin Runyon.

Figs 10 , 11 , 12 , 13

###### Type material.

***Holotype***, ♂ labelled: “WEST INDIES: MONTSERRAT/ Fogarty Ghaut (Soldiers)/ 16°46.41'N, 62°12.44'W/ 21 June 2017, JB Runyon”; “HOLOTYPE/ ♂ *Medetera*/ *montserratensis*/ Runyon [red label]” (USNM, type number USNMENT01350609). ***Paratypes*: Montserrat**: 1 ♂, 1 ♀, same data as holotype; 10 ♂, Runaway Ghaut, 175 m, 16°45.43'N, 62°12.89'W, 23 June 2017, J.B. Runyon; 1 ♀, Jack Boy Hill (top), 480 m, 16°45.797'N, 62°10.886'W, 25 June 2017, J.B. Runyon (MTEC, USNM).

###### Description.

**Male** (Fig. 10). Body length 2.7–3.0 mm, wing length 2.5–2.8 × width 1.0–1.1 mm. ***Head***: Face and frons metallic dark blue-violet to green with little to no pruinosity, clypeus with brown pruinosity. Palpus black, rounded, covered with short brown setae and one larger brown to black seta near apex. Proboscis black with relatively large yellowish setae along margin. Antenna black, scape and pedicel sometimes somewhat yellow-orange ventrally; first flagellomere short, blunt, somewhat compressed laterally; arista-like stylus apical, inserted in slight sinus. Lower postocular setae white to yellow-white. ***Thorax***: Scutum dark metallic green with distinct violet reflections and slight grayish pruinosity, flattened area of posterior mesonotum reddish-copper; small setae yellow, large setae black; ca. 12 pairs of small yellow biseriate acrostichal setae that are not very distinct from numerous small setae covering anterior half of scutum, posterior-most pair of acrostichal setae diverging slightly; three pairs of large black dorsocentral setae; scutellum with two pairs of large black marginal setae, outer pair 3/4 as long as inner pair. Pleuron dark metallic bluish green with more grayish pruinosity than scutum; with 2–3 small yellow-brown to black setae on lower proepisternum. ***Legs***: Coxae dark brown with extreme tips becoming yellowish, coxa II often somewhat yellowish laterally; with yellow-brown setae. Legs otherwise yellow. Femora I and II with row of longer yellow-brown pv setae apically; femur II with short yellow ventral setae on basal half; femur III with setae on dorsal half brown, those on ventral half yellow, 2–3 distinct erect yellow-brown setae along middle of anterior surface and many longer erect brown ad setae at base. Tibia I without distinct setae, dorsal setulae brown, ventral setulae yellow; tibia II with setulae yellow to brown, with paired black dorsal setae near 1/3, five small brown apical setae; tibia III with setulae and most larger setae usually yellow, rather small brown ad seta near 1/5 and at apex, 4–5 large usually yellow pd setae. Tarsus I (Fig. 11A) with tarsomere I(2) flattened and broadened apically; tarsomere I(3) slightly longer than wide, rounded apically, concave on anterior surface; tarsomere I(4, 5) very small, combined length less than length of tarsomere I(3); tarsomere III(1) with minute black posterior spicule at base and several distinct small black apical setae. Ratios of tibia:tarsomeres: leg I: 36–20–10–11–2–4; leg II: 48–22–9–8–4–4; leg III: 64–12–24–16–6–5. ***Wing***: Hyaline, oval, with brown veins. Vein R_2+3_ nearly straight; R_4+5_ curving slightly backwards on apical half of wing, approaching and nearly parallel near apex with M_1_ which curves distinctly forward beyond crossvein dm-cu; crossvein dm-cu slightly longer than last part of CuA_1_. Calypter white with yellow-white setae. Halter knob and stem white. ***Abdomen***: Stout basally, rather abruptly tapered distally, with yellow setae, metallic green with copper reflections, obscured by little or no pruinosity. Sternites brown with many short pale yellow setae. Hypopygium (Fig. 12) on a peduncle created by segments VI and VII; tergite VI setose; segment VII dark brown with tergite setose, sternite bare and rather flattened ventrally and strongly sclerotized. Hypopygial foramen left basolateral. Sternite VIII relatively large, dark brown, forming a setose cap-like cover over hypopygial foramen. Epandrium elongate oval, 3 × as long as wide, brown dorsally and at base, becoming yellow ventrally, with two small ventral setae near apex. Surstylus yellow, with two large lobes; dorsal lobe with patch of sensilla and three or four small setae at apex; ventral lobe shallowly cordate apically, with large branched seta near 1/2 and finger-like medial lobe near base. Cercus yellow with yellow setae and hairs, rounded dorsally, rather flat ventrally, with small finger-like apical lobe. Phallus simple, narrow with round apex, slightly longer than hypandrium. Hypandrium arising near mid-length of epandrium, and forming hood over phallus, more sclerotized along ventral margin.

**Female.** Body length 3.1–3.2 mm, wing length 2.7–2.8 × width 1.1–1.2 mm. Similar to male, but tarsus I plain with ratios of tarsomeres: 18-10-7-3-4.

###### Etymology.

This species is named for the island of Montserrat.

###### Distribution.

Montserrat.

###### Remarks.

*Medeteramontserratensis* belongs to the *M.aberrans* species group that is characterized in part by having tarsomeres I (2, 3) flattened and modified (for other group characters see Bickel and Arnaud 2011). The *aberrans* group contains 27 species in the Neotropics (Yang et al. 2006, as *Saccopheronta* Becker), but only *M.montserratensis*, *M.steyskali* Robinson (Dominica), *M.excavata* (Becker) (Bolivia, Peru), and *M.metallina* (Becker) (Peru) have wholly yellow femora (the latter two species have “red-yellow” legs in contrast to the bright yellow legs of *M.montserratensis* and *M.steyskali*). *Medeteramontserratensis* and *M.steyskali* both are distinct from *M.excavata* which has an excavated tarsus I(3) (Becker 1922: fig. 55) and *M.metallina* which has black postorbital setae. *Medeteramontserratensis* is very similar to *M.steyskali*, and the two are likely sister taxa, but differs in having tarsomeres I (2, 3) broader (Fig. 11) and in small details in the shape of surstylar lobes (e.g., shape of the finger-like medial lobe).

Adults of *Medeteramontserratensis* were found on trunks of large palm trees, several times seen occurring in small aggregations of 4–6 individuals that were mostly males (Fig. 13).

##### Medetera pseudonigripes

Taxon classificationAnimaliaDipteraDolichopodidae

Robinson

261C1D29-1847-5DF6-8B74-37F2FD4CD40E

Medetera pseudonigripes Robinson, 1975: 28.

###### Material examined.

**Dominica: *Holotype*** ♂, Clarke Hall, 7 March 1964, H. Robinson (USNM). **Montserrat**: 2 ♂, Cassava Ghaut, Beattie House, 21–30 June 2002, uv light, M.A. Ivie; 1 ♂, same as previous, 6–30 June 2002, A. Krakower; 1 ♂, rental house in Old Town, 16°44.795'N, 62°13.711'W, 19 June 2017, J.B. Runyon. **St. Lucia**: Micoud District, Mamiku Gardens, 10–15 m, 13°52.11'N, 60°54.07'W, 9 May 2009, J.B. Runyon; 5 ♂, 1 ♀, nr. Micoud, trail to Fond Bay, 15 m, 13°49'48"N, 60°53'42"W, 16–22 May 2009, Malaise, S.D. Gaimari & A.R. Cline; 1 ♂, Barre de L’Isle trail, 285 m, 13.93268N, 60.95775W, 18–30 May 2009, Malaise, R.C. Winton & L.L. Ivie (MTEC, USNM).

###### Distribution.

Dominica, Montserrat, St. Lucia.

###### Remarks.

The tibiae in specimens from Montserrat are mostly yellow whereas those from Dominica and St. Lucia are mostly brown, but otherwise appear conspecific. The drawing of male genitalia of *M.pseudonigripes* in Robinson (1975: fig. 25) shows the surstylus pointed, but it is actually narrowly spatulate with tip shallowly concave or notched. This species belongs to the *M.isobellae* species group most notably in having the surstyli fused into expanded apical lobe with modified setae medially (Bickel 1985). *M.pseudonigripes* is very similar to the Nearctic *M.isobellae* Bickel, but the latter species has the surstylus much broader apically with the tip not concave or notched.

#### Genus *Systenus* Loew

##### Systenus ladonnae

Taxon classificationAnimaliaDipteraDolichopodidae

sp. nov .

85D74600-8C42-58D6-9DEC-52CE50A84A95

http://zoobank.org/20575311-14CB-4EC6-A3E8-7F1BE369715F

Figs 14 , 15

###### Type material.

***Holotype***, ♂ labelled: “MONTSERRAT: Cassava/ Ghaut, Beattie House/ 16°45.91'N, 62°12.95W/ 04–23MAR2002, 632 ft./ A. Krakower, Malaise” “HOLOTYPE/ ♂ *Systenus*/ *ladonnae*/ Runyon [red label]” (USNM, type number USNMENT01350610). ***Paratypes***: **Dominica**: 1 ♀, Cabrits National Park, East Cabrits Trail, 15.58564N, 61.47210W, Malaise, 30 May–7 June 2011, M.A. & L.L. Ivie. **Montserrat**: 1 ♂, same data as holotype, 23 March–3 April 2002, uv light, K.A. Marske; 1 ♂, Hill above Hope Ghaut, 16°45.17'N, 62°12.74'W, canopy fogging at dawn, 4 December 2002, 1,051 ft, J. Boatswain & J. Martin. **Nevis**: 1 ♂, Lover’s Beach, 17.20451N, 62.60577W, 21 March 2017, Malaise, W. Smithen; 1 ♀, same as previous, 26 April 2017. **St. Kitts**: 1 ♀, Dos d’Ane Pond Trail, 17°20.049'N, 62°48.012'W, Malaise, 31 July–12 August 2017 (MTEC, USNM).

**Figure 14.**
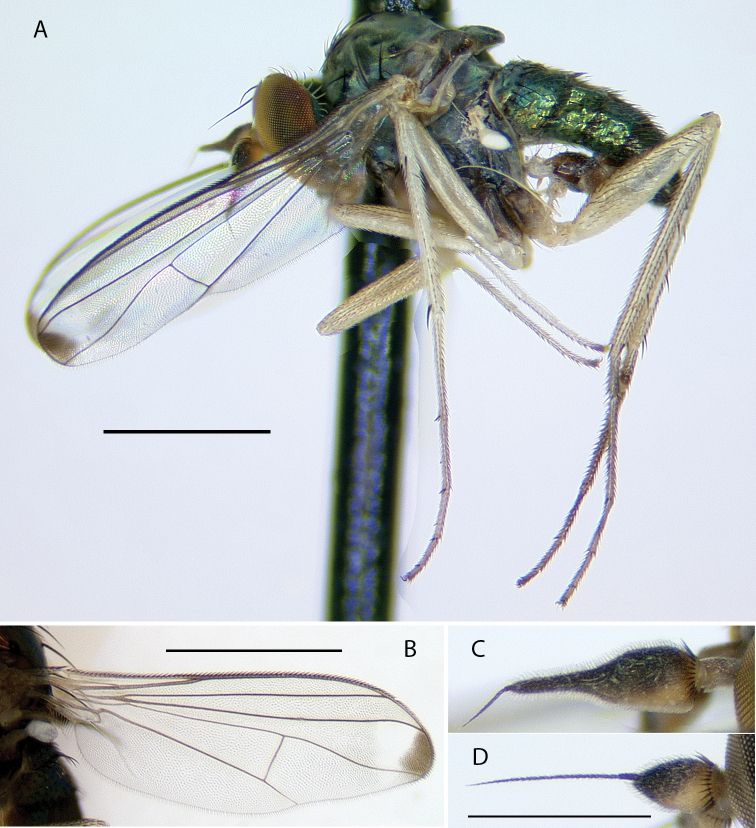
*Systenusladonnae* sp. nov. **A** habitus of male holotype, left lateral **B** male wing, dorsal **C** male antenna, left lateral **D** female antenna, left lateral. Scale bars: 1.0 mm (**A, B**), 0.5 mm (**C, D**).

**Figure 15.**
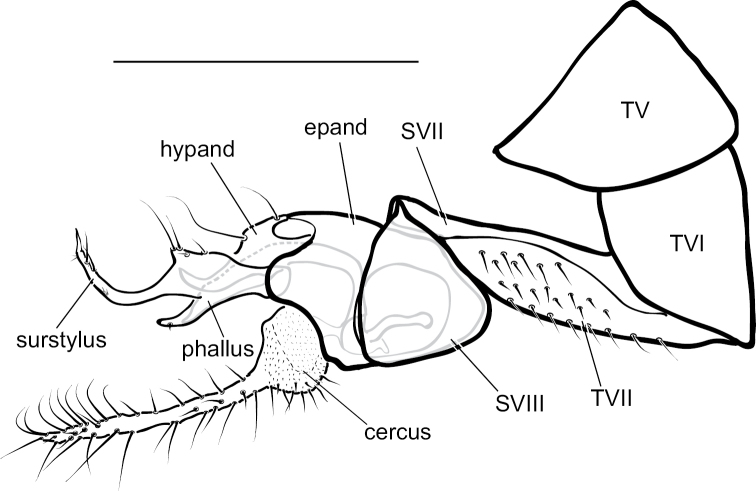
*Systenusladonnae* sp. nov. male terminalia, left lateral. Abbreviations: epand-epandrium; hypand-hypandrium; T-tergite; S-sernite. Scale bar: 0.5 mm.

**Description. Male** (Fig. 14A–C). Body length 2.9 mm, wing length 2.6 × width 0.9 mm. ***Head***: Face narrowed below but eyes distinctly separated (ca. five ommatidia wide at narrowest point); face and frons dark metallic green with some violet reflections, covered with thick grayish pruinosity. Palpus yellow with short black setae and one larger seta near apex. Proboscis yellow, keel-like, projecting anteriorly. Antenna (Fig. 14C) with scape and pedicel yellowish, scape without dorsal setae; first flagellomere yellow on approximately basal third below, otherwise brown, subrectangular basally and abruptly narrowed to elongate tapering point in distal half, covered with short thick pubescence; arista-like stylus apical, short, length subequal to basal width of first flagellomere. Postcranium dorsally concave. Postocular setae in a single row, wholly white. ***Thorax***: Scutum dark metallic green with a bronze stripe between acrostichal setae, covered with rather dense light gray pruinosity; posterior mesonotum distinctly flattened; ca. 12 pairs of biseriate acrostichal setae; six strong dorsocentral setae; scutellum with two pairs of large marginal setae, lateral pair smaller. Pleuron wholly green with dense light grey pruinosity; proepisternum with one strong ventrally projecting white seta above base of coxa I. ***Legs***: Coxa I yellow with approximately basal half brown, with yellow setae on anterior surface, those near apex large; coxa II dark brown with yellow anterior setae, without lateral seta; coxa III dark brown with apex becoming yellow, with large yellow ad seta near 1/2. Remainder of legs yellow, except distal tarsomeres brownish. Femora lacking anterior preapical setae; femora II and III with ventral surface appearing fuzzy due to very short yellow microsetulae. Tibiae I and II covered with short ivory-colored vestiture; tibia I bare of major setae; tibia II with black dorsal and ad seta near 1/5, with two black apical setae anteriorly, remainder of apical setae very short; tibia III with 4–5 pd setae scattered along length. Tarsus III covered in short, stiff setulae which are longest ventrally (length subequal to width of tarsomeres). Ratios of tibia:tarsomeres: leg I: 18–8–5–3–2–2; leg II: 22–12–8–5–3–2; leg III: 26–5–12–6–4–3. ***Wing*** (Fig. 14B): Hyaline, but with apical brown maculation between R_4+5_ and M_1_ that is immediately preceded by a similar-sized white spot with white microtrichia; vein R_4+5_ nearly straight then bent posteriorly near apex; vein M_1_ bowed beyond crossvein dm-cu so that veins R_4+5_ and M_1_ diverge and then converge near apex. Calypter yellow with fan of yellow-brown setae. Halter pale yellow. ***Abdomen***: Metallic green with bronze reflections and dusting of gray pruinosity; posterior margin of tergite I with row of long brown to black setae, tergites otherwise covered with short brown to black setae. Sternites II–VI membranous or only weakly sclerotized, somewhat recessed; sternite VIII forming a setose cap-like cover over hypopygial foramen. Hypopygium (Fig. 15) on an elongate narrow peduncle formed by tergite and sternite VII that are separated by partially sclerotized pleural membrane, sternite VII glabrous, tergite VII setose. Hypopygial foramen left lateral near base. Epandrium dark brown, a little longer than wide. Surstylus yellow, antler-shaped, with rather large dorsal lobe before 1/2, small setae-bearing ventral lobe near 1/2, and long, slender finger-like apical lobe. Cercus yellow, with rounded swollen base, elongate and digitiform distally with many yellow setae. Phallus simple, rather broad throughout and narrowed near apex, arching dorsally on apical half. Hypandrium somewhat wishbone-shaped in ventral view, up curved distally in lateral view, attached to epandrium by a membrane.

**Female.** Body length 2.7–3.0 mm, wing length 2.5–2.6 × width 0.9–1.0 mm. Similar to male, but face wider (width slightly less than width of first flagellomere); antenna (Fig. 14D) shorter, ovoid with pointed apex; arista-like stylus longer than first flagellomere; wing without maculation, but some specimens with hint of brown clouding at and just behind apex of R_4+5_.

###### Etymology.

This species is named in honor of LaDonna Ivie (Bozeman, MT) whose hard work and expertise made the Montserrat biodiversity project possible. She ran many traps on Montserrat and the Malaise trap on Dominica that collected the only known specimen of this genus/species from that island.

###### Distribution.

Dominica, Montserrat, Nevis, St. Kitts.

###### Remarks.

This is the first report of the genus *Systenus* in the Lesser Antilles and is the 23^rd^ species described from the New World. *Systenusladonnae* is similar to *S.maculipennis* Bickel from Costa Rica (Bickel, 2015), but *S.maculipennis* differs most notably in the shape of the wing apex (wing membrane is reduced posteriad of distal vein M in *S.maculipennis*) and in lacking a white spot on wing. *Systenusladonnae* also resembles the Nearctic *S.apicalis* Wirth which also has a white and black spot near wing apex, but in *S.apicalis* the white spot is apical to the brown spot.

Specimens were collected in dry forests near the coast and low elevation mesic forests.

#### Genus *Thrypticus* Gerstäcker

##### Key to the species of *Thrypticus* in Montserrat

**Table:** 

1	Abdominal tergite I and often tergite II yellow	***T.abdominalis* (Say)**
–	Abdominal tergites wholly metallic green	**2**
2	Basal abdominal sternites yellow; male surstylus with 2 very long setae at apex	***T.violaceus* Van Duzee**
–	Abdominal sternites dark; male surstylus without 2 exceptionally long apical setae	**3**
3	Thorax with 8 or more pairs of acrostichal setae with hindmost offset laterally; male surstylus as long as epandrium	***T.parvulus* Van Duzee**
–	Thorax with 5 or 6 (rarely 7) pairs of acrostichal setae in nearly straight rows; hypopygial appendages shorter than epandrium	**4**
4	Antenna with scape and pedicel yellow; tarsomeres III(1, 2) of ca. equal length; epandrium tapered from base to appendages, rather pointed apically; hypopygial appendages yellow	***T.aequalis* Robinson**
–	Antenna wholly brown; tarsus III(1) distinctly shorter than tarsus III(2); epandrium scarcely tapered from base to tip, blunt apically (Fig. 17); hypopygium with median appendage (dorsal lobe of surstylus) brown (Fig. 16B)	***T.mediofuscus* sp. nov.**

##### Thrypticus abdominalis

Taxon classificationAnimaliaDipteraDolichopodidae

(Say)

587C0F07-3D1E-5F55-AD99-AE5A380D0ECD

Chrysotus abdominalis Say, 1829–1830: 169.
Xanthotricha cupulifer Aldrich, 1896: 339.

###### Material examined.

**Dominica**: 1 ♂, Grande Savane, 1 February 1965, W.W. Wirth (USNM); 1 ♂, Springfield Estate, yellow pan, 1–3 June 2011. **Montserrat**: 1 ♂, 1 ♀, Cassava Ghaut, Beattie House, 8–17 April 2002, Malaise trap, A. Krakower; 1 ♀, same as previous, 14–30 June 2002; 1 ♂, 2 ♀, Gun Hill, 2–19 June 2002, Malaise trap, K.A. Marske (MTEC, USNM).

###### Distribution.

*Thrypticusabdominalis* is a widespread species, occurring in central and eastern North America, Central America, and throughout the West Indies.

##### Thrypticus aequalis

Taxon classificationAnimaliaDipteraDolichopodidae

Robinson

BD211EB7-197C-5EFB-B0A2-3398E6A2955F

Thrypticus aequalis Robinson, 1975: 36.

###### Material examined.

**Dominica**: 2 ♀, Clarke Hall, Malaise trap, 8–10 January 1965, W.W. Wirth (USNM); 1 ♂, 2 ♀, Springfield Estate, yellow pan trap, 1–3 June 2011, M.A. & L.L. Ivie. **Montserrat**: 1 ♀, Bottomless Ghaut, 5 August 2005, yellow pan trap, V.G. Martinson (MTEC).

###### Distribution.

Dominica, Montserrat.

###### Remarks.

The female specimen from Montserrat matches female paratypes (the holotype is a male) and other specimens from Dominica notably by the distinctive structure of the ovipositor (Robinson 1975, fig. 59).

##### Thrypticus mediofuscus sp. nov.

Taxon classificationAnimaliaDipteraDolichopodidae

4AE82CA0-CBD9-500B-AF6D-4D87FAC2C1B6

http://zoobank.org/0E5CEF17-C104-4AC2-A845-D9DE6D680E06

Figs 16 , 17

###### Type material.

***Holotype***, ♂ labelled: “MONTSERRAT:/ Sweetwater Ghaut/ 01 Aug 2005/ Yellow Pan Trap/ V.G. Martinson”; “HOLOTYPE/ ♂ *Thrypticus*/ *mediofuscus*/ Runyon [red label]” (USNM, type number USNMENT01350611). ***Paratypes*: Dominica**: 3 ♂, Springfield Estate, 1–3 June 2011, yellow pan trap, M.A. & L.L. Ivie; 1 ♂, St. Paul Parish, Springfield Estate, 29 May 2011, FIT with yellow pan, M.A. & L.L. Ivie. **Montserrat**: 1 ♂, rental house in Old Town, 16°44.79'N, 62°13.711'W, 80 m, yellow pan traps, 19 June 2017, J.B. Runyon (MTEC, USNM).

**Figure 16.**
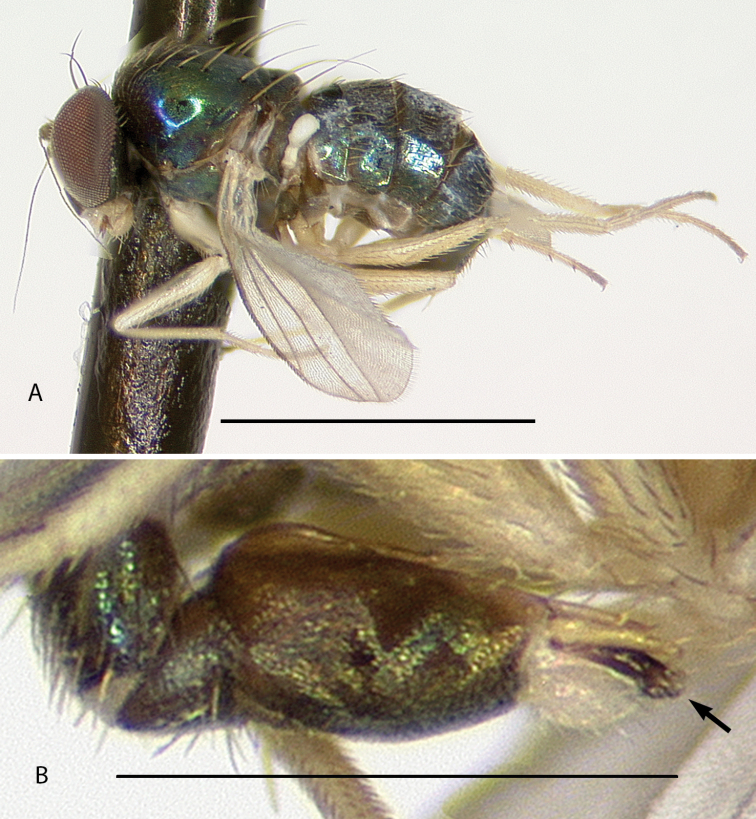
*Thrypticusmediofuscus* sp. nov. **A** habitus of holotype male, left lateral **B** male terminalia, right lateral, arrow indicates darker middle hypopygial appendage (dorsal lobe of surstylus). Scale bars: 1.0 mm (**A**), 0.5 mm (**B**).

**Figure 17.**
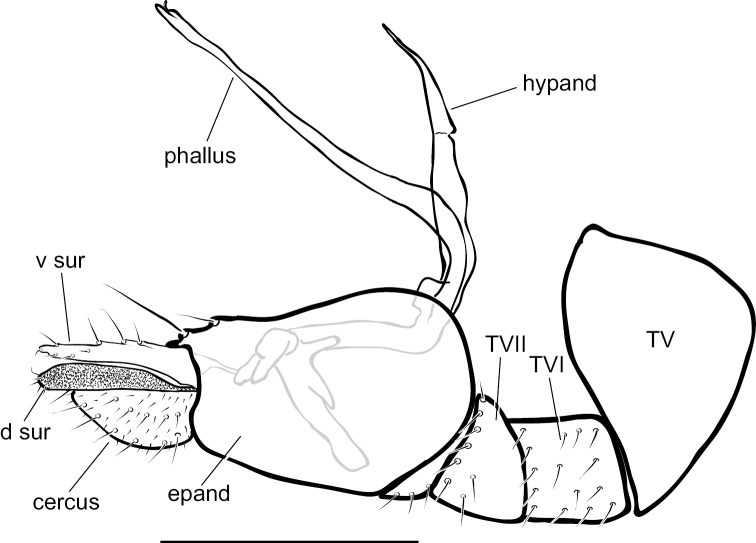
*Thrypticusmediofuscus* sp. nov. male terminalia, left lateral. Abbreviations: epand-epandrium; d sur-dorsal lobe of surstylus; hypand-hypandrium; T-tergite; v sur-ventral lobe of surstylus. Scale bar: 0.25 mm.

**Description. Male** (Fig. 16). Body length 1.7 mm, wing length 1.4 × width 0.5 mm. ***Head***: Face wider than first flagellomere, dark brown with little to no violet reflections and very sparse light brown pruinosity, lower face with denser light brown pruinosity. Frons dark brown with strong violet reflections. Palpus yellow, slender, ovate with larger subapical yellow seta. Proboscis yellow with yellow marginal hairs. Antenna brown, often slightly dark reddish; first flagellomere rounded, very short, only slightly larger than scape; arista-like stylus apical. Lower postocular setae pale yellow to white, ventral-most seta distinctly larger. ***Thorax***: Scutum dark metallic green with slight grayish pruinosity and strong violet reflections on anterior half; setae yellow to yellow-brown; ca. six pairs of small biseriate acrostichal setae, posterior-most acrostichal setae not distinctly diverging; five or six pairs of dorsocentral setae, posterior four pairs large, increasing in size posteriorly; scutellum with a pair of large marginal setae and minute seta on outer margin. Pleuron concolorous with scutum but more brownish above coxae II and III, without violet reflections; with a yellow seta on lower proepisternum. ***Legs***: Wholly yellow with yellow to yellow-brown setae. Femur and tibia I without distinctive setae. Tibia II with large distally directed ad seta at 1/3 and ventral seta at apex; tibia III with row of very short erect comb-like setae (length ca. half width of tibia) on apical half dorsally and similar smaller row of such setae ventrally. Tarsomere I(1) with ventral row of very short erect comb-like setae (length < width of tarsomere). Ratios of tibia:tarsomeres: leg I: 18–8–4–3–2–3; leg II: 24–10–6–4–3–2; leg III: 28–6–8–5–3–3. ***Wing***: Hyaline, oval, with light brown veins. Vein R_2+3_ essentially straight and diverging from R_4+5_ throughout, ending in costa near 4/5 of wing length; R_4+5_ curving slightly backwards on apical half of wing, approaching and subparallel to M_1_ near apex, half as far from M_1_ at apex than opposite crossvein dm-cu; M_1_ essentially straight beyond crossvein dm-cu, ending in wing apex; last part of CuA_1_ ca. 3.5 × longer than crossvein dm-cu. Calypter white with yellow-white setae. Halter knob and stem white. ***Abdomen***: Tergites metallic dark green with slight bluish reflections, obscured by little or no pruinosity, setae yellow. Sternites brown with short pale brown setae. Hypopygium (Figs 16B, 17) on a short peduncle created by segments VI and VII. Epandrium dark brown with some metallic green reflections and sparse grayish pruinosity and two very small ventral lobes near apex each bearing a seta; hypopygial foramen left basolateral. Surstylus and cercus not spreading, forming a compact structure that in ventral view is oval with pointed apex; ventral lobe of surstylus pale yellow, shining, with four rather erect yellow ventral setae spaced along length; dorsal lobe of surstylus shining brown, with small hairs at apex. Cercus pale yellow, evenly rounded dorsally and covered with yellow hairs and two rows of slightly larger yellow setae. Phallus bifurcate at apex, with subquadrate membranous ventral lobe near base. Hypandrium with flexion and indentation near 2/3, caudate and membranous apically.

**Female.** Unknown.

###### Etymology.

This species is named for the dark middle appendage of the hypopygium (dorsal lobe of surstylus) of the male in lateral view (Fig. 16B).

###### Distribution.

Dominica, Montserrat.

###### Remarks.

In Robinson (1975), *Thrypticusmediofuscus* keys to *T.delicatus* Robinson (holotype examined), but is distinguished by larger body size (1.2 mm in *T.delicatus*), having coxa II wholly yellow (brownish in *T.delicatus*), relative length of tarsomeres I(1, 2) as 2:1 (5:4 in *T.delicatus*) and having dorsal lobe of surstylus dark brown (Figs 16B, 17; hypopygial appendages wholly yellow in *T.delicatus*). *Thrypticusmediofuscus* is the only *Thrypticus* species in the Lesser Antilles with a bicolored surstylus, however, I have seen a related undescribed species from St. Lucia.

All specimens from both Montserrat and Dominica were collected in yellow pan traps.

##### Thrypticus parvulus

Taxon classificationAnimaliaDipteraDolichopodidae

Van Duzee

CC4C2CDA-5F93-52E9-8F00-8F9AA269BAE9

Thrypticus parvulus Van Duzee, 1930a: 86.

###### Material examined.

**Montserrat**: 1 ♂, 1 ♀, Cassava Ghaut, Beattie House, 14–30 June 2002, Malaise trap, M.A. Ivie; 1 ♀, same as previous, 10–12 January 2002, Ivie, Marske & Puliafico. 1 ♀, Fogarty, 20–22 June 2002, Malaise trap, K.A. Marske (MTEC).

###### Distribution.

Dominica, Montserrat, St. Vincent.

##### Thrypticus violaceus

Taxon classificationAnimaliaDipteraDolichopodidae

Van Duzee

BFB20CEA-9895-5417-9E59-747F45FB792F

Thrypticus violaceus Van Duzee, 1927: 5.
Thrypticus setosus Robinson, 1964: 118.

###### Material examined.

**Dominica**: 1 ♂, Fond Colet, 5–9 October 1964, P.J. Spangler (USNM) **Montserrat**: 12 ♂, 12 ♀, Woodlands, Riverside House, 10–12 January 2002, Malaise trap, Ivie, Marske & Puliafico; 9 ♂, 4 ♀, same as previous, 5–7 January 2002; 4 ♂, 2 ♀, same as previous, 17–28 July 2005, WIBF group; 2 ♂, 3 ♀, Hope Ghaut, 8–10 January 2002, yellow pan traps, K.A. Marske; 1 ♂, Cassava Ghaut, Beattie House, 14–30 June 2002, Malaise trap, M.A. Ivie; 1 ♀, Gun Hill, 2–19 June 2002, Malaise trap, K.A. Marske (MTEC, USNM); 1 ♀, March 1910 (USNM).

###### Distribution.

North America (Florida, North Carolina, and Texas) and the West Indies (Haiti, Puerto Rico, Dominica, and Montserrat).

### Subfamily Achalcinae

#### Genus *Xanthina* Aldrich

##### Xanthina rubromarginata

Taxon classificationAnimaliaDipteraDolichopodidae

Robinson

5DE21178-929A-5E97-B3A5-B7A90979E7D2

Xanthina rubromarginata Robinson, 1975: 44.

###### Material examined.

**Dominica**: 1 ♂, Trafalgar Falls, 15 March 1964, H. Robinson (USNM). **Montserrat**: 3 ♂, 3 ♀, Woodlands, Riverside House, 8–10 January 2002, yellow pan traps, K. Marske & K. Puliafico; 1 ♀, same as previous, 22 July 2005, V.G. Martinson; 1 ♀, Bottomless Ghaut to Big River trail, 14 August 2005, yellow pan traps, V.G. Martinson; 1 ♂, 4 ♀, Cassava Ghaut, 24 July 2005, yellow pan traps, V.G. Martinson; 1 ♂, 1 ♀, Big River, 5 August 2005, yellow pan traps, V.G. Martinson; 1 ♂, Hope Ghaut, 300 m, 16°45.108'N, 62°12.695'W, 20 June 2017, J.B. Runyon; 1 ♂, 2 ♀, Jack Boy Hill (top), 480 m, 16°45.797'N, 62°10.886'W, 25 June 2017, J.B. Runyon; 1 ♂, 2 ♀, ghaut above Montserrat Volcano Observatory, 330 m, 16°45.130'N, 62°12.487'W, 27 June 2017, J.B. Runyon; 1 ♂, Big River, 450 m, 16°45.690'N, 62°11.174'W, 28 June 2017, J.B. Runyon (MTEC, USNM).

###### Distribution.

Dominica, Montserrat.

###### Remarks.

Adults were collected in yellow pan traps and by sweeping moist, deeply shaded ground in mesic forests.

### Subfamily Enliniinae

#### Genus *Enlinia* Aldrich

##### Enlinia patellitarsis

Taxon classificationAnimaliaDipteraDolichopodidae

Robinson

29B7E488-49B3-5B44-B132-E5B855634805

Enlinia patellitarsis Robinson, 1975: 48.

###### Material examined.

**Dominica: *Holotype*** ♂, Freshwater Lake, 23 February 1964, H. Robinson (USNM). **Montserrat**: 8 ♂, 4 ♀, Runaway Ghaut, roadside springs, 150 m, 16°45.449'N, 62°13.011'W, 22 June 2017, J.B. Runyon; 14 ♂, 3 ♀, Hope Ghaut, 300 m, 16°45.108'N, 62°12.695'W, 20 June 2017, J.B. Runyon; 4 ♂, 1 ♀, same as previous, 280 m, 16°45.101'N, 62°12.760'W (MTEC, USNM).

###### Distribution.

Dominica, Montserrat.

###### Remarks.

Adults were found hovering closely to nearly vertical rocky surfaces of dripping springs and in a small creek on wet rock surfaces being occasionally splashed by water. Adults were found in similar habitats on Dominica (Robinson 1975: 49).

#### Genus *Harmstonia* Robinson

##### Harmstonia simplex

Taxon classificationAnimaliaDipteraDolichopodidae

Robinson

E4353855-B0A2-5AA8-A325-EBD68251486B

Harmstonia simplex Robinson, 1967a: 5.

###### Material examined.

**Dominica: *Holotype*** ♂, Clarke Hall, 11–20 February 1965, W.W. Wirth (USNM). **Montserrat**: 2 ♂, 1 ♀, Big River, 5 August 2005, yellow pan traps, V.G. Martinson; 1 ♀, Bottomless Ghaut, 5 August 2005, yellow pan traps, V.G. Martinson; 3 ♂, 3 ♀, Hope Ghaut, 300 m, 16°45.108'N, 62°12.695'W, 20 June 2017, J.B. Runyon; 5 ♂, 5 ♀, same as previous, 280 m, 16°45.101'N, 62°12.760'W; 5 ♀, Runaway Ghaut, roadside springs, 150 m, 16°45.449'N, 62°13.011'W, 22 June 2017, J.B. Runyon; 1 ♂, Runaway Ghaut, 175 m, 16°45.43'N, 62°12.89'W, 23 June 2017, J.B. Runyon; 1 ♂, 1 ♀, Corbett Spring, 300 m, 16°45.012'N, 62°11.184'W, 26 June 2017, J.B. Runyon; 2 ♂, 1 ♀, Fairy Walk River, 260 m, 16°45.162'N, 62°10.854'W, 26 June 2017, J.B. Runyon; 2 ♂, 2 ♀, ghaut above Montserrat Volcano Observatory, 330 m, 16°45.130'N, 62°12.487'W, 27 June 2017, J.B. Runyon; 2 ♂, 1 ♀, Bottomless Ghaut, 400 m, 16°45.994'N, 62°11.497'W, 28 June 2017, J.B. Runyon; 5 ♂, 5 ♀, Big River, 450 m, 16°45.690'N, 62°11.174'W, 28 June 2017, J.B. Runyon (MTEC, USNM).

###### Distribution.

Dominica, Montserrat.

###### Remarks.

This is the only species of *Harmstonia* known from the Lesser Antilles. Adults were found on moist rocks in ghauts, but unlike *Enliniapatellitarsis*, do not require running or splashing water.

### Subfamily Peloropeodinae

#### Genus *Micromorphus* Mik

##### Micromorphus albipes

Taxon classificationAnimaliaDipteraDolichopodidae

(Zetterstedt)

EA31C5A6-EE1F-516D-BE6E-48FB37BE9FAB

Hydrophorus albipes Zetterstedt, 1843: 454.
Achalcus caudatus Aldrich, 1902: 93.
Micromorphus panamensis Van Duzee, 1931a: 180.

###### Material examined.

**Dominica**: 2 ♂, 29 January 1964, H. Robinson (USNM); 1 ♂, St. Paul Parish, near Pont Casse (trail), Morne Trois Pitons, humid forest, Malaise, 750 m, 16–17 April 2004, M.E. Irwin & B.M. Shepard. **Montserrat**: 1 ♀, Bottomless Ghaut, 5 August 2005, yellow pan traps, V.G. Martinson; 2 ♀, Hope Ghaut, 300 m, 16°45.108'N, 62°12.695'W, 20 June 2017, J.B. Runyon; 1 ♂, 8 ♀, Jack Boy Hill (top), 480 m, 16°45.797'N, 62°10.886'W, 25 June 2017, J.B. Runyon; 1 ♂, 2 ♀, ghaut above Montserrat Volcano Observatory, 330 m, 16°45.130'N, 62°12.487'W, 27 June 2017, J.B. Runyon; 4 ♂, 2 ♀, Big River, 450 m, 16°45.690'N, 62°11.174'W, 28 June 2017, J.B. Runyon; 1 ♂, Katy Hill (top), 730 m, 16°45.731'N, 62°11.646'W, 28 June 2017, J.B. Runyon (MTEC, USNM).

###### Distribution.

*Micromorphusalbipes* is exceptionally widespread being reported from the Nearctic, Neotropics, Oriental, and Palearctic realms (Pollet et al. 2004).

###### Remarks.

Robinson (1975) identified this species in Dominica, and specimens from Montserrat match those from Dominica. However, as noted by Pollet et al. (2004), there is some question about whether Zetterstedt’s species is truly so ubiquitous and careful comparison of specimens from each realm is needed. In 2017, adults were obtained by sweeping moist, shaded ground in mesic forests.

#### Genus *Peloropeodes* Wheeler

##### Peloropeodes frater

Taxon classificationAnimaliaDipteraDolichopodidae

(Aldrich)

37C6E449-EDCB-5AAC-9698-7D0B30F1500C

Sympycnus frater Aldrich, 1902: 83.

###### Material examined.

**Dominica**: 1 ♂, St. John Parish, Cabrits National Park, East Cabrits Trail, 15.58564N, 61.47210W, 30 May–7 June 2011, Malaise, M.A. & L.L. Ivie. **Montserrat**: 3 ♂, 4 ♀, Hope Ghaut, 300 m, 16°45.108'N, 62°12.695'W, 20 June 2017, J.B. Runyon; 5 ♂, 3 ♀, same as previous, 280 m, 16°45.101'N, 62°12.760'W; 6 ♂, 6 ♀, Runaway Ghaut, roadside springs, 150 m, 16°45.449'N, 62°13.011'W, 22 June 2017, J.B. Runyon; 1 ♂, Runaway Ghaut, 175 m, 16°45.43'N, 62°12.89'W, 23 June 2017, J.B. Runyon; 1 ♂, Bottomless Ghaut, 400 m, 16°45.994'N, 62°11.497'W, 28 June 2017, J.B. Runyon. **St. Lucia**: 2 ♂, 1 ♀, Micoud District, Latille Falls, 50 m, 13°49.94'N, 60°55.14'W, 9 May 2009, J.B. Runyon (MTEC, USNM).

###### Distribution.

Dominica, Grenada, Montserrat, St. Lucia.

###### Remarks.

Males of this species have a distinctive long, usually wavy-tipped ventral seta near base of femur II. There is considerable variation in several characters in male specimens among islands, most notably in the modifications at posterior edge of male abdominal sternite III. Specimens from Dominica have the sclerotized lateral lobes of sternite III narrow, but those from Montserrat are broadly rounded. It seems likely that a *P.frater* species complex exists in the Lesser Antilles, but examination of more specimens from more islands is needed to assess limits of variation.

### Subfamily Diaphorinae

#### Genus *Achradocera* Becker

##### Achradocera apicalis

Taxon classificationAnimaliaDipteraDolichopodidae

(Aldrich)

60B2E70F-F183-5EF4-BCA9-C40437D04DAD

Fig. 18A, C

Chrysotus apicalis Aldrich, 1896: 330.
Achradocera angustifacies Becker, 1922: 207.

###### Material examined.

***Lectotype*** (designated here to fix identity of the species) ♂, labelled: “St. Vincent/ W. Indies.”; “Collection/ JM Aldrich”; “Cotype/ No.50426/ U.S.N.M.”; “*Chrysotus*/ *apicalis*/ Type Ald. [hand written]”; “LECTOTYPE/ ♂ *Achradocera*/ *apicalis* (Aldrich)/ des. JB Runyon” [red label] (USNM). **Dominica**: 5 ♂, 2 ♀, Springfield Estate, FIT trap, 29 May 2011, M.A. & L.L. Ivie; 1 ♂, same as previous, Malaise trap, 29 May–1 June 2011; 3 ♂, same as previous, yellow pan traps, 1–3 June 2011. **Montserrat**: 6 ♂, 2 ♀, Woodlands, Riverside House, 10–12 January 2002, Malaise trap, Ivie, Marske & Puliafico; 6 ♂, 1 ♀, same as previous, 5–7 January 2002; 3 ♂, same as previous, 17–28 July 2005, WIBF group; 3 ♂, Hope Ghaut, 8–10 January 2002, yellow pan traps, K.A. Marske; 1 ♂, Cassava Ghaut, Beattie House, 21 January–5 February 2002, Malaise trap, A. Krakower; 2 ♂, same as previous, 24 June 2005, yellow pan traps, V.G. Martinson; 3 ♂, Sweetwater Ghaut, 1 August 2005, yellow pan traps, V.G. Martinson; 3 ♂, Bottomless Ghaut, 5 August 2005, yellow pan traps, V.G. Martinson; 1 ♂, trail to Fairy Walk, 15 August 2005, yellow pan traps, V.G. Martinson; 2 ♂, Cedar Ghaut, 4 August 2005, yellow pan traps, V.G. Martinson; 1 ♂, Big River, 5 August 2005, yellow pan traps, V.G. Martinson. **Nevis**: 5 ♂, Camps River Ghaut, 17°11.36'N, 62°34.66'W, 25 May 2017, J.B. Runyon; 3 ♂, small pond, 200 m, 17°07.460'N, 62°35.584'W, 26 May 2017, J.B. Runyon; 1 ♂, The Source trail, 400–550 m, 17°08.76'N, 62°34.31'W, 26 May 2017, J.B. Runyon. **St. Lucia**: 6 ♂, 2 ♀, Escap Community, small stream in dry forest, 45 m, 13°49.92'N, 60°53.91'W, 2–3 May 2009, J.B. Runyon; 5 ♂, Savannes, Mangrove Reserve, 0–5 m, 13°45.97'N, 60°54.88'W, 3 May 2009, J.B. Runyon; 1 ♂, 1 ♀, trail, dry forest, 45 m, 13°49.9'N, 60°53.9'W, 6 May 2009, J.B. Runyon; 2 ♂, 1 ♀, Doree River ravine, 220 m, 13°47.962'N, 61°01.100'W, 7 May 2009, J.B. Runyon; 1 ♂, Fond Bay near beach, 13°49.89'N, 60°53.65'W, 8 May 2009, J.B. Runyon (MTEC, USNM).

###### Distribution.

Widespread in the West Indies, also reported from Mexico, Ecuador, Chile, and has recently dispersed (probably via accidental human introduction) to French Polynesia and Tonga (Bickel 2000).

###### Remarks.

**Figure 18.**
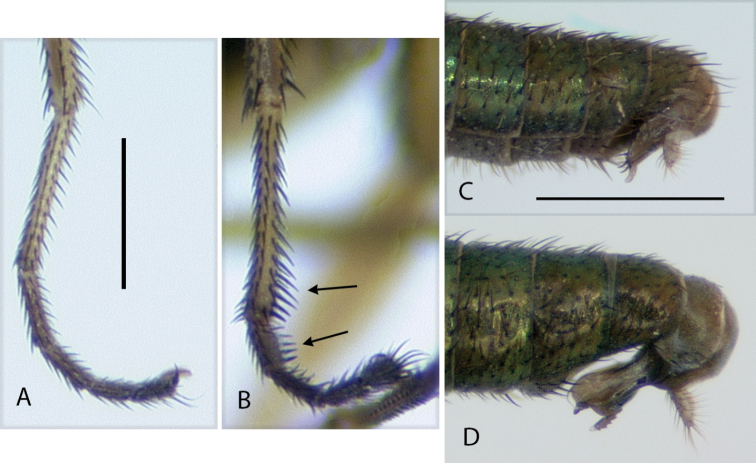
Tarsus I and terminalia of males **A***Achradoceraapicalis* (Aldrich), tarsus I, posterior view (Montserrat) **B***Achradocerabarbata* (Loew), tarsus I, posterior view (Kentucky, USA), arrows indicate ventral setae **C***Achradoceraapicalis*, tip of abdomen, left lateral (Montserrat) **D***Achradocerabarbata*, tip of abdomen, left lateral (Florida, USA). Scale bars: 0.25 mm (**A, B**), 0.5 mm (**C, D**).

Aldrich (1902) synonymized *Achradoceraapicalis* (Aldrich) with *A.barbata* (Loew), primarily due to the distinctive femoral coloration of both species. Robinson (1975) considered *A.apicalis* distinct by the much shorter antenna of the male. Bickel (2000) followed Aldrich (1902) and treated *A.apicalis* and *A.barbata* as synonyms, based again in part on femoral coloration and variation in male antennal length. However, comparison of photos of the holotype of *A.barbata* (MCZ) and specimens from North America (Alabama, Florida, Georgia, Indiana, Kentucky, Missouri, New York, Pennsylvania, Texas, South Carolina, Virginia) with *A.apicalis* from the West Indies (Dominica, Montserrat, Nevis, St. Lucia, St. Vincent) reveals that these species are distinct, most notably in the form of the male front tarsus and genitalia (Fig. 18). The *A.barbata* type and male specimens from North America all have a distinct series of ventral setae on tarsus I (Fig. 18B) whereas specimens from the West Indies (including syntypes from St. Vincent) lack these setae (Fig. 18A). The surstylus and phallus are also very different, with *A.barbata* having the surstylus enlarged and rounded apically and phallus dorsally serrate (Fig. 18D; Bickel 2000: fig. 1a) but in *A.apicalis* the surtylus is smaller and pointed apically and the phallus is not serrate (Fig. 18C; Bickel 2000: fig. 1e as *A.barbata*). The antennal length in males of *A.apicalis* is highly variable, as noted by Bickel (2000), but in all available specimens is distinctly shorter than the antenna in males of *A.barbata*. Therefore, *A.apicalis* (Aldrich) is here removed from synonymy with *A.barbata* (Loew). The species described in Bickel (2000) as *A.barbata* is *A.apicalis*.

#### Genus *Asyndetus* Loew

##### Key to the species of *Asyndetus* in Montserrat

**Table:** 

1	Tibiae I and II dark brown to black usually with distinct metallic green reflections; tibia III with ≥12 large setae (excluding apicals); vein R_1_ reaching over half distance to tip of R_2+3_; body size > 3.5 mm (usually 4.0–6.0 mm); usually associated with crab holes	***A.interruptus* (Loew)**
–	Tibiae I and II usually yellow (sometimes brownish), with little or no distinct metallic green coloration; tibia III with <8 distinct relatively small setae (excluding apicals); vein R_1_ not reaching quite half distance to tip of R_2+3_; body size usually < 3.0 mm	***A.fratellus* Aldrich**

##### Asyndetus interruptus

Taxon classificationAnimaliaDipteraDolichopodidae

(Loew)

72E1CE43-ECE1-570F-A4FA-28F123DE3733

Diaphorus interruptus Loew, 1861: 37.
Asyndetus interruptus Loew, 1869: 37.
Asyndetus bredini Robinson, 1975: 68.
Asyndetus wirthi Robinson, 1997: 479, syn. nov.

###### Material examined.

**Belize**: 2 ♂, 5 ♀, Stann Creek District, beach at Hopkins, 0–2 m, 16°51.16'N, 88°16.73'W, 23 April 2015, JB Runyon; 10 ♂, 3 ♀, same as previous, 18 March 2019. **British Virgin Islands**: 1 ♂, Prickly Pear Island, 18°30.18'N, 64°22.00'W, 3 November 2016, JB Runyon. **Montserrat**: 20 ♂, 8 ♀, Woodlands Beach, 16°45.75'N, 62°13.42'W, 20 June 2017, J.B. Runyon; 1 ♂, 2 ♀, Old Road Bay (beach), 16°44.623'N, 62°14.035'W, 22 June 2017, J.B. Runyon; 2 ♂, Fox’s Bay Beach, 16°43.59'N, 62°14.17'W, 24 June 2017, J.B. Runyon; 1 ♀, Rendezvous Bay Beach, 16°48.489'N, 62°12.296'W, 23 June 2017, J.B. Runyon; 2 ♀, Rendezvous Bay, 26 July 2005, yellow pan trap, V.G. Martinson. **Nevis**: 7 ♂, Winward Beach, 17°06.96'N, 62°32.91'W, 28 May 2017, J.B. Runyon. **St. Kitts**: 3 ♂, Majors Bay, 17°13.62'N, 62°38.91'W, 2 June 2017, J.B. Runyon; 9 ♂, 4 ♀, North Friar’s Bay, 17°16.59'N, 62°40.33'W, 24 May 2017, J.B. Runyon. **St. Lucia**: 5 ♂, 3 ♀, Savannes, Mangrove Reserve, 0–5 m, 13°45.97'N, 60°54.88'W, 3 May 2009, J.B. Runyon; 3 ♂, Micoud District, Fond Bay at beach, 0–5 m, 13°49.89'N, 60°53.65'W, 8 May 2009, J.B. Runyon (MTEC, USNM).

###### Distribution.

Widespread, can be found on beaches in the southeastern United States (Florida), Central America (Belize), Ecuador (Galápagos Islands), and the West Indies (Antigua, British Virgin Islands, Cuba, Dominica, Jamaica, Montserrat, Nevis, St. Kitts, St. Lucia).

###### Remarks.

Initially, I thought both *A.interruptus* and *A.wirthi* Robinson, very similar species (Robinson and Deyrup 1997), were represented in material from Montserrat. Specimens of *A.interruptus* show a wide range of intraspecific variability as discussed by Bickel and Sinclair (1997). The large series from Woodlands Beach shows a gradation in characters used by Robinson and Deyrup (1997) to distinguish *A.interruptus* and *A.wirthi* (e.g., relative length to width of face, shape of antenna, presence of coppery median band between rows of dorsocentral setae). Dissection and examination of male genitalia of *interruptus*-morphotype and *wirthi*-morphotype specimens from Woodlands Beach reveal no obvious differences and both match the illustration in Bickel and Sinclair (1997). Thus, *A.wirthi* is considered a synonym of *A.interruptus*.

*Asyndetusinterruptus* adults were seen mostly on open sand almost always near crab holes and most specimens were obtained by quickly placing a net over crab holes to catch adults as they promptly flew out. This species was re-described and illustrated by Bickel and Sinclair (1997).

##### Asyndetus fratellus

Taxon classificationAnimaliaDipteraDolichopodidae

Aldrich

7645A012-2E4A-5154-A2E1-7650461B0E76

Fig. 19

Asyndetus fratellus Aldrich, 1896: 332.

###### Material examined.

***Lectotype*** (designated here to fix identity of the species) ♂, St. Vincent, W. Indies, Collection J.M. Aldrich, *Asyndetusfratellus* Type Ald., “LECTOTYPE/ ♂ *Asyndetus*/ *fratellus* Aldrich/ des. JB Runyon” [red label] (USNM, specimen number USNMENT01519227). **British Virgin Islands**: 1 ♂, 1 ♀, Guana Island, sand pit Malaise, 15–21 October 2001, B. & B. Valentine; 3 ♀, same as previous, Malaise, 23–25 October 2000; 1 ♂, same as previous, East end, white beach, 2–10 October 2002, R.R. Snelling; 4 ♂, 2 ♀, Eustatia Island, Main Beach, pan traps, 18°30.59'N, 64°21.41'W, 31 October 2016, J.B. Runyon; 2 ♂, 2 ♀, same as previous, Baby Beach, 18°30.63'N, 64°21.57'W, 28–30 October 2016; Prickly Pear Island, salt pond edge, pan traps, 18°30.18'N, 64°21.99'W, 3 November 2016, J.B. Runyon; 7 ♂, 2 ♀, Virgin Gorda, Bitter End Yacht Club, sandy ground near beach, 18°30.13'N, 64°21.30'W, 8–10 November 2016, J.B. Runyon. **Montserrat**: 1 ♂, Woodlands Beach, 16°45.75'N, 62°13.42'W, 21 June 2017, J.B. Runyon; 4 ♂, 6 ♀, Old Road Bay (beach), 16°44.623'N, 62°14.035'W, 22 June 2017, J.B. Runyon; 2 ♂, 2 ♀, Fox’s Bay Beach, 16°43.59'N, 62°14.17'W, 24 June 2017, J.B. Runyon; 1 ♀, Rendezvous Bay Beach, 16°48.489'N, 62°12.296'W, 23 June 2017, J.B. Runyon; 1 ♀, Rendezvous Bay, 26 July 2005, yellow pan trap, V.G. Martinson. **Nevis**: 14 ♂, 17 ♀, Winward Beach, 17°06.96'N, 62°32.91'W, 28 May 2017, J.B. Runyon. **Puerto Rico**: 2 ♂, 5 ♀, Culebra, Flamenco Beach, 27 December 2001, M. Huben. **St. Kitts**: 4 ♂, 3♀, Majors Bay, on *Ipomoea*, 17°13.624'N, 62°38.908'W, 21 May 2017, J.B. Runyon; 1 ♂, 2 ♀, North Friar’s Bay, 17°16.59'N, 62°40.33'W, 24 May 2017, J.B. Runyon; 1 ♂, South Frigate Bay, 17°16.869'N, 62°41.201'W, 24 May 2017, J.B. Runyon. **St. Lucia**: 18 ♂, 26 ♀, Savannes, Mangrove Reserve, 0–5 m, 13°45.97'N, 60°54.88'W, 3 May 2009, J.B. Runyon; 5 ♂, 5 ♀, Micoud District, Fond Bay at beach, 0–5 m, 13°49.89'N, 60°53.65'W, 8 May 2009, J.B. Runyon (MTEC, USNM).

###### Re-description, based on material from Montserrat.

**Male.** Body length 2.2–2.7 mm (body size of some specimens from St. Kitts and Nevis approach 3.5 mm), wing length 1.7–2.1 × width 0.7–1.0 mm. ***Head***: Face as wide as frons, parallel-sided, slightly higher than wide, covered with dense white pruinosity that obscures ground color. Frons with dense grayish white pruinosity, obscuring ground color; vertical setae proclinate. Palpus black with sparse white pruinosity, with black setae, a couple larger setae near apex. Proboscis black. Antenna black; pedicel somewhat produced above and on sides; first flagellomere short, wider than long, rounded apically; arista-like stylus inserted near middle of dorsal edge. Lower postocular setae white. ***Thorax***: Scutum dark metallic green-blue with dense white pruinosity, with distinct band of coppery brown pruinosity between dorsocentral rows becoming slightly broader posteriorly and ending at scutellum and coppery brown pruinose area above wing bases; 1–6 pairs of irregularly biseriate acrostichal setae, often missing on anterior half of scutum; five pairs of dorsocentral setae; scutellum with one pair of large marginal setae and one pair of small lateral setae. Pleuron dark metallic bluish green with dense grayish white pruinosity; with two small black setae on lower proepisternum and one or two small black setae on upper proepisternum. ***Legs***: Hairs and setae mostly black. Coxae concolorous with pleuron; coxae I and II with black setae anteriorly; coxa III with black lateral seta near base and small brown lateral seta near 2/3. Femora dark brown to black with extreme tips yellow, with av and pv rows of longer rather slender dark setae ventrally (longest ca. half width of femur) that can appear yellowish in certain lights, and with a few stouter av and pv setae near apex; femora II and III also with slightly larger anterior setae near apex. Tibiae I and II yellow (some specimens from British Virgin Islands, Puerto Rico, St. Lucia, and St. Vincent have tibia I and/or II varying degrees of brown), tibia II usually brownish at very base, tibia III brown but sometimes yellowish basally; tibia I with small area of close-set pale setulae av on apical half, with small setae, an ad seta near 1/3, pd seta near 1/3, 1/2, near apex and apical ad, posterior, and pv seta; tibia II with large ad seta near 1/5 and 3/5, large pd seta just before 1/5, 1/2, near 3/5, smaller ventral seta near 3/5, and 3–4 large apical setae, the ventral one largest; tibia III with large setae, ad seta at 1/5, just beyond 1/2 and sometimes smaller seta near 2/5, with five or six pv small setae of varying lengths rather evenly spaced along length of tibia, no ventral setae, four apical setae the dorsal seta largest. Tarsomeres I(1) and II(1) with apex brown, tarsi otherwise dark brown; tarsomere 5 of each leg with apical fan of small black dorsal setae. Tarsomere I(5) slightly broadened. Tarsal claws absent, pulvilli white and enlarged on all legs. Ratios of tibia:tarsomeres: leg I: 32–14–8–6–4–5; leg II: 40–18–10–7–4–5; leg III: 45–13–12–9–6–5. ***Wing***: Hyaline but with slight whitish sheen and brown veins, oblong-elliptical with prominent anal lobe. Veins R_2+3_ and R_4+5_ rather close together, subparallel but slightly diverging apically, both joining costa before wing apex; R_4+5_ nearly straight to scarcely bent backwards at apex. Distal section of M free and offset from basal section (rarely these sections are indistinctly connected via a thin trace of vein M; basal and distal sections of M overlap in a few female specimens from St. Kitts and Nevis). Crossvein dm-cu placed near basal 1/3 of wing length, ca. one-seventh as long as last part of CuA_1_. Calypter white with white setae. Halter stem yellow-brown and knob white. ***Abdomen***: Cylindrical, dark metallic green (some specimens with distinct copper reflections) obscured by grayish white pruinosity that is thickest laterally. Tergites covered with numerous small black setae that are longer laterally and along distal margins; tergite VI mostly to completely hidden, bare. Sternites with sparse but rather long setae that can appear brownish. Sternite VIII with four short but stout setae projecting posteriorly from apex of preabdomen. Hypopygium small, dark brown, enclosed in tip of abdomen. Epandrium dark brown, nearly round. Surstylus bilobed; dorsal lobe shining dark brown, as long as epandrium, narrow, broadest basally with slightly expanded apex, with distinct dorsal seta near 2/3 (and sometimes a second smaller neighboring seta) and minute hairs apically; ventral lobe of surstylus half as long as dorsal lobe, subtriangular, with distinct seta at apex subtended by one or two smaller setae and medially near base with a papilla bearing a seta. Cercus dark brown, small, nearly round, covered with small black setae of nearly uniform length.

**Female.** Body length 2.6–2.9 mm, wing length 2.1–2.4 × width 0.8–1.1 mm. Similar to male, but face slightly wider; clypeus distinct, bulging slightly; femora II and III without longer ventral setae but av row on femur I distinct; tibia III often yellowish on basal half; each tarsomere 5 without fan of black dorsal setae; pulvilli small; short distinct claws present.

###### Distribution.

British Virgin Islands, Grenada, Jamaica, Montserrat, Nevis, Puerto Rico, St. Kitts, St. Lucia, St. Vincent.

###### Remarks.

**Figure 19.**
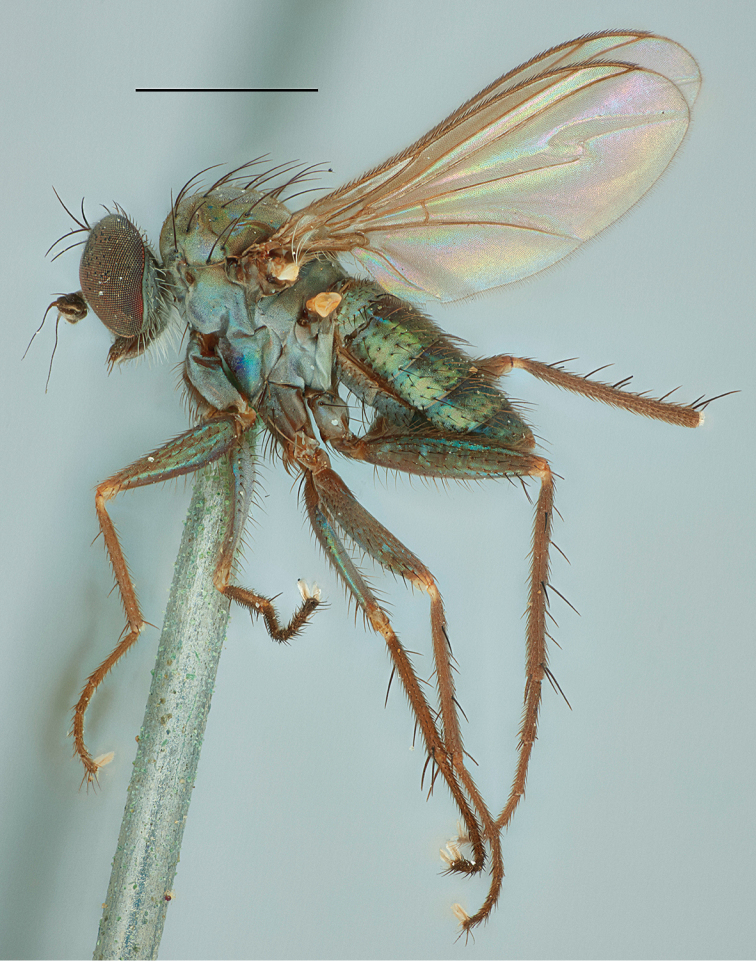
Lectotype male of *Asyndetusfratellus* (St. Vincent), left lateral (specimen number USNMENT01519227). Scale bar: 1.0 mm. Photo taken by Alyssa Seemann (USNM).

All specimens from Montserrat have yellow tibiae I and II, and because of this I at first suspected these represented an undescribed species. The only other species of *Asyndetus* known from the West Indies reported to have tibiae I and II yellow is *A.syntormoides* Wheeler which has an enlarged first flagellomere and vein M delicate but complete throughout (Wheeler 1899: figs 50–52). However, examination of material from Puerto Rico to St. Lucia (see Material examined) reveals the color of tibiae I and II varies from yellow to dark brown. Specimens from islands north of Montserrat generally have tibia I yellow with tibia II often yellow but frequently brown (a few specimens also have tibia I brown), but specimens southward usually have all tibia brownish, including the lectotype from St. Vincent (Fig. 19; a few specimens from St. Lucia have tibia I yellow). Other characters are variable including body size (2.0–3.5 mm), number/extent of acrostichal setae, and size of ventral setae on femora II and III. I can find no characters to reliably distinguish these specimens, and thus consider them conspecific, and interpret *A.fratellus* as a littoral species widespread in the West Indies. However, a revision of this genus in the Neotropics is needed. Two species similar to *A.fratellus* were described from Dominica (*A.dominicensis* Robinson) and Puerto Rico (*A.deficiens* Robinson) that might prove conspecific. Outside the West Indies, *A.currani* Van Duzee (Panama, photos of holotype examined) is very similar and might also prove conspecific, but the holotype has ventral hairs on femora more yellowish and wing with R_4+5_ slightly more bent backwards at apex.

Many adults of *A.fratellus* were collected from leaves of beach morning glory (*Ipomoeapes-caprae*).

#### Genus *Chrysotus* Meigen

##### Key to the species of *Chrysotus* in Montserrat (males)

**Table:** 

1	First flagellomere broad, 2–3 × as wide as pedicel, with base projecting above base of pedicel (as in Fig. 27), which is distinctly produced on inner side	**2**
–	First flagellomere scarcely broader than pedicel, which is not distinctly longer on inner side	**5**
2	Tarsus III(1) with conspicuous ventral seta near 1/2	***C.spinipes* Van Duzee**
–	Tarsus III(1) without conspicuous ventral seta	**3**
3	Tarsus III(2) prolonged posteriorly in spur overlapping tarsus III(3) (Fig. 28B)	***C.montserratensis* sp. nov.**
–	Tarsus III(2) without spur-like projection	**4**
4	First flagellomere with deep rectangular apical notch where arista-like stylus is inserted; tibiae wholly yellow; halter knob yellow	***C.proximus* Aldrich**
–	First flagellomere only slightly depressed where arista-like stylus is inserted; tibiae II and III brownish; halter knob brown	***C.integer* Robinson**
5	Male face broad, eyes not contiguous below antennae	**6**
–	Male face obliterated or nearly so by contiguous eyes	**11**
6	Tergite VI bare except 1 distolateral seta at lower margin; males with tarsal claws absent on all legs	**7**
–	Tergite VI covered with numerous setae; males with all legs with at least 1 claw	**8**
7	Male frons as wide as face, dorsal ommatidia not enlarged (Fig. 25C); ad seta near base of tibia II large (length > width of tibia)	***C.parvulus* (Aldrich)**
–	Male frons narrower than face, dorsal ommatidia noticeably enlarged (Fig. 25B); ad seta near base of tibia II small (length < width of tibia)	***C.interfrons* sp. nov.**
8	Abdomen deep bluish violet; males with all legs with only 1 claw; epandrium with bulbous basodorsal protuberance (Fig. 23)	**9**
–	Abdomen metallic green to bronze or brown; males with 2 claws on leg III; epandrium evenly rounded basally and dorsally	**10**
9	Halter knob brown; setae on the thorax, legs, abdomen, and calypter dark brown to black; sheath of phallus with 1 large apical spine	***C.callichromus* Robinson**
–	Halter knob white; setae on the thorax, legs, abdomen, and calypter mostly white to pale brown; sheath of phallus with 3 large apical spines (Fig. 23)	***C.callichromoides* sp. nov.**
10	Eyes broadly separated above antennae; palpus yellow	***C.angustifrons* (Robinson)**
–	Eyes contiguous above antennae; palpus mostly brown	***C.spectabilis* (Loew)**
11	Coxa I half or more yellow	**12**
–	Coxa I mostly to wholly brown to black	**16**
12	Palpus yellow, narrow, very long (length subequal to head height); first flagellomere yellow	***C.xiphostoma* Robinson**
–	Palpus short; first flagellomere brown	**13**
13	First flagellomere prolonged into slender tip bearing numerous long hairs; pleuron yellow	***C.microtatus* Meuffels & Grootaert**
–	First flagellomere short, not or scarcely longer than wide; pleuron dark	**14**
14	Scape and pedicel yellow; coxa I with rather long yellow setae; femur III yellow and with 2 erect ventral setae at base	***C.brevicornis* Van Duzee**
–	Antenna wholly brown; coxa I with brown to black setae; femur III with apical half partly to wholly brown and without distinct ventral setae at base	**15**
15	Palpus yellow, exserted, elongate oval, bare except for a strong seta at apex; tibia and tarsus III with many long, erect setae covering anterior surface; hypopygium enlarged, abdomen not tapering	***C.hirsutus* Aldrich**
–	Palpus yellow, small and partly hidden, covered with several small setae; tibia and tarsus III without unusual setae; hypopygium small, abdomen noticeably tapering	***C.antillensis* sp. nov.**
16	First flagellomere triangular and prolonged (length ca. 2.5 × basal width) with apex deeply cleft and arista-like stylus inserted between 2 narrow projections, the ventral longer; tibia I whitish-yellow with white setae posteriorly; halter knob brown	***C.acutus* Aldrich**
–	First flagellomere not prolonged or cleft apically; tibia I without white setae posteriorly; halter knob yellow or brown	**17**
17	Palpus rounded, white; wing veins R_4+5_ and M_1_ slightly diverging distally; male abdomen stout, broadened to tip; cercus prominent	**18**
–	Palpus black; wing veins R_4+5_ and M_1_ parallel to slightly convergent distally; male abdomen gradually tapered; cercus small	**19**
18	Wing vein M_1_ straight distally; cercus narrowly oval bearing only slender setae	***C.mediocaudatus* Robinson**
–	Wing vein M_1_ curving backward near tip; cercus broad, rather appressed, sclerotized and bearing 2 or 3 stout apical setae	***C.lamellicaudatus* Robinson**
19	Legs wholly brown to black; tibia II with small ad seta, without pd setae; wing with crossvein dm-cu two-thirds as long as last part of CuA_1_; mesoscutum with dense black pruinosity; female face slightly narrowed below	***C.orichalceus* Gosseries**
–	Legs partly yellow or white; tibia II with large ad seta, and usually with 2 small pd setae; wing with crossvein dm-cu half as long as last part of CuA_1_; mesoscutum without black pruinosity; female face parallel-sided	**20**
20	Femora mostly dark brown with dark setae; female with lower postocular setae pale	***C.pseudoniger* Robinson**
–	Femora yellow with femur III partly brown, with many white setae; female with lower postocular setae mostly dark	***C.albihirtipes* Robinson**

##### Chrysotus acutus

Taxon classificationAnimaliaDipteraDolichopodidae

Aldrich

FE9E1F9B-C69A-5E55-A9BD-5F1F5AF1D43B

Chrysotus acutus Aldrich, 1896: 329.

###### Material examined.

**Montserrat**: 3 ♂, Bottomless Ghaut to Big River trail, 14 August 2005, yellow pan traps, V.G. Martinson; 1 ♂, Big River, 5 August 2005, yellow pan traps, V.G. Martinson; 2 ♂, ghaut above Montserrat Volcano Observatory, 330 m, 16°45.130'N, 62°12.487'W, 27 June 2017, J.B. Runyon (MTEC, USNM). **St. Vincent**: Syntype ♂ (USNM).

###### Distribution.

Lesser Antilles (Dominica, Montserrat, St. Vincent). Reports of this species in Central America are probably incorrect (Robinson 1975).

##### Chrysotus albihirtipes

Taxon classificationAnimaliaDipteraDolichopodidae

Robinson

548A46C5-8BC2-5850-A230-F4B780F737E2

Chrysotus albihirtipes Robinson, 1975: 90.

###### Material examined.

**Dominica: *Holotype*** ♂, Boeri Lake trail, 22 February 1964, H. Robinson (USNM). **Montserrat**: 1 ♂, Runaway Ghaut, 175 m, 16°45.43'N, 62°12.89'W, 23 June 2017, J.B. Runyon (MTEC).

###### Distribution.

Dominica, Montserrat.

##### Chrysotus angustifrons

Taxon classificationAnimaliaDipteraDolichopodidae

(Robinson)

0E6755F1-ED2A-5AE8-A88D-81CC1468618C

Diaphorus angustifrons Robinson, 1975: 93.
Dubious angustifrons (Robinson) [unwarranted combination by Wei 2012: 611].

###### Material examined.

**Dominica**: 1 ♂, Springfield Estate, yellow pans, 1–3 June 2011, M.A. & L.L. Ivie. **Montserrat**: 2 ♂, 2 ♀, Fox’s Bay Beach, 16°43.59'N, 62°14.17'W, 23 June 2017, J.B. Runyon (MTEC, USNM).

###### Distribution.

Dominica, Montserrat.

###### Remarks.

This species was transferred to *Chrysotus* and re-described and illustrated by Capellari and Amorim (2010). Wei (2012) proposed placement of this species in a new genus, *Dubius* Wei, but this appears to be unwarranted as discussed by Capellari and Amorim (2014).

##### Chrysotus antillensis

Taxon classificationAnimaliaDipteraDolichopodidae

sp. nov .

F2723C20-097F-5027-8C5B-513209C44BF7

http://zoobank.org/5B0B8171-7B2F-4BC0-9F16-04047BFDB5F0

Figs 20 , 21

###### Type material.

***Holotype***, ♂ labelled: “DOMINCA: St. John Par./ Cabrits N.P. (Malaise)/ East Cabrits Trail/ 15.58564N, 61.47210W/ 30MAY–07JUNE 2011/ M.A. & L.L. Ivie”; “HOLOTYPE/ ♂ *Chrysotus*/ *antillensis*/ Runyon [red label]” (USNM, type number USNMENT01350612). ***Paratypes***: 41 ♂, 1 ♀, same data as holotype. **Montserrat**: 1 ♂, Woodlands, Riverside House, 10–12 January 2002, Malaise trap, Ivie, Marske, Puliafico**. Nevis**: 6 ♂, 2 ♀, Recreation ground, 134 m, 17°07.507'N, 62°34.446'W, 31 August 2017, fogging; 1 ♂, St. John Parish, small pond, 200 m, 17°07.460'N, 62°35.584'W, 26 May 2017, J.B. Runyon. **St. Lucia**: 5 ♂, 2 ♀, near Micoud, trail to Fond Bay, 15 m, 13°49'48"N, 60°53'42"W, 16–22 May 2009, Malaise trap, S.D. Gaimari & A.R. Cline; 1 ♂, Grande Anse, 38 m, 14.00519N, 60.89737W, Malaise trap, 13–23 May 2009, R. Winton & E. Ivie; 1 ♂, Grande Anse, 14.00529N, 60.89737W, FIT, 23–26 May 2009, C.A. Maier & R.C. Winton (MTEC, USNM).

###### Other material examined.

**British Virgin Islands**: 1 ♂, 9 ♀, Tortola, 425 m, 18°25.35'N, 64°38.67'W, 6 November 2016, J.B. Runyon; 1 ♀, Eustatia Island, Baby Beach, 18°30.64'N, 64°21.57'W, 28 October 2016, J.B. Runyon; 1 ♂, 1 ♀, Guana Island, sand pit Malaise, 15–21 October 2001, B. & B. Valentine.

###### Description.

**Figure 20.**
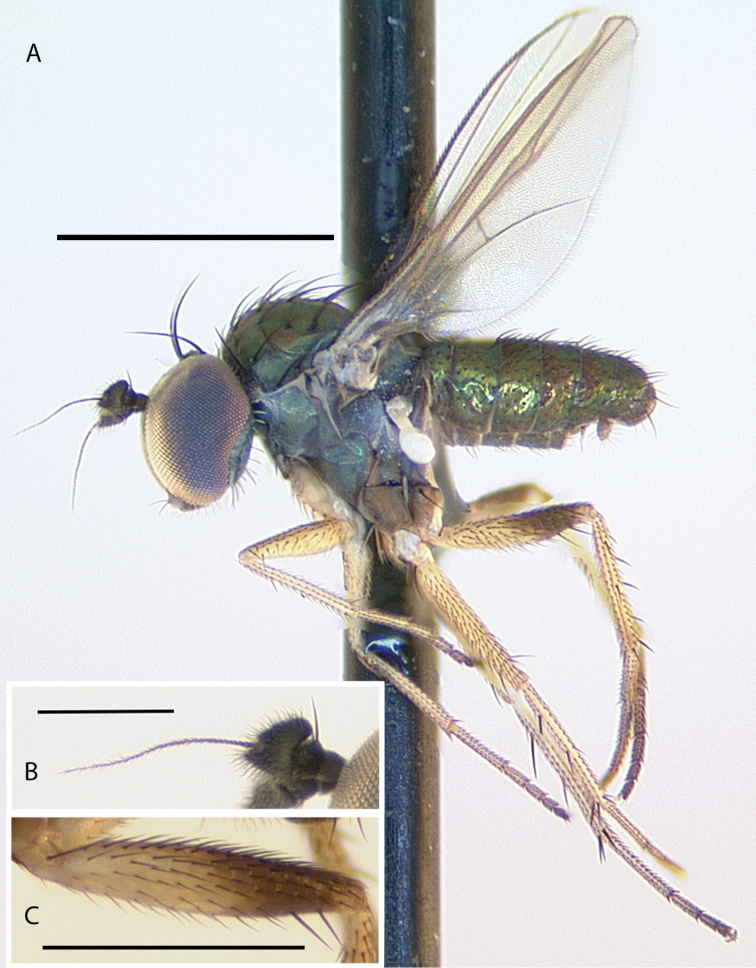
*Chrysotusantillensis* sp. nov., male **A** habitus of male holotype, left lateral **B** antenna, left lateral **C** femur III, anterior. Scale bars: 1.0 mm (**A**), 0.25 mm (**B**), 0.5 mm (**C**).

**Figure 21.**
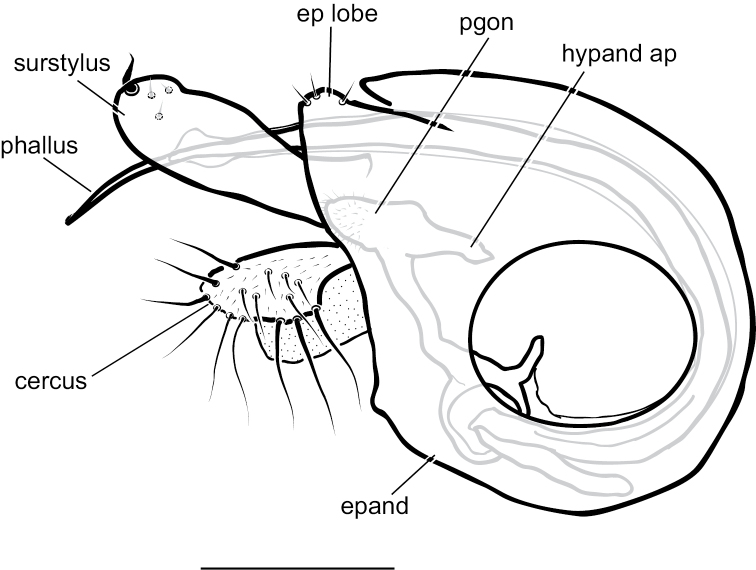
*Chrysotusantillensis* sp. nov. male terminalia, left lateral. Abbreviations: epand-epandrium; ep lobe-epandrial lobe; hypand ap-hypandrial apodeme; pgon-postgonite. Scale bar: 0.1 mm.

**Male** (Fig. 20A). Body length 1.9–2.1 mm, wing length 1.6–1.7 × width 0.6–0.7 mm. ***Head***: Eyes contiguous below, with anterior ommatidia enlarged; upper face narrowly triangular, metallic green with dense light brown pruinosity. Frons metallic green-blue with sparse light brown pruinosity and minor bronze reflections. Postcranium with dense light brown pruinosity. Palpus small, oval, yellow, covered with minute yellow hairs and a pale brown to black dorsal subapical seta. Proboscis dark yellow to brown with fine pale to brown hairs along margin. Antenna (Fig. 20B) black; scape rather long, cylindrical; pedicel shorter than scape, with apical ring of small setae, and a larger apical seta dorsally; first flagellomere subtriangular, rounded dorsally at base and overlapping pedicel, width subequal to length; arista-like stylus subapical, inserted just lateral and dorsal to apex in a small notch. Postocular setae white. ***Thorax***: Scutum and scutellum metallic green with strong bronze reflections and sparse light brown pruinosity; postpronotal lobe with a small yellow spot at lateral corner; eight pairs of small biseriate acrostichal setae; six pairs of dorsocentral setae, anterior-most pair small; scutellum with one pair of large marginal setae and one pair of small lateral setae. Pleuron metallic bluish green with dense gray pruinosity; one or two pale brown setae on lower proepisternum. ***Legs***: Coxa I yellow but usually brownish at very base, with yellow-brown to black setae; coxae II and III nearly concolorous with pleuron but with brown tinge and yellow tips, with pale brown to brown setae. Femora yellow except femur III (Fig. 20C) brown on approximately apical one-third with tip narrowly yellow and with 2–3 distinct av setae near tip; femur II with preapical av and pv seta. Tibia I yellow with small ad seta at 1/4; tibia II yellow with large ad seta near 1/5 and usually a smaller ad seta at 1/2, small pv seta just before 1/5 and 1/2, and with apical ring of four or five setae; tibia III yellow with ad seta at 1/5 and 1/2, a subapical dorsal seta, with pv seta near 1/5, 2/5, 1/2, and 4/5. Tarsi yellow, distal tarsomeres becoming brown, with small claws and very small pulvilli. Ratios of tibia:tarsomeres: leg I: 22–12–5–4–3–4; leg II: 28–13–6–5–3–3; leg III: 34–10–8–5–3–3. ***Wing***: Hyaline, oblong-elliptical, relatively narrow with poorly developed anal lobe. Vein R_4+5_ and M_1_ nearly straight but very slightly diverging near apex. Crossvein dm-cu placed near 2/5 of wing length, ca. one-fourth as long as last part of CuA_1_. Calypter white with white to pale-brown setae. Halter stem and knob yellow. ***Abdomen***: Cylindrical, gradually tapering, with hairs and setae black. Tergites dark metallic green with bronze reflections and little to no pruinosity; tergite VI with numerous small setae. Sternite VIII covering hypopygial foramen, with small setae. Hypopygium (Fig. 21) small, partially embedded in tip of abdomen. Hypopygial foramen left lateral. Epandrium dark brown, rounded with distal margin rather flattened, with broad ventroapical lobe bearing three small setae. Surstylus paddle-shaped, shining brown, with three small medial setae near apex and a larger medial black spine-like seta at apex. Cercus brown, triangular with ventral margin rather straight, with brown hairs and setae. Phallus narrow with rounded, very slightly broadened apex; encircled by external membranous sheath that has small wing-like inflations before apex of phallus. Postgonites covered with microtrichia apically. Hypandrial apodemes short, rather pointed apically in lateral view.

**Female.** Body length 2.1–2.3 mm, wing length 1.9–2.0 × width 0.8–0.9 mm. Similar to male, but face wide (>half width of frons at ocellus) and nearly parallel-sided, covered with dense light gray-brown pruinosity; clypeus distinct, bulging at suture; frons metallic blue-green to violet with very sparse light brown pruinosity; palpi larger, yellow with base brown, covered with yellow microtrichia and a few small brown to black setae; scape short, subequal in length to pedicel; first flagellomere shorter, reniform; abdomen broader, slightly flattened dorsoventrally; wing noticeably broader.

###### Etymology.

This species is named for the Greater and Lesser Antilles.

###### Distribution.

British Virgin Islands, Dominica, Montserrat, Nevis, St. Lucia.

###### Remarks.

The combination of hind femur color and shape of the male first flagellomere of *C.antillensis* is distinctive (Fig. 20). Females are very similar to those of *C.hirsutus* Aldrich, but females of *C.antillensis* have two or three small but distinct ventral setae on tibia III (no outstanding ventral setae in *C.hirsutus*). Given the number of specimens collected in Dominica in 2011, it is perhaps surprising that *C.antillensis* was not discovered during the Bredin-Archbold-Smihsonian survey of Dominica (Robinson 1975). However, four species found on Montserrat that were not included in Robinson (1975) are herein reported from Dominica (*Chrysotusantillensis*, *C.callichromoides*, *Systenusladonnae*, and *Thrypticusmediofuscus*). These four species appear largely restricted to dry forests at lower elevations, suggesting that this habitat type may not have received adequate attention during the Dominica survey.

##### Chrysotus brevicornis

Taxon classificationAnimaliaDipteraDolichopodidae

Van Duzee

448B28DA-0557-5BFB-84D4-01444BDA051E

Chrysotus brevicornis Van Duzee, 1933b: 68.
Chrysotus brevispina Van Duzee, 1933b: 68.
Chrysotus latifacies Van Duzee, 1933b: 69.
Chrysotus mexicanus Robinson, 1967b: 120.

###### Material examined.

**Dominica**: 3 ♂, Springfield Estate, FIT, 29 May 2011, M.A. & L.L. Ivie. **Mexico: *Holotype*** ♂ of *Chysotusmexicanus*, Veracruz, km 375, rt. 180, 7 August 1962, H. Robinson (USNM). **Montserrat**: 29 ♂, 24 ♀, Woodlands, Riverside House, 10–12 January 2002, Malaise trap, Ivie, Marske, Puliafico; 8 ♂, 4 ♀, same as previous, 5–7 January 2002; 3 ♂, Hope Ghaut, 8–10 January 2002, yellow pan traps, K.A. Marske; 1 ♂, Bottomless Ghaut, 5 August 2005, yellow pan traps, V.G. Martinson; 1 ♂, Underwood Ghaut, canopy fogging at dawn, 23 May 2002, K. Marske & J. Boatswain; 8 ♂, 3 ♀, Fox’s Bay Beach, 16°43.59'N, 62°14.17'W, 23 June 2017, J.B. Runyon; 4 ♂, 1 ♀, rental house in Old Town, 16°44.795'N, 62°13.711'W, 19 June 2017, J.B. Runyon (MTEC, USNM).

###### Distribution.

Widespread in the Neotropics, from Mexico to Brazil and throughout the West Indies, and the Galápagos Islands.

###### Remarks.

This species was re-described and illustrated by Bickel and Sinclair (1997). Robinson (1975) treated this species as *C.mexicanus*.

##### Chrysotus callichromoides

Taxon classificationAnimaliaDipteraDolichopodidae

sp. nov .

415BAF40-BF13-5A21-86E6-E05F2A574E62

http://zoobank.org/406317D4-9363-4C91-BABB-68D1D6518D18

Figs 22 , 23

###### Type material.

***Holotype***, ♂ labelled: “MONTSERRAT: Woodlands/ Riverside House, 140 ft/ 16°45.985'N, 62°13.341'W/ 10–12JAN2002, Malaise/ Ivie, Marske, Puliafico”; “HOLOTYPE/ ♂ *Chrysotus*/ *callichromoides*/ Runyon [red label]” (USNM, type number USNMENT01350613). ***Paratypes*: Montserrat**: 2 ♂, 2 ♀, same data as holotype; 1 ♂, same as previous, 5–7 January 2002; 2 ♂, same as previous, 10–13 January 2002, at light; 8 ♂, Cassava Ghaut, Beattie House, 632 ft, 16°45.91'N, 62°12.95'W, 8–17 April 2002, Malaise, A. Krakower; 2 ♂, 1 ♀, same as previous, 17 April–1 May 2002; 1 ♂, same as previous, 17–30 May 2002; 4 ♂, same as previous, 6–12 June 2002; uv light; 10 ♂, 10 ♀, same as previous, 21–30 June 2002, M.A. Ivie; 5 ♂, 7 ♀, same as previous, 14–30 June 2002, Malaise; 5 ♂, same as previous, 24 June 2005, yellow pans, V.G. Martinson; 1 ♂, Rendezvous Bay, 26–31 July 2005, uv light, WIBF group; 1 ♂, Cedar Ghaut, 26–31 July 2005, yellow pans, V.G. Martinson; 1 ♂, Bottomless Ghaut, 5 August 2005, yellow pans, V.G. Martinson; 1 ♂, Old Town, 16°44.795'N, 62°13.711'W, 19 June 2017, J.B. Runyon.

###### Other material examined.

**Dominica**: 4 ♂, 1 ♀, St. John Parish, Cabrits National Park, East Cabrits Trail, 15.58564°N, 61.47210°W, 30 May–7 June 2011, Malaise, M.A. & L.L. Ivie. **Nevis**: 1 ♂, Camps watershed, 17.18972N. 62.57740W, 70 m, Malaise; 1 ♂, same as previous, yellow pans, 25 May 2017, J.B. Runyon; 4 ♂, Recreation ground, 134 m, 17°07.507'N, 62°34.446'W, 31 August 2017, fogging; 2 ♂, Pinney’s Estate, 22 m, 17°08'54.7"N, 62°37'15.8"W, 20 June 2017, fogging. **St. Kitts**: 2 ♂, Majors Bay, 15 m, 17.22713N, 62.65183W, 20 February–3 March 2017, Malaise. **St. Lucia**: 5 ♂, 1 ♀, Micoud District, Escap community, 13°49.92'N, 60°53.91'W, 2–7 May 2009, yellow pans, J. Runyon & C. Delphia; 2 ♂, 1 ♀, Micoud District, trail in dry forest, 45 m, 13°49.9'N, 60°53.9'W, 6 May 2009, J.B. Runyon; 7 ♂, 7 ♀, near Micoud, trail to Fond Bay, 15 m, 13°49'48"N, 60°53'42"W, 16–22 May 2009, Malaise and blacklight trap, S.D. Gaimari & A.R. Cline (MTEC, USNM).

###### Description.

**Figure 22.**
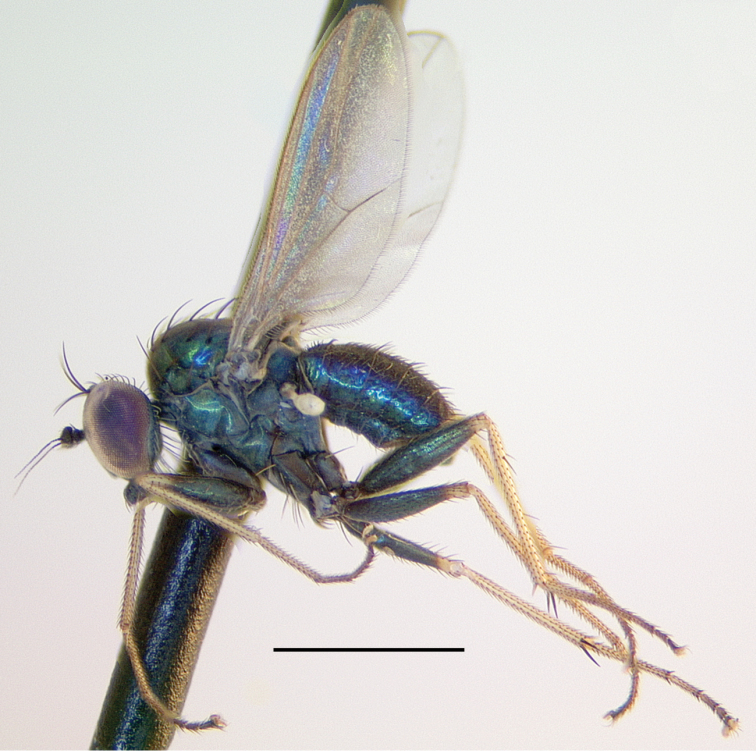
*Chrysotuscallichromoides* sp. nov. habitus of male holotype, left lateral. Scale bar: 1.0 mm.

**Figure 23.**
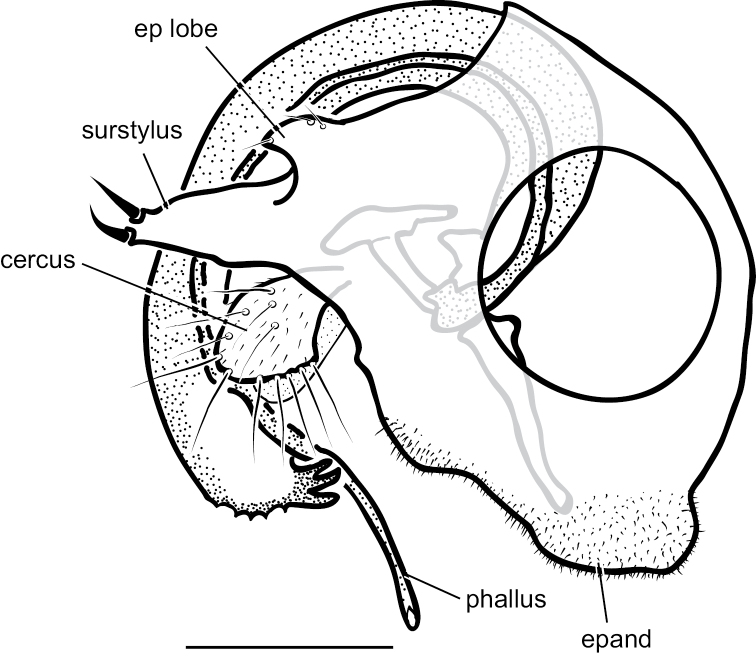
*Chrysotuscallichromoides* sp. nov. male terminalia, left lateral. Abbreviations: epand-epandrium; ep lobe-epandrial lobe. Scale bar: 0.1 mm.

**Male** (Fig. 22). Body length 2.2–2.6 mm, wing length 2.0–2.5 × width 0.8–1.1 mm. ***Head***: Eyes not contiguous below; face only slightly narrowed, at narrowest one-third width of frons at ocelli; face and frons metallic green-blue with yellow-brown pruinosity that is denser along eyes. Palpus black, oval and broadly pointed apically, with sparse silver pruinosity and ca. six yellow-brown to black setae. Proboscis dark brown with fine hairs along margin. Antenna black; first flagellomere short, width ca. 1.5 × length, lower posterior margin rather flat and dorsal margin rounded, slightly receding; arista-like stylus nearly apical, with small pointed projection below insertion. Lower postocular setae white. ***Thorax***: Scutum and scutellum metallic green with distinct violet reflections and sparse light brown pruinosity; setae on scutum pale brown; six pairs of small biseriate acrostichal setae; six pairs of dorsocentral setae, anterior-most pair small; scutellum with one pair of large marginal setae and one pair of small lateral setae. Pleuron metallic bluish green with sparse gray pruinosity; one or two white setae on lower proepisternum. ***Legs***: Coxae dark brown with metallic blue-green reflections and white setae. Femora dark brown with metallic blue-green reflections with yellow apex and yellow to pale brown hairs and setae, with a few small av and pv setae near tips; femur II with four or five small posterior setae on apical one-third. Tibiae yellow with most setae and hairs pale brown; tibia I without distinctive setae; tibia II without distinctive setae except two large ventral setae at apex; tibia III with ad seta near 1/4, smaller pd seta near 1/4, 1/2, and near apex (specimens from St. Lucia usually have another pd seta near 2/5). Tarsi yellow, distal tarsomeres becoming brown, with tarsomere 5 slightly broadened; pulvilli white and slightly enlarged (subequal in size to tarsomere 5), and each leg with just one tarsal claw; tarsomeres I (2–4) ventrally with white pile. Ratios of tibia:tarsomeres: leg I: 25–18–8–6–3–4; leg II: 36–20–9–6–3–4; leg III: 44–16–9–6–4–4. ***Wing***: Hyaline, oblong-elliptical. Costa slightly more thickened than usual between R_1_ and R_4+5_. Vein R_4+5_ nearly straight. Vein M_1_ curving slightly backwards near apex. Crossvein dm-cu ca. two-thirds as long as last part of CuA_1_. Calypter yellow with white to pale-brown setae. Halter knob white, stem brownish at base. ***Abdomen***: Broadly cylindrical, gradually tapering, with hairs and setae pale brown to brown. Tergites dark metallic violet (especially in dorsal view), lateral margins and sternites bluish green; marginal setae only slightly larger; tergite VI with numerous small setae. Sternite VIII with small setae, covering hypopygial foramen. Hypopygium (Fig. 23) small, dark brown to black, positioned in ventral notch at tip of abdomen. Hypopygial foramen left lateral. Epandrium dark brown, rather deeply emarginated apicoventrally, with triangular ventroapical lobe bearing three small setae; basodorsally with bulbous protuberance that is densely covered with minute hairs. Surstylus elongate, shining brown, with two strong spines at apex. Cercus brown, ovoid with apex somewhat pointed and narrowed at base, with rather dense whitish to pale brown hairs and setae. Phallus simple, narrow, strongly arched, strongly sclerotized but less so near rounded apex; with sheath rather broad throughout, broadened near apex with 4–5 small dorsal teeth along dorsal margin and three black apical spines arching ventrally to overlap phallus, strongly sclerotized except membranous subapically in middle of expanded area.

**Female.** Body length 2.2–2.6 mm, wing length 2.0–2.4 × width 0.8–1.0 mm. Similar to male, but face less narrowed below with narrowest width two-thirds width of frons at ocelli; clypeus distinct, bulging at suture; first flagellomere with less prominent point below insertion of arista-like stylus; thorax and abdomen dark green with bronze reflections, not violet; femur I usually more yellow at tip; tibia II with large ad seta near 1/5; tarsi with tarsomere 5 not broadened, pulvilli not enlarged, with two tarsal claws; wing with thickening of costa less pronounced.

###### Etymology.

This species is named for its similarity and presumed relatedness to *C.callichromus* Robinson.

###### Distribution.

Dominica, Montserrat, Nevis, St. Kitts, St. Lucia.

###### Remarks.

*Chrysotuscallichromoides* differs most noticeably from *C.callichromus* in having a white halter knob and white to pale brown setae on the thorax, legs, abdomen, and calypter (each of these has dark brown to black setae in *C.callichromus*). The male genitalia of these two species are very similar, but the phallus sheath in *C.callichromus* has only one apical black spine (three in *C.callichromoides*). The habitat also seems to differ, with *C.callichromoides* mostly found in low elevation dry forests and *C.callichromus* in moist ghauts in mesic forests. *Chrysotusmorrisoni* Van Duzee (Virgin Islands) (holotype examined) is also related but differs in the scutum having dense brownish pruinosity (mostly obscuring cuticle) with only very slight violet reflections.

##### Chrysotus callichromus

Taxon classificationAnimaliaDipteraDolichopodidae

Robinson

47A54AEC-6796-58DF-8386-48540184FD9F

Chrysotus callichromus Robinson, 1975: 79.

###### Material examined.

**Dominica: *Holotype*** ♂, Clarke Hall, light trap, 21–28 February 1965, W.W. Wirth (USNM). **Montserrat**: 3 ♂, ghaut above Montserrat Volcano Observatory, 330 m, 16°45.130'N, 62°12.487'W, 27 June 2017, J.B. Runyon (MTEC, USNM).

###### Distribution.

Dominica, Montserrat.

##### Chrysotus hirsutus

Taxon classificationAnimaliaDipteraDolichopodidae

Aldrich

830F9007-2D28-51DA-A850-21498D73966C

Chrysotus hirsutus Aldrich, 1896: 328.

###### Material examined.

**Dominica**: 4 ♂, 4 ♀, Cabrits National Park, East Cabrits Trail, 15.58564N, 61.47210W, Malaise, 30 May–7 June 2011, M.A. & L.L. Ivie. **Montserrat**: 19 ♂, 12 ♀, Woodlands, Riverside House, 10–12 January 2002, Malaise trap, Ivie, Marske, Puliafico; 3 ♂, 2 ♀, same as previous, 5–7 January 2002; 2 ♂, Cassava Ghaut, Beattie House, 14–21 January 2002, Malaise trap, A. Krakower; 1 ♂, same as previous, 18 March–4 April 2002; 1 ♀, same as previous, 21–30 June 2002, UV light, M.A. Ivie; 1 ♂, Hope Ghaut, 8–10 January 2002, yellow pan traps, K.A. Marske; 1 ♂, Bottomless Ghaut, 5 August 2005, yellow pan traps, V.G. Martinson; 2 ♂, trail to Fairy Walk, 15 August 2005, yellow pan traps, V.G. Martinson (MTEC, USNM). **St. Vincent**: Syntype ♂ (USNM).

###### Distribution.

Widespread in the New World tropics.

##### Chrysotus interfrons

Taxon classificationAnimaliaDipteraDolichopodidae

sp. nov .

08F5193D-A765-5E7C-917D-9B03CE71115D

http://zoobank.org/BA1E88D1-F0B5-4F72-B2DA-462C39498D26

Figs 24 , 25 , 26

###### Type material.

***Holotype***, ♂ labelled: “MONTSERRAT:/ Cedar Ghaut/ 04AUG2005, V. G./ Martinson, D. Hughley/ Yellow Pan Trap”; “HOLOTYPE/ ♂ *Chrysotus*/ *interfrons*/ Runyon [red label]” (USNM, type number USNMENT01350615). ***Paratypes*: Montserrat**: 1 ♂, Woodlands, Riverside House, 5–7 January 2002, Malaise trap, Ivie, Marske & Puliafico; 2 ♂, 1 ♀, Hope Ghaut, 8–10 January 2002, yellow pan trap, K.A. Marske; 1 ♂, Cassava Ghaut, 877 ft, 16°45.75'N, 62°12.47'W, fogging at dawn, K. Marske & J. Boatswain; 1 ♂, Beattie House, 21–30 June 2002, uv light, M.A. Ivie; 2 ♂, Fogarty Ghaut (Soldiers), 16°46.41'N, 62°12.44'W, 21 June 2017, J.B. Runyon; 1 ♂, Runaway Ghaut, 175 m, 16°45.43'N, 62°12.89'W, 23 June 2017, J.B. Runyon; 1 ♂, Fairy Walk River, 260 m, 16°45.162'N, 62°10.854'W, 26 June 2017, J.B. Runyon (MTEC, USNM).

###### Description.

**Figure 24.**
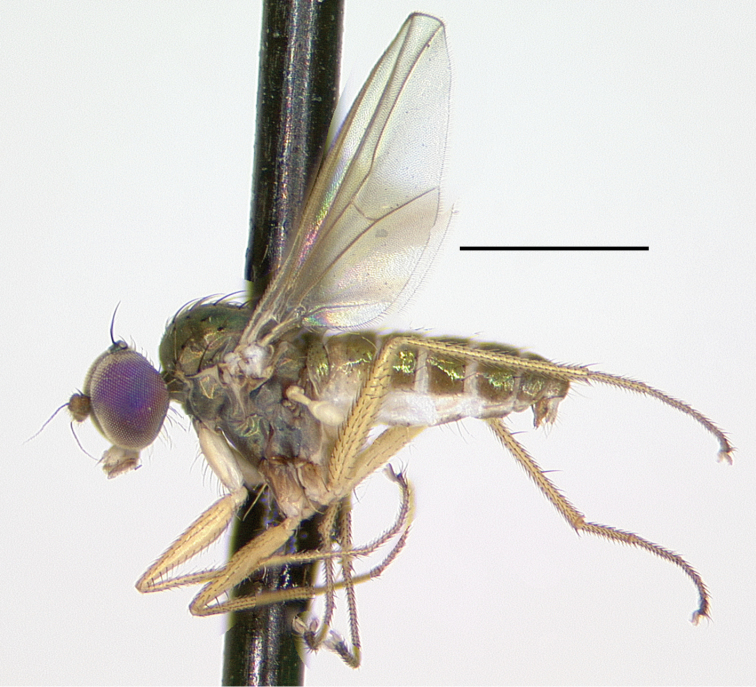
*Chrysotusinterfrons* sp. nov. habitus of holotype male, left lateral. Scale bar: 1.0 mm.

**Figure 25.**
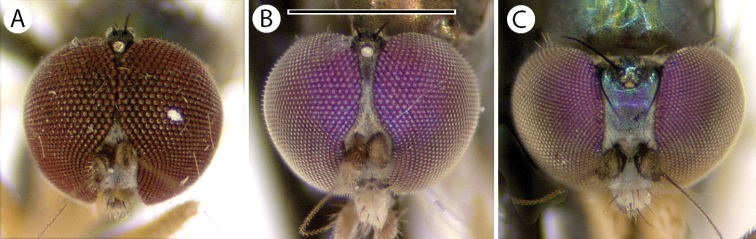
Heads of males, anterodorsal views **A***Chrysotusflavipes* (Aldrich) (Dominica) **B***Chrysotusinterfrons* sp. nov. (Montserrat) **C***Chrysotusparvulus* (Aldrich) (Montserrat). Note differences in width of frons and enlargement of ommatidia. Scale bar: 0.5 mm.

**Figure 26.**
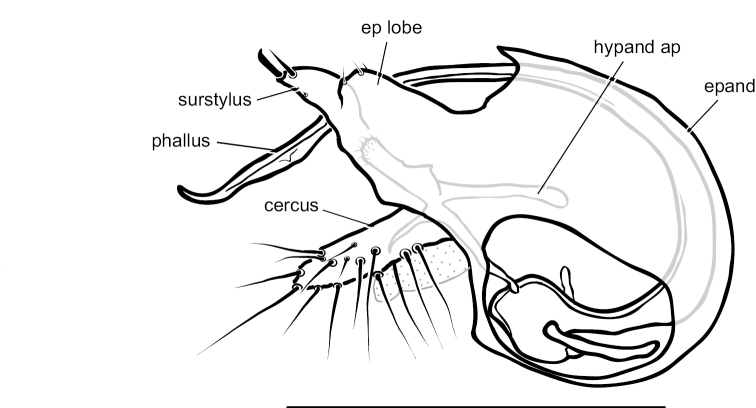
*Chrysotusinterfrons* sp. nov. male terminalia, left lateral. Abbreviations: epand-epandrium; ep lobe-epandrial lobe; hypand ap-hypandrial apodeme. Scale bar: 0.25 mm.

**Male** (Fig. 24). Body length 2.7–2.8 mm, wing length 2.2–2.3 × width 0.9–1.1 mm. ***Head***: Face recessed, ca. 1.5 × as high as wide, nearly rectangular but very slightly narrowed below middle, ca. 3 × as wide as frons, metallic green cuticle almost completely obscured by yellowish white pruinosity. Frons (Fig. 25B) narrowed above with narrowest width subequal to width of anterior ocellus, dark metallic green mostly obscured by white to yellowish white pruinosity. Palpus narrowly oval, pale yellow with a few small black setae. Proboscis dark yellow, with very fine brown hairs along margin. Antenna brown-yellow; first flagellomere small, truncated-triangular, two-thirds as long as high; arista-like stylus arising from median-apical sinus. Postocular setae pale yellow, uppermost four or five becoming brown; ventral postcranium with ca. four pale yellow setae per side that are longer than postocular setae. ***Thorax***: Scutum and scutellum dark metallic green with weak copper and blue reflections and sparse light brown pruinosity that is denser on anterior and lateral slopes; setae on scutum light brown to black; seven pairs of biseriate acrostichal setae; six pairs of dorsocentral setae, anterior-most pair small; scutellum with one pair of large marginal setae and one pair of very small setae just lateral to larger setae. Pleuron dark brown with metallic blue reflections and moderately dense gray pruinosity; 1–3 yellow setae on lower proepisternum; upper proepisternum bare. ***Legs***: Yellow, except most of coxa II, basal half or more of coxa III and 5^th^ segment of tarsi brown. Anterior surface of coxa I with scattered, rather long yellow to brown setae; coxa II with scattered yellow to brown setae on anterior surface and large ad yellowish seta near 1/2; coxa III with large yellowish lateral seta near base. Femora I and II with pv row of brown setae (length < width of femur), those distally longest; femur III anteriorly and posteriorly with one or two erect yellow setae near base. Tibia I without distinctive setae but setae along ventral surface slightly longer (length subequal to width of tibia), finer, and usually paler than those on dorsal surface; tibia II with very small brown to black ad seta near 1/5 (length < width of tibia), sometimes a trace of a pd seta near 1/2, and 2 larger brown ventral setae at apex; tibia III with small ad seta near 1/5, usually larger pd seta near 2/5, 3/5, near tip, and very small pd seta near 1/6. Tarsi with pulvilli enlarged, on tarsus I slightly larger than tarsomere 5, less enlarged on tarsi II and III; tarsal claws absent on all legs. Ratios of tibia:tarsomeres: leg I: 36–22–10–5–4–4; leg II: 42–24–11–7–3–4; leg III: 50–16–13–8–5–4. ***Wing***: Hyaline, broadly elliptical with well-developed anal lobe, veins brown. R_2+3_ straight, slightly and evenly diverging from R_4+5_. R_4+5_ and M_1_ nearly parallel beyond crossvein dm-cu. Crossvein dm-cu slightly less than half as long as last part of CuA_1_. Calypter yellow with yellow to light brown setae. Halter knob and stem light yellow. ***Abdomen***: Cylindrical, metallic green with bronze reflections, sides of tergite II and basal sternites yellow. Setae of tergites brown to black, sternites with longer usually yellow setae. Tergite VI bare except one distolateral seta at lower margin. Sternite VIII with ca. eight brownish setae of various sizes, the largest two or three only slightly larger than setae along margins of tergites. Hypopygium (Fig. 26) small, brown, positioned in ventral notch at tip of abdomen. Hypopygial foramen lateral but positioned relatively far posteriorly and near dorsal edge. Epandrium brown, ventrally with small spine-like projection where phallus emerges; with broad ventroapical lobe that is subquadrate apically with ca. three small setae. Surstylus elongate, rounded ventrally, shining brown, with strong apical cylindrical seta with rounded tip that is subtended by a smaller seta. Cercus digitiform with slightly pointed apex, brownish yellow, with numerous stiff yellow to brown setae especially along basal half of dorsal margin. Phallus narrow with apex round; sheath of phallus membranous, slightly expanded subapically with a small tooth in membrane. Postgonites rounded apically with some microtrichia. Hypandrial apodemes rather long and narrow.

**Female.** Body length 2.8 mm, wing length 2.0 × width 0.9 mm. Similar to male, but face and frons with blue-violet reflections and yellow-brown pruinosity that is denser along eyes; frons as wide as face; clypeus distinct and forming lower one-third of face; palpus broader with a few more setae; antenna slightly shorter and more rounded distally; femora I and II with pv row of setae smaller and indistinct basally; femur III without erect setae near base; pulvilli not enlarged; wing tinged with brown; tarsal claws present on all legs.

###### Etymology.

This species is named for the relative width of the frons in males which is intermediate to males of the closely related species *Chrysotusflavipes* (Aldrich) and *Chrysotusparvulus* (Aldrich) (Fig. 25).

###### Distribution.

Montserrat.

###### Remarks.

*Chrysotusinterfrons* forms a closely related group with two other West Indian species that have to date been treated in the genus *Diaphorus*. However, these species do not fit in the current definition of *Diaphorus* proposed by Robinson and Vockeroth (1981) since they lack setae on the upper proepisternum, black calypteral setae, a completely bare tergite VI, and four to eight long strong setae on sternite VIII (see discussion in Capellari and Amorim 2010). *Diaphorusparvulus* Aldrich was transferred to *Chrysotus* by Becker (1922, page 170), an act that has been largely overlooked. *Diaphorusflavipes* Aldrich is herein moved to *Chrysotus*: *Chrysotusflavipes* (Aldrich) comb. nov. *Chrysotusmundus* (Loew) is very similar to *Chrysotusflavipes* and the two are possibly conspecific.

*Chrysotusinterfrons* is most easily separated from West Indian members of this group by the narrow frons (Fig. 25); additional characters distinguishing these related species are given in Table 3. *Chrysotusflavipes* and *C.parvulus* are widespread in the Lesser Antilles, and both occur on Dominica (Robinson 1975), Grenada (Aldrich 1902), and St. Vincent (Aldrich 1896) but *C.flavipes* is absent on Monserrat.

**Table 3.** Characters distinguishing males of the closely related *Chrysotusflavipes* (Aldrich), *C.interfrons* sp. nov., and *C.parvulus* (Aldrich).

Character	*Chrysotusflavipes*	*C.interfrons*	*C.parvulus*
Frons (Fig. 25)	obliterated by contiguous eyes	narrow	as wide as face
Face shape	height subequal to width	height ca. 1.5 × width	height ca. 1.5 × width
Ommatidia	dorsal facets greatly enlarged	dorsal facets slightly enlarged	dorsal facets not enlarged
Size *ad* seta on tibia II	small (length < width of tibia)	small (length < width of tibia)	large (length > width of tibia)

##### Chrysotus integer

Taxon classificationAnimaliaDipteraDolichopodidae

Robinson

2A7615C6-7239-5738-9A5D-59D34C4EB807

Chrysotus integer Robinson, 1975: 75.

###### Material examined.

**Dominica: *Holotype*** ♂, Clarke Hall, 11–20 January 1965, Malaise trap, W.W. Wirth (USNM). **Montserrat**: 3 ♂, Woodlands, Riverside House, 10–12 January 2002, Malaise trap, Ivie, Marske, Puliafico; 2 ♂, same as previous, 5–7 January 2002; 1 ♂, Cassava Ghaut, Beattie House, 21 January–5 February 2002, Malaise trap, A. Krakower; 1 ♂, same as previous, 6–12 June 2002, UV light; 1 ♂, 1 ♀, Bottomless Ghaut, 5 August 2005, yellow pan traps, V.G. Martinson (MTEC, USNM).

###### Distribution.

Dominica, Grenada, and Montserrat.

##### Chrysotus lamellicaudatus

Taxon classificationAnimaliaDipteraDolichopodidae

Robinson

C2CFDD73-17BE-589F-AB83-F6699E16AA31

Chrysotus lamellicaudatus Robinson, 1975: 87.

###### Material examined.

**Dominica: *Holotype*** ♂, South Chiltern Estate, 2 February 1965, W.W. Wirth (USNM). **Montserrat**: 1 ♂, Woodlands, Riverside House, 5–7 January 2002, Malaise trap, Ivie, Marske, Puliafico; 1 ♂, same as previous, 22 July 2005; yellow pan traps, V.G. Martinson; 3 ♂, 4 ♀, Cassava Ghaut, 24 July 2005, yellow pan traps, V.G. Martinson; 2 ♂, Bottomless Ghaut, 5 August 2005, yellow pan traps, V.G. Martinson; 2 ♂, Bottomless Ghaut trail to Big River, 14 August 2005, yellow pan traps, V.G. Martinson; 3 ♂, Hope Ghaut, 8–10 January 2002, yellow pan traps, K.A. Marske; 1 ♂, Sweetwater Ghaut, 1 August 2005, yellow pan traps, V.G. Martinson; 1 ♂, Killiekranke, 3 August 2005, yellow pan traps, V.G. Martinson; 5 ♂, Hope Ghaut, 300 m, 16°45.108'N, 62°12.695'W, 20 June 2017, J.B. Runyon; 3 ♂, Fogarty Ghaut (Soldiers), 16°46.41'N, 62°12.44'W, 21 June 2017, J.B. Runyon; 1 ♂, Runaway Ghaut, 175 m, 16°45.43'N, 62°12.89'W, 23 June 2017, J.B. Runyon; 1 ♂, Corbett Spring, 300 m, 16°45.012'N, 62°11.184'W, 26 June 2017, J.B. Runyon; 1 ♂, Fairy Walk River, 260 m, 16°45.162'N, 62°10.854'W, 26 June 2017, J.B. Runyon; 1 ♂, ghaut above Montserrat Volcano Observatory, 330 m, 16°45.130'N, 62°12.487'W, 27 June 2017, J.B. Runyon; 3 ♂, 2 ♀, Big River, 450 m, 16°45.690'N, 62°11.174'W, 28 June 2017, J.B. Runyon; 1 ♂, Bottomless Ghaut, 400 m, 16°45.994'N, 62°11.497'W, 28 June 2017, J.B. Runyon (MTEC, USNM).

###### Distribution.

Dominica, Montserrat.

##### Chrysotus mediocaudatus

Taxon classificationAnimaliaDipteraDolichopodidae

Robinson

C5D704E4-A795-5437-B4A7-D938365DCFD9

Chrysotus mediocaudatus Robinson, 1975: 87.

###### Material examined.

**Dominica: *Holotype*** ♂, Fond Figues River, 9 February 1965, W.W. Wirth (USNM). **Montserrat**: 3 ♂, ghaut above Montserrat Volcano Observatory, 330 m, 16°45.130'N, 62°12.487'W, 27 June 2017, J.B. Runyon (MTEC, USNM).

###### Distribution.

Dominica, Montserrat.

##### Chrysotus microtatus

Taxon classificationAnimaliaDipteraDolichopodidae

Meuffels & Grootaert

0C27A5B4-5A2E-5D39-A70C-6C0B390F2AE6

Chrysotus minimus Robinson, 1975: 82; preoccupied by Chrysotusminimus (Meigen, 1830).
Chrysotus microtatus Meuffels & Grootaert, 1999: 291; new name for Chrysotusminimus Robinson.

###### Material examined.

**Dominica: *Holotype*** ♂, Fond Figues River, rain forest, 3 February 1965, W.W. Wirth (USNM). **Montserrat**: 10 ♂, 7 ♀, Bottomless Ghaut, 5 August 2005, yellow pan traps, V.G. Martinson; 3 ♂, same as previous, 14 August 2005; 19 ♂, 21 ♀, Bottomless Ghaut trail to Big River, 14 August 2005, yellow pan traps, V.G. Martinson; 16 ♂, 4 ♀, Big River, 5 August 2005, yellow pan traps, V.G. Martinson; 2 ♂, 4 ♀, Big River, 450 m, 16°45.690'N, 62°11.174'W, 28 June 2017, J.B. Runyon; 1 ♂, Bottomless Ghaut, 400 m, 16°45.994'N, 62°11.497'W, 28 June 2017, J.B. Runyon (MTEC, USNM).

###### Distribution.

Dominica, Montserrat.

###### Remarks.

Adults of *Chrysotusmicrotatus* were found on Montserrat only in the deepest ghauts.

##### Chrysotus montserratensis sp. nov.

Taxon classificationAnimaliaDipteraDolichopodidae

1372713C-B08D-56DB-A2B0-1DE67D951547

http://zoobank.org/BF0C4941-5632-43FB-A438-2C1599991A6E

Figs 27 , 28 , 29

###### Type material.

***Holotype***, ♂ labelled: “WEST INDIES: MONTSERRAT/ Big River 450 m/ 16°45.690'N, 62°11.174'W/ 28 JUNE 2017, JB Runyon”; “HOLOTYPE/ ♂ *Chrysotus* / *montserratensis* / Runyon [red label]” (USNM, type number USNMENT01350614). ***Paratypes*: Montserrat**: 3 ♀, same data as holotype; 2 ♀, Big River, 5 August 2005, yellow pans, V.G. Martinson; 2 ♀, Jack Boy Hill (top), 480 m, 16°45.797'N, 62°10.886'W, 25 June 2017, J.B. Runyon; 1 ♀, ghaut above Montserrat Volcano Observatory, 330 m, 16°45.130'N, 62°12.487'W, 27 June 2017, J.B. Runyon; 1 ♂, Bottomless Ghaut, 400 m, 16°45.994'N, 62°11.497'W, 28 June 2017, J.B. Runyon; 1 ♀, Katy Hill (top), 730 m, 16°45.731'N, 62°11.646'W, 28 June 2017, J.B. Runyon (MTEC, USNM).

###### Description.

**Figure 27.**
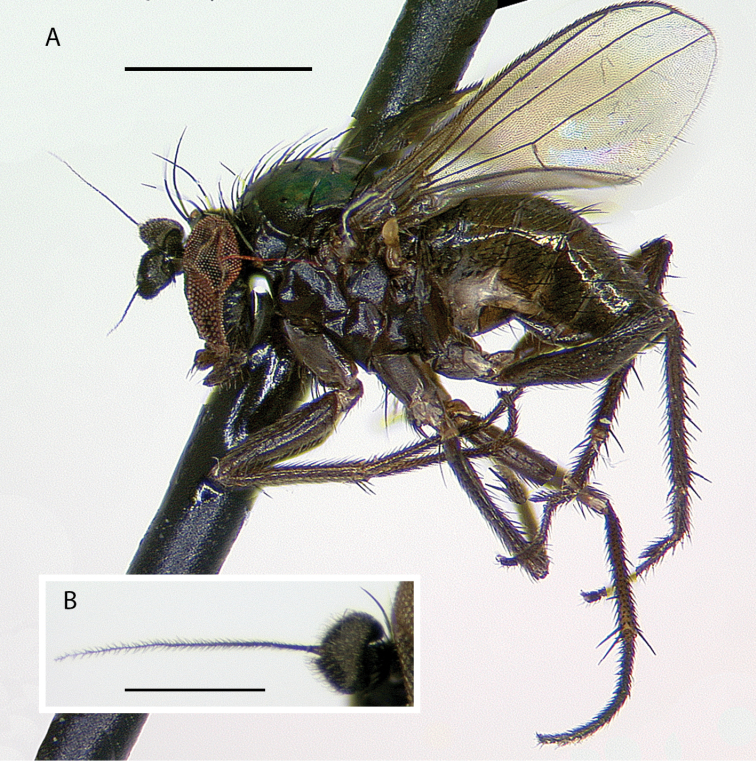
*Chrysotusmontserratensis* sp. nov. **A** habitus of holotype male, left lateral **B** female antenna, left lateral. Scale bars: 1.0 mm (**A**), 0.5 mm (**B**).

**Figure 28.**
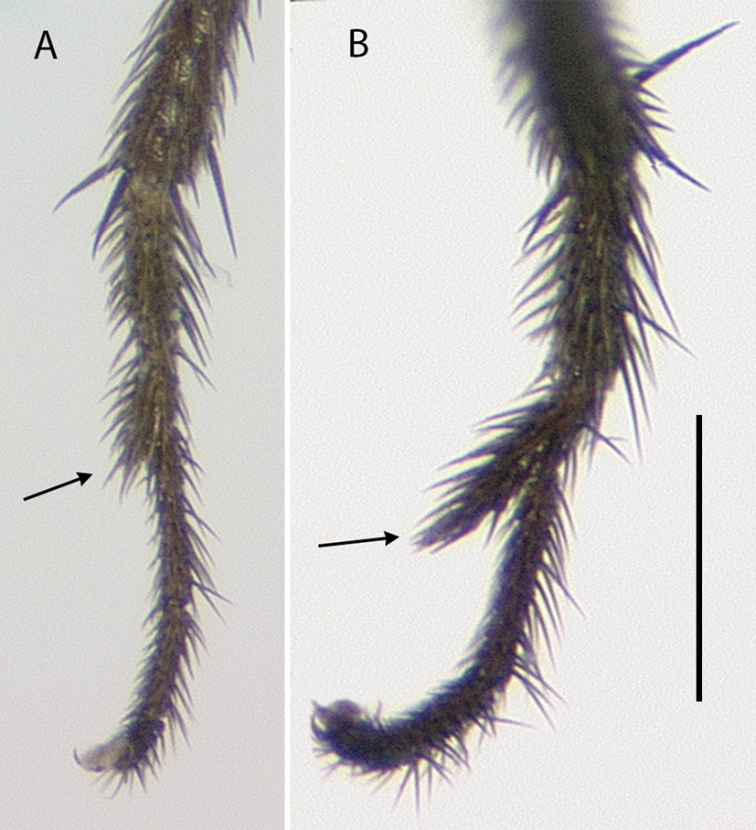
Tarsus III of males, posterior views **A***Chrysotusexcisus* Aldrich (Dominica) **B***Chrysotusmontserratensis* sp. nov. Arrows indicate spur-like projection on tarsus III(2). Scale bar: 0.5 mm.

**Figure 29.**
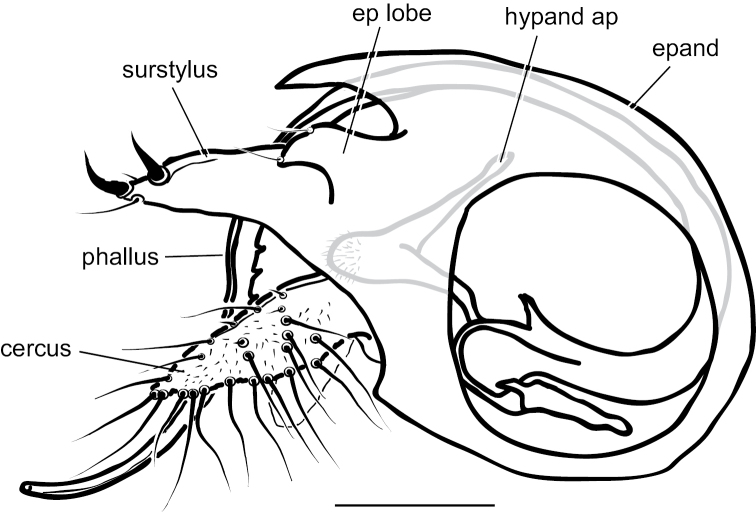
*Chrysotusmontserratensis* sp. nov. male terminalia, left lateral. Abbreviations: epand-epandrium; ep lobe-epandrial lobe; hypand ap-hypandrial apodeme. Scale bar: 0.1 mm.

**Male** (Fig. 27A). Body length 2.9–3.0 mm, wing length 2.4–2.5 × width 0.9–1.0 mm. ***Head***: Eyes essentially contiguous below; face dark metallic green-blue obscured by light brown pruinosity, lower half of face very narrow (subequal in width to one ommatidium) and nearly parallel-sided, upper face narrow triangular. Frons metallic green-blue with brown pruinosity. Palpus brown, subquadrate with rounded corners, with ca. four rather large black setae (longest subequal to width of palpus). Proboscis dark brown, somewhat enlarged and projecting anteriorly, with brown hairs along margin. Antenna (Fig. 27A) black; first flagellomere large, triangular-ovate to crescent-shaped, base extending above and overlapping pedicel; arista-like stylus subapical, inserted in shallow notch. Postocular setae brown to black. ***Thorax***: Scutum and scutellum dark metallic green with slight blue reflections and sparse light brown pruinosity; setae on scutum black; six pairs of rather large irregularly biseriate acrostichal setae; six pairs of dorsocentral setae; scutellum with one pair of large marginal setae and one pair of smaller lateral setae (ca. half length of larger setae). Pleuron dark brown to almost black with slight green-blue reflections, obscured by gray pruinosity; with two brown to black setae on lower proepisternum. ***Legs***: Dark brown to black with black setae and hairs. Coxa I with rather long coarse anterior setae becoming larger distally; coxa II apically with two small brown spines composed of fused setae. Trochanter II with large av seta. Femur I with slightly longer pv setae full-length, those at very base erect, those near tip larger. Femur II with slightly longer setae av and pv, with subapical row of four to five posterior setae; femur III with a few larger preapical av, pv, and anterior setae. Tibia I with very small ad seta near 1/4 and larger pd seta at apex, with ventral setae slightly longer; tibia II with large ad seta near 1/4 preceded by very small seta, a smaller ad seta near 1/2, ventrally with a seta near 1/3, 1/2, and 3/5 which increase in size distally, and four large apical setae; tibia III with 3–5 ad setae the largest near 1/5, 2/5, and just beyond 1/2, four or five pd setae with largest near 1/3, 2/5, 3/5, four apical or subapical setae, ventral surface with slightly longer setae. Ventral surface of tarsomeres I(1, 2) with longer setae (longest slightly wider than tarsomere); tarsus III (Fig. 28B) with dense short brushy setae; tarsomere III(1) a little broadened with longest setae anteriorly; tarsomere III(2) prolonged posteriorly into a spur overlapping ca. half of tarsomere III(3), this spur covered ventrally with short dense setae; tarsomeres III(3, 4) with slightly longer setae anteriorly and dorsally. Pulvilli not enlarged, all legs with two claws. Ratios of tibia:tarsomeres: leg I: 38–22–9–8–6–4; leg II: 40–20–8–7–4–3; leg III: 50–14–6 (12 including spur)–11–8–4. ***Wing***: Hyaline, elliptical, with brown veins. R_2+3_ straight. R_4+5_ and M_1_ nearly parallel in apical half of wing, both curving slightly backward apically. Last part of CuA_1_ ca. 2.5 × as long as crossvein dm-cu. Calypter dark brown with black setae. Halter knob and stem yellow. ***Abdomen***: Cylindrical, rather broad, gradually tapering, with hairs and setae black. Tergites and sternites dark metallic greenish, nearly black; tergite VI with numerous small setae and larger marginal setae. Sternite VIII with small setae, covering hypopygial foramen. Hypopygium (Fig. 29) small, blackish, positioned in ventral notch at tip of abdomen. Epandrium black, with small ventroapical lobe bearing two small setae. Surstylus elongate, shining dark brown, with strong subapical spine and spine at apex, and two small hairs near apex. Cercus elongate triangular, brown, with numerous stiff brown setae especially along dorsal margin; more sclerotized narrowly along ventral edge. Phallus simple, narrow, with apex round; sheath of phallus broadened dorsally near and beyond emergence from epandrium, with three or four small teeth along dorsal margin ca. midway between epandrium and tip of phallus. Postgonites rounded apically with some microtrichia. Hypandrial apodemes long and narrow.

**Female.** Body length 3.1–3.5 mm, wing length 2.6–3.1 × width 0.9–1.4 mm. Similar to male, but face wide, narrowest part ca. two-thirds width of frons at ocellus; dark metallic green-blue obscured by very sparse light brown pruinosity; clypeus distinct, bulging at suture, slightly widened; palpus broader, more rounded apically, with more setae; first flagellomere (Fig. 27B) smaller, length two-thirds height, distinctly crescent-shaped in medial view, apical notch smaller; scutum with strong metallic green-blue-violet reflections; coxa II without apical spines; tarsomere II(1) with less distinct ventral setae; tarsus III without dense brushy setae; tarsus III(2) without spur.

###### Etymology.

This species is named for the island of Montserrat.

###### Distribution.

Montserrat.

###### Remarks.

*Chrysotusmontserratensis* is related to *C.excisus* Aldrich (Dominica, Mexico, St. Vincent) and *C.pseudexcisus* (Dominica) being most closely related to *C.excisus* which shares tarsomere III(2) prolonged posteriorly in a spur overlapping base of tarsomere III(3). *Chrysotusmontserratensis* differs in having a larger spur on tarsomere III(2) (Fig. 28), larger body size, and a yellow halter knob.

##### Chrysotus orichalceus

Taxon classificationAnimaliaDipteraDolichopodidae

Gosseries

805F5F04-EB0E-5C83-AE68-4957FDEBA67E

Chrysotus niger Aldrich, 1896: 327; preoccupied by Chrysotusniger Loew, 1869.
Chrysotus orichalceus Gosseries, 1988: 305; new name for Chrysotusniger Aldrich.

###### Material examined.

**Dominica**: ♂, Clarke Hall, 11–20 January 1965, Malaise trap, W.W. Wirth (USNM). **Montserrat**: 22 ♂, 6 ♀, Woodlands, Riverside House, 10–12 January 2002, Malaise trap, Ivie, Marske, Puliafico; 15 ♂, 6 ♀, same as previous, 5–7 January 2002; 2 ♂, 2 ♀, Cassava Ghaut, Beattie House, 4–23 March 2002, Malaise trap, A. Krakower; 1 ♂, same as previous, 18 March–4 April 2002; 2 ♂, Hope Ghaut, 8–10 January 2002, yellow pan traps, K.A. Marske; 1 ♂, 2 ♀, Jack Boy Hill, 28 July 2005, yellow pan traps, V.G. Martinson; 1 ♂, Hope Ghaut, 23 July 2005, yellow pan traps, V.G. Martinson; 1 ♂, Sweetwater Ghaut, 1 August 2005, yellow pan traps, V.G. Martinson; 1 ♂, Runaway Ghaut, roadside springs, 150 m, 16°45.449'N, 62°13.011'W, 22 June 2017, J.B. Runyon (MTEC, USNM). **St. Vincent**: Syntypes 2 ♂, 2 ♀, May (USNM).

###### Distribution.

Dominica, Montserrat, and St. Vincent.

##### Chrysotus parvulus

Taxon classificationAnimaliaDipteraDolichopodidae

(Aldrich)

8798103A-0C84-5352-B7F4-0ECF4738FD80

Diaphorus parvulus Aldrich, 1896: 321.
Chrysotus longipes Van Duzee, 1927: 1.

###### Material examined.

**Dominica**: 3 ♂, 23 January–17 February 1964, H. Robinson; 1 ♂, 1 ♀, St. Mark Parish, 4 km N Soufriere, 75 m, 17–19 March 2003, E. Bentson, G. Carner; 13 ♂, Cabrits National Park, East Cabrits Trail, 15.58564N, 61.47210W, Malaise, 30 May–7 June 2011, M.A. & L.L. Ivie. **Montserrat**: 1 ♂, 3 ♀, Underwood Ghaut, canopy fogging at dawn, 23 May 2002, K. Marske & J. Boatswain; 1 ♂, 1 ♀, Woodlands, Riverside House, 22 July 2005, yellow pan traps, V.G. Martinson; 1 ♂, 4 ♀, Bottomless Ghaut, 5 August 2005, yellow pan trap, V.G. Martinson; 6 ♂, 4 ♀, same as previous, 14 August 2005; 2 ♂, 4 ♀, Killiekranke, 3 August 2005, yellow pan trap, V.G. Martinson; 9 ♂, 1 ♀, Big River, 5 August 2005, yellow pan traps, V.G. Martinson; 4 ♂, 6 ♀, Bottomless Ghaut trail to Big River, 14 August 2005, yellow pan traps, V.G. Martinson; 1 ♂, Cassava Ghaut, 24 June 2005, yellow pan traps, V.G. Martinson; 2 ♂, 3 ♀, Fogarty Ghaut (Soldiers), 16°46.41'N, 62°12.44'W, 21 June 2017, J.B. Runyon; 2 ♂, 3 ♀, Jack Boy Hill (top), 480 m, 16°45.797'N, 62°10.886'W, 25 June 2017, J.B. Runyon; 1 ♀, Big River, 450 m, 16°45.690'N, 62°11.174'W, 28 June 2017, J.B. Runyon; 1 ♀, Bottomless Ghaut, 400 m, 16°45.994'N, 62°11.497'W, 28 June 2017, J.B. Runyon; 1 ♂, 1 ♀, Katy Hill (top), 730 m, 16°45.731'N, 62°11.646'W, 28 June 2017, J.B. Runyon; 1 ♂, ghaut above Montserrat Volcano Observatory, 330 m, 16°45.130'N, 62°12.487'W, 27 June 2017, J.B. Runyon (MTEC, USNM).

###### Distribution.

Lesser Antilles and Puerto Rico.

###### Remarks.

This species was moved from *Diaphorus* to *Chrysotus* by Becker (1922, page 170), an act that has gone largely unnoticed. Becker’s move was correct because this species does not fit in the current definition of the genus *Diaphorus* (see Remarks for *C.interfrons* sp. nov.). In particular it lacks setae on the upper proepisternum, black calypteral setae, a completely bare tergite 6, and four to eight long strong setae on sternite 8. The species fits in the broadly defined *Chrysotus* following Pollet et al. (2004), which is likely paraphyletic and might need subdivision as diaphorine phylogeny is elucidated (Capellari and Amorim 2010). This necessitates a new replacement name for the Nearctic *Chysotusparvulus* Van Duzee, 1924, as:

##### Chrysotus milvadu nom. nov.

Taxon classificationAnimaliaDipteraDolichopodidae

DD7340B2-4064-5E17-9578-24B508F34090

Chysotus parvulus Van Duzee, 1924b: 25; preoccupied by Chrysotusparvulus (Aldrich, 1896).

###### Note.

This honorary new name is derived from combining the first two or three letters each of Millard Van Duzee and is treated here as a noun in apposition.

##### Chrysotus proximus

Taxon classificationAnimaliaDipteraDolichopodidae

Aldrich

6AC28BC2-1F82-55DB-880E-A0B563D3DB37

Chrysotus proximus Aldrich, 1896: 326.

###### Material examined.

**Montserrat**: 2 ♂, Cassava Ghaut, Beattie House, 14–21 January 2002, Malaise trap, A. Krakower; 1 ♂, Hope Ghaut, 8–10 January 2002, yellow pan traps, K.A. Marske; 1 ♂, Hope Ghaut, 300 m, 16°45.108'N, 62°12.695'W, 20 June 2017, J.B. Runyon; 1 ♀, Runaway Ghaut, 175 m, 16°45.43'N, 62°12.89'W, 23 June 2017, J.B. Runyon; 3 ♂, 9 ♀, Jack Boy Hill (top), 480 m, 16°45.797'N, 62°10.886'W, 25 June 2017, J.B. Runyon; 7 ♂, 2 ♀, ghaut above Montserrat Volcano Observatory, 330 m, 16°45.130'N, 62°12.487'W, 27 June 2017, J.B. Runyon (MTEC, USNM). **St. Vincent**: Syntypes 1 ♂, 1 ♀ (USNM).

###### Distribution.

Lesser Antilles (Dominica, Grenada, Montserrat, and St. Vincent).

##### Chrysotus pseudoniger

Taxon classificationAnimaliaDipteraDolichopodidae

Robinson

988A7944-CB55-5B27-8B88-D2E1F363F83D

Chrysotus pseudoniger Robinson, 1975: 89.

###### Material examined.

**Dominica: *Holotype*** ♂, Clarke Hall, cocoa trail, 18 January 1965, W.W. Wirth (USNM). **Montserrat**: 1 ♂, Hope Ghaut, 23 July 2005, yellow pan traps, V.G. Martinson; 4 ♂, Big River, 5 August 2005, yellow pan traps, V.G. Martinson; 2 ♂, 1 ♀, Bottomless Ghaut, 5 August 2005, yellow pan traps, V.G. Martinson; 4 ♂, 1 ♀, Fairy Walk, 9 August 2005, yellow pan traps, V.G. Martinson; 1 ♂, Bottomless Ghaut trail to Big River, 14 August 2005, yellow pan traps, V.G. Martinson; 2 ♂, Hope Ghaut, 300 m, 16°45.108'N, 62°12.695'W, 20 June 2017, J.B. Runyon (MTEC, USNM).

###### Distribution.

Dominica, Montserrat.

##### Chrysotus spectabilis

Taxon classificationAnimaliaDipteraDolichopodidae

(Loew)

BAE1640B-52CF-5ABF-B864-6334E8BDCDF4

Diaphorus spectabilis Loew, 1861: 57.
Diaphorus exunguis Thomson, 1869: 506.
Diaphorus approximatus Aldrich, 1896: 321.
Dubious spectabilis (Loew) [unwarranted combination by Wei 2012: 611].

###### Material examined.

**Dominica**: 10 ♂, 1 ♀, Springfield Estate, yellow pans, 1–3 June 2011, M.A. & L.L. Ivie. **Montserrat**: 22 ♂, 24 ♀, Woodlands, Riverside House, 10–12 January 2002, Malaise trap, Ivie, Marske & Puliafico; 1 ♂, same as previous, 5–7 January 2002; 3 ♂, Hope Ghaut, 8–10 January 2002, yellow pan traps, K.A. Marske; 5 ♂, Cassava Ghaut, Beattie House, 14–21 January 2002, Malaise trap, A. Krakower; 1 ♂, same as previous, 5–15 February 2002; 1 ♂, same as previous, 23 March–8 April 2002; 1 ♂, same as previous, 8–17 April 2002; 1 ♂, same as previous, 17 April–01 May 2002; 3 ♂, 1 ♀, same as previous, 13–14 January 2002, blacklight, M.A. Ivie & K.A. Marske; 1 ♂, same as previous, canopy fogging at dawn, 21 May 2002, K. Marske & J. Boatswain; 3 ♂, same as previous, 24 June 2005, yellow pan traps, V.G. Martinson; 1 ♂, Big River, 5 August 2005, yellow pan traps, V.G. Martinson; 2 ♂, Bottomless Ghaut, 5 August 2005, yellow pan traps, V.G. Martinson; 3 ♂, 1 ♀, Jack Boy Hill, 28 July 2005, yellow pan traps, V.G. Martinson; 2 ♂, Cedar Ghaut, 4 August 2005, yellow pan traps, V.G. Martinson (MTEC, USNM).

###### Distribution.

Eastern North America south to Argentina.

###### Remarks.

Capellari and Amorim (2010) re-described and illustrated this species. Wei (2012) proposed placement of *C.spectabilis* in the new genus *Dubius* Wei, but I concur with Capellari and Amorim (2014) that this is unjustified.

##### Chrysotus spinipes

Taxon classificationAnimaliaDipteraDolichopodidae

Van Duzee

B71A55E9-A4BC-531A-9922-853F057E9CB0

Chrysotus spinipes Van Duzee, 1924b: 19.

###### Material examined.

**Cuba: *Holotype*** ♂, Havana, Baker (CAS). **Dominica**: 1 ♂, Clarke Hall, 21–28 February 1965, light trap, W.W. Wirth (USNM). **Montserrat**: 3 ♂, Woodlands, Riverside House, 10–12 January 2002, Malaise trap, Ivie, Marske & Puliafico (MTEC, USNM).

###### Distribution.

Cuba, Dominica, Montserrat.

##### Chrysotus xiphostoma

Taxon classificationAnimaliaDipteraDolichopodidae

Robinson

C9567F10-582F-5753-87A6-67A6A0F76774

Chrysotus xiphostoma Robinson, 1975: 84.

###### Material examined.

**Dominica: *Holotype*** ♂, Clarke Hall, 21–23 January 1965, light trap, W.W. Wirth (USNM). **Montserrat**: 37 ♂, 18 ♀, Woodlands, Riverside House, 10–12 January 2002, Malaise trap, Ivie, Marske & Puliafico; 10 ♂, 6 ♀, same as previous, 5–7 January 2002; 2 ♂, Hope Ghaut, 8–10 January 2002, yellow pan traps, K.A. Marske; 2 ♂, Bottomless Ghaut, 5 August 2005, yellow pan traps, V.G. Martinson; 4 ♂, Bottomless Ghaut trail to Big River, 14 August 2005, yellow pan traps, V.G. Martinson; 1 ♂, Big River, 5 August 2005, yellow pan traps, V.G. Martinson; 2 ♂, Sweetwater Ghaut, 1 August 2005, yellow pan traps, V.G. Martinson; 1 ♂, Cedar Ghaut, 4 August 2005, yellow pan traps, V.G. Martinson; 1 ♂, Hope Ghaut, 300 m, 16°45.108'N, 62°12.695'W, 20 June 2017, J.B. Runyon; 1 ♀, Runaway Ghaut, roadside springs, 150 m, 16°45.449'N, 62°13.011'W, 22 June 2017, J.B. Runyon (MTEC, USNM).

###### Distribution.

Lesser Antilles (Dominica, Montserrat, Nevis, Saint Kitts, Saint Lucia) (Runyon and Capellari 2018).

###### Remarks.

*Chrysotusxiphostoma* belongs to the *Chysotuslongipalpus* species group and was re-described and illustrated by Capellari (2015).

#### Genus *Diaphorus* Meigen

##### Key to the species of *Diaphorus* in Montserrat

**Table:** 

1	Femora mostly brown with tips yellow; lower postocular setae black; knob of halter brownish	***D.contiguus* Aldrich**
–	Femora wholly yellow; lower postocular setae white; knob of halter yellow	***D.robinsoni* sp. nov .**

##### Diaphorus contiguus

Taxon classificationAnimaliaDipteraDolichopodidae

Aldrich

6F238983-62D6-5F54-ABA6-4E320565C24A

Diaphorus contiguus Aldrich, 1896: 323.

###### Material examined.

**Montserrat**: 1 ♂, Woodlands, Riverside House, 10–12 January 2002, Malaise trap, Ivie, Marske & Puliafico (MTEC). **St. Vincent**: Syntype 1 ♂ (USNM).

###### Distribution.

Southeastern USA, Bermuda, and the Lesser Antilles (Dominica, Montserrat, St. Vincent).

##### Diaphorus robinsoni

Taxon classificationAnimaliaDipteraDolichopodidae

sp. nov .

0EBCE809-1AF8-57EB-9DCC-F671EE5E4AB0

http://zoobank.org/AFBA73D2-A8AE-4402-AD99-583A54E4A27D

Figs 30 , 31

###### Type material.

***Holotype***, ♂ labelled: “DOMINICA: St. John Par./ Cabrits N.P. (malaise)/ East Cabrits Trail/ 15.58564N, 61.47210W/ 30MAY–07JUNE 2011/ M.A. & L.L. Ivie”; “HOLOTYPE/ ♂ *Diaphorus*/ *robinsoni*/ Runyon [red label]” (USNM, type number USNMENT01350616). ***Paratypes*: Dominica**: 3 ♂, 2 ♀, Clarke Hall, 2–21 March 1964, H. Robinson. **Montserrat**: 1 ♂, Cassava Ghaut, 877 ft, canopy fogging at dawn, 21 May 2002, K. Marske & J. Boatswain; 1 ♂, Hope Ghaut, 300 m, 16°45.108'N, 62°12.695'W, 20 June 2017, J.B. Runyon (MTEC).

**Figure 30.**
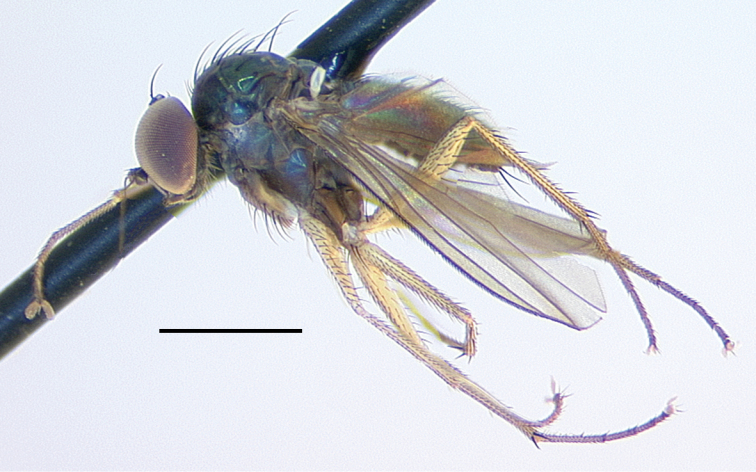
*Diaphorusrobinsoni* sp. nov. habitus of holotype male, left lateral. Scale bar: 1.0 mm.

**Figure 31.**
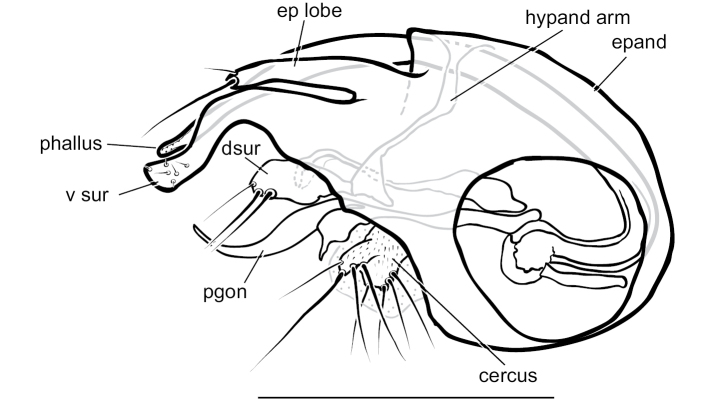
*Diaphorusrobinsoni* sp. nov. male terminalia, left lateral. Abbreviations: d sur-dorsal lobe of surstylus; epand-epandrium; ep lobe-epandrial lobe; hypand arm-hypandrial arm; pgon-postgonite; v sur-ventral lobe of surstylus. Scale bar: 0.25 mm.

**Description** (adapted from Robinson (1975), as *Diaphorusmundus*). **Male** (Fig. 30). Body length 2.8–3.3 mm, wing length 2.7–3.1 × width 1.2–1.5 mm. ***Head***: Eyes broadly contiguous above antenna, with ommatidia distinctly enlarged on dorsal half. Face distinctly recessed, as high as wide, metallic bluish with slight whitish pruinosity that is densest and brownish along eyes. Frons reduced to a narrow triangle immediately above antennae, covered with dense brown pruinosity. Palpus yellow with black setae, with one distinct large black seta at apex. Proboscis brown. Antenna brown; first flagellomere slightly yellowish basally, rather truncate, ca. 1.5 × as high as long, with small whitish hairs. Arista-like stylus apical, inserted at dorsal corner. Lower postocular setae multiseriate, white. ***Thorax***: Scutum and scutellum bright metallic green with some violet reflections and slight yellowish pruinosity; 5–7 pairs of black biseriate acrostichal setae; five pairs of black dorsocentral setae; scutellum with one pair of large marginal setae and one pair of small lateral setae. Pleuron slightly bluish with denser grayish yellow pruinosity; upper proepisternum with two small brownish setae, lower proepisternum with distinct black seta above coxa I. ***Legs***: Yellow except base of coxa I and all of coxae II and III brown. Hairs and setae black. Coxa I with small black setae on anterior surface and three or four large black setae spaced along distal two-thirds of lateral edge; coxa II with rather long, rather dense setae anteriorly and three larger ad setae; coxa III with large ad seta near base. Femur I with row of pv setae, basal-most seta in this series larger and erect, others progressively longer on distal two-thirds, with row of shorter av setae on basal two-thirds; femur II with both av and pv rows of short setae, pv series more distinct; femur III with longer erect setae in four ventral rows, one av and one pv seta near base stronger. Tibia I with only very small ad seta near 1/5; tibia II with rather large ad seta near 1/5 and smaller ad seta near 1/2, small pd seta near 1/5 and 1/2, distinct ventral seta near 3/4, four apical setae; tibia III with small ad seta near 1/5, larger pd seta near 1/5, 2/5, 3/5, and 4/5, four apical setae. Pulvilli much enlarged on leg I, moderately enlarged on leg II, and scarcely enlarged on leg III. Tarsal claws absent on legs I and II. Ratios of tibia:tarsomeres: leg I: 40–24–8–6–4–6; leg II: 48–28–12–8–4–3; leg III: 64–18–16–12–8–5. ***Wing***: Hyaline, oval, broad at base with well-developed anal lobe. Veins yellow-brown. R_1_ reaching 2/5 of wing length. R_2+3_ very slightly flexed, very slightly diverging from R_4+5_. M_1_ nearly straight and nearly parallel with R_4+5_ beyond crossvein. Crossvein dm-cu ca. two-thirds as long as last part of CuA_1_. Calypter yellow-brown with black setae. Halter knob yellow. ***Abdomen***: Wholly metallic green with strong coppery reflections. Hairs and setae black with some pale hairs on sternites; marginal setae of tergites and those laterally on tergite II 2–3 × as long as background setae. Tergite VI bare. Sternite VIII with four large, stout black setae projecting posteriorly from tip of preabdomen. Hypopygium (Fig. 31) small, dark brown, mostly concealed in ventral notch at tip of abdomen. Hypopygial foramen left basolateral, placed near dorsal edge of epandrium. Epandrium dark brown, with rather large finger-like ventroapical lobe bearing one large and one small seta at apex. Ventral lobe of surstylus elongate, rather sigmoid, with ca. six very small socketed setae near apex. Doral lobe of surstylus rather broadly rounded and with three setae. Cercus brown, small, bilobed with each lobe covered in minute hairs and bearing three or four brown setae. Phallus strongly arched, apex round and very slightly flared with several minute inward-facing teeth along lateral margin near apex. Hypandrial arms present, symmetrical, but not external to epandrium. Postgonites bilobed apically, ventral lobe large and hook-shaped, dorsal lobe smaller with pointed lobe immediately ventral to cercus.

**Female.** Body length 3.1 mm, wing length 3.1 × width 1.4 mm. Similar to male, but face 1.5 × as wide as high; clypeus distinct, forming lower two-fifths of face; front as wide as face with straight sides; face and front bluish with violet reflections; palpus broader, brownish, with apical setae distinct; femora without longer ventral setae; tibia II with small additional ad seta near base, second small ventral seta near 1/3, sometimes with an additional av or pd seta.

###### Etymology.

This species is named for Harold Robinson who collected and treated this species (as *D.mundus* Loew) on Dominica.

###### Distribution.

Dominica, Montserrat.

###### Remarks.

Robinson (1975) interpreted this species as *Diaphorusmundus* Loew which was transferred to the genus *Chrysotus* by Pollet et al. (2004) because it does not fit the definition of *Diaphorus* proposed by Robinson and Vockeroth (1981). *Chrysotusmundus* actually belongs to the group containing *C.interfrons* sp. nov. and *C.parvulus*. *Diaphorusrobinsoni* sp. nov. is a true *Diaphorus* and is most similar to *D.amazonicus* Parent (Brazil) but this species has tergites 2–3 yellow laterally (wholly green in *D.robinsoni*) and *Diaphorussubsejunctus* Loew (Cuba) which has the eyes not meeting above antenna and pale calypteral setae (and thus might not be a true *Diaphorus*).

#### Genus *Symbolia* Becker

##### Symbolia linearis

Taxon classificationAnimaliaDipteraDolichopodidae

(Aldrich)

5F1B29AA-B5C9-59D5-8949-075409EE0CEE

Anepsius linearis Aldrich, 1896: 317.
Sympycnus thoracicus Van Duzee, 1930b: 51.

###### Material examined.

**Dominica**: 1 ♀, Dleau Morne Laurent, 1 March 1964, H. Robinson (USNM). **Montserrat**: 1 ♀, Katy Hill (top), 730 m, 16°45.731'N, 62°11.646'W, 28 June 2017, J.B. Runyon (MTEC).

###### Distribution.

Lesser Antilles (Dominica, Grenada, Montserrat, and St. Vincent).

###### Remarks.

The single female collected on Montserrat was taken from a large leaf at the top of Katy Hill, the highest point sampled on the island.

### Subfamily Plagioneurinae

#### Genus *Plagioneurus* Loew

##### Plagioneurus univittatus

Taxon classificationAnimaliaDipteraDolichopodidae

Loew

894DC197-FFD7-561E-8884-3EB7DA076B21

Plagioneurus univittatus Loew, 1857: 43.

###### Material examined.

**Dominica**: 1 ♂, Springfield Estate, yellow pans, 1–3 June 2011, M.A. & L.L. Ivie; 1 ♀, same as previous, Malaise trap, 29 May–11 June 2011. **Montserrat**: 1 ♂, Woodlands, Riverside House, 8–10 January 2002, yellow pan traps, K. Marske & K. Puliafico; 1 ♀, Sweetwater Ghaut, 1 August 2005, yellow pan traps, V.G. Martinson (MTEC, USNM).

###### Distribution.

Widely distributed in the New World and reported from the eastern Nearctic, Central America, South America, and the West Indies (Robinson 1964; Pollet et al. 2004).

### Subfamily Sympycninae

#### Genus *Sympycnus* Loew

##### Key to the species of *Sympycnus* in Montserrat

**Table:** 

1	Thorax with 5 pairs of dorsocentral setae; body size ca. 1.5 mm	***S.pentachaetus* Robinson**
–	Thorax with 6 pairs of dorsocentral setae; body size ca. 2.2 mm	***S.montserratensis* sp. nov .**

##### Sympycnus montserratensis

Taxon classificationAnimaliaDipteraDolichopodidae

sp. nov .

A7C3724B-2E0F-51CE-B37B-6EBE03CE0599

http://zoobank.org/9A4E8EE3-CA47-4C80-A185-55AB26BD2A9C

Fig. 32

###### Type material.

***Holotype***, ♂ labelled: “WEST INDIES: MONTSERRAT/ Katy Hill (top), 730 m/ 16°45.731'N, 62°11.646'W/ 28 JUNE 2017, J.B. Runyon”; “HOLOTYPE/ ♂ *Sympycnus*/ *montserratensis*/ Runyon [red label]” (USNM, type number USNMENT01350617). ***Paratypes*: Montserrat**: 1 ♂, 6 ♀, same data as holotype (MTEC, USNM).

###### Description.

**Figure 32.**
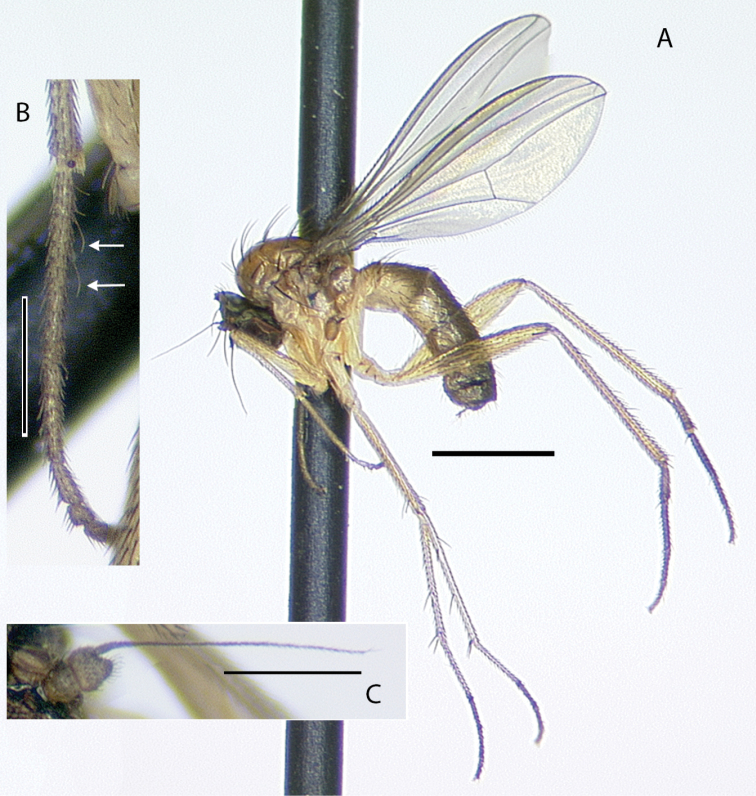
*Sympycnusmontserratensis* sp. nov. male **A** habitus of holotype, left lateral **B** tarsus I, posterior view, arrows indicate slender distally-curved ventral setae **C** male antenna, medial view. Scale bars: 0.5 mm (**A**), 0.25 mm (**B, C**).

**Male** (Fig. 32A). Body length 2.2 mm, wing length 2.2 × width 0.8 mm. ***Head***: Eyes essentially contiguous below, face very narrow throughout (most of face ≤ width of one ommatidium), very slightly widened near mouth and narrowly triangular immediately below antennae, dark brown with thick brown-gray pruinosity. Frons and occiput dark brown with dense brown-gray pruinosity. Palpus very small, narrowly oval, dark yellow with two small black setae near apex. Proboscis brown, with fine brown hairs along margin. Antenna (Fig. 32C) with scape and very base of first flagellomere yellow, remainder brown; first flagellomere broadly triangular with rounded apex, as long as high; arista-like stylus arising from middle of dorsal edge. Lower postocular setae longer, whitish, upper postocular setae black. ***Thorax***: Scutum and scutellum yellow-brown with black setae; acrostichal setae absent; six pairs of dorsocentral setae, 5^th^ and 6^th^ pairs smaller, 5^th^ pair slightly out of line; scutellum with one pair of large marginal setae and one pair of minute lateral hairs. Pleuron and metepimeron yellow, anepimeron with small black triangular area below wing insertion; proepisternum with two small, fine pale setae. ***Legs***: Yellow, except coxa II with small brown streak at insertion of ad seta and small black posterobasal spot; tarsi becoming faintly brown distally. Anterior surface of coxa I with short yellow to brown setae becoming larger distally and ca. five black setae across apex. Femur I with small subapical pv seta; femur II with subapical anterior and pv seta; femur III with subapical anterior seta. Tibia I without distinctive setae; tibia II with ad seta near 1/3 and 2/3, pd near 1/5, pv near 2/3, and 4 apical setae; tibia III with ad seta near 1/5, and small rather indistinct pd seta just before 1/5, 2/5, just beyond 1/2, near 2/3, and 4 apical setae. Tarsus I (Fig. 32B) with tarsomere I(1) bearing two longer slender distally-curved ventral setae in basal half and a slightly shorter such seta at apex, and series of ca. 4–6 very short slender more erect ventral setae scattered along length (two near base most distinct). Ratios of tibia:tarsomeres: leg I: 32-18-9-4-2-3; leg II: 45-22-10-8-5-4; leg III: 58-12-16-10-6-5. ***Wing***: Surface brownish tinged, slightly darker brown anteriorly, with dark brown veins; narrowly elliptical without anal lobe. R_2+3_ essentially straight. R_4+5_ distinctly arching backward and slightly convergent with M_1_ near apex. M_1_ arching backwards slightly near apex, ending in wing tip. Crossvein dm-cu two-thirds as long as last part of CuA_1_. Calypter brown with fan of rather long yellow to light brown setae. Halter knob brownish yellow and stem yellow. ***Abdomen***: Cylindrical, slender, longer than thorax, with black setae. Tergites dark brown dorsally, except tergite I (and sometimes part of tergite II) yellow-brown dorsally; tergites I–V broadly yellow laterally; tergite 6 wholly dark brown. Sternites yellow. Abdomen without metallic reflections. Hypopygium very small, dark brown, mostly concealed in tip of preabdomen, not dissected. Cerci brown, narrowly triangular (twice as long as wide), covered with short brown hairs with longer nearly white hairs at apex.

**Female.** Body length 2.2–2.3 mm, wing length 2.2–2.3 × width 0.8–0.9 mm. Similar to male, but face broader (one-third width of frons), parallel-sided, upper face with gray pruinosity, clypeus with gray-brown pruinosity; palpus larger, dark yellow to nearly brown with white tip; dorsum of thorax more yellow with scutellum brown; tarsus I unmodified, but tarsus I(1) usually with one very short slender curved ventral seta evident at base; abdomen generally more yellow, curved downward when dry.

###### Etymology.

This species is named for the island of Montserrat.

###### Distribution.

Montserrat.

###### Remarks.

*Sympycnusmontserratensis* is related to *S.dominicensis* Robinson (Dominica) but differs most notably in color of the thorax (yellow-brown in *S.montserratensis*, brown in *S.dominicensis*) and form of front tarsus in males (e.g., position of slender curved ventral setae and relative length of tarsomeres; cf. Robinson 1975: fig. 194). Specimens were taken by sweeping shaded moist leaf-covered ground in elfin woodland on the top Katy Hill (Fig. 2C).

The hypopygium was not dissected to preserve intact the two male specimens and because male terminalia in Sympycninae frequently offer no reliable characters to separate species within a genus (e.g., Hurley and Runyon 2009; Evenhuis 2012; Runyon 2012).

##### Sympycnus pentachaetus

Taxon classificationAnimaliaDipteraDolichopodidae

Robinson

970F9EFC-C238-5B6C-80E2-602C3A6BB21F

Sympycnus pentachaetus Robinson, 1975: 106.

###### Material examined.

**Dominica**: 1 ♂, 3 ♀, St. David Parish, ca. 1 km NE Ponte Casse, Waitukubuli National Trail, 15.381490N, 61.340138W, Malaise trap, 31 May–5 June 2011. **Montserrat**: 1 ♀, Jack Boy Hill (top), 480 m, 16°45.797'N, 62°10.886'W, 25 June 2017, J.B. Runyon; 3 ♂, 3 ♀, Big River, 450 m, 16°45.690'N, 62°11.174'W, 28 June 2017, J.B. Runyon; 1 ♂, Bottomless Ghaut, 400 m, 16°45.994'N, 62°11.497'W, 28 June 2017, J.B. Runyon (MTEC, USNM).

###### Distribution.

Dominica, Montserrat.

###### Remarks.

*Sympycnuspentachaetus* adults were collected by sweeping shaded moist ground and streamside rocks in higher elevation mesic forests but at lower elevations than *S.montserratensis*.

### Subfamily Dolichopodinae

#### Genus *Paraclius* Loew

##### Key to the species of *Paraclius* in Montserrat

**Table:** 

1	Pleuron and much of abdomen yellow	***P.megalocerus* Robinson**
–	Most of pleuron and all of abdomen metallic green to blue	***P.* unidentified species (female)**

##### Paraclius megalocerus

Taxon classificationAnimaliaDipteraDolichopodidae

Robinson

CA25C82E-B3D0-5B3E-AA12-2332747E4381

Paraclius megalocerus Robinson, 1975: 111.

###### Material examined.

**Dominica: *Holotype*** ♂, Clarke Hall, 28 February 1964, H. Robinson (USNM). **Montserrat**: 1 ♂, Woodlands, Riverside House, 8–10 January 2002, yellow pan traps, K. Marske & K. Puliafico (MTEC).

###### Distribution.

Dominica, Montserrat.

##### Paraclius

Taxon classificationAnimaliaDipteraDolichopodidae

unidentified species

569B8508-855C-518D-B19F-B7813498C6A8

###### Material examined.

**Montserrat**: 1 ♀, Woodlands, Riverside House, 8–10 January 2002, yellow pan traps, K. Marske & K. Puliafico (MTEC).

###### Remarks.

The female specimen differs from those of *P.megalocerus* in having the pleuron and abdominal tergites metallic green or blue. This species seems most similar to males of *P.pavo* (Aldrich) from St. Vincent (the female is unknown) but cannot be confidently assigned to species.

#### Genus *Tachytrechus* Haliday

##### Tachytrechus perornatus

Taxon classificationAnimaliaDipteraDolichopodidae

Robinson

EADBCE2B-79B3-5BED-B5A2-69DD6B972ABE

Tachytrechus perornatus Robinson, 1975: 122.

###### Material examined.

**Dominica: *Holotype*** ♂, La Ronde River, 15 February 1964, H. Robinson (USNM). **Montserrat**: 2 ♂, 3 ♀, Hope Ghaut, 280 m, 16°45.101'N, 62°12.760'W, 20 June 2017, J.B. Runyon; 1 ♀, same as previous, 300 m, 16°45.108'N, 62°12.695'W (MTEC, USNM).

###### Distribution.

Dominica, Montserrat.

###### Remarks.

Adults were collected from rocks in a small stream with flowing water.

### Subfamily Hydrophorinae

#### Genus *Cymatopus* Kertész

##### Cymatopus bredini

Taxon classificationAnimaliaDipteraDolichopodidae

Robinson

B2782072-14BE-5C84-87EE-0DED84DD672C

Cymatopus bredini Robinson, 1975: 125.

###### Material examined.

**Dominica: *Holotype*** ♂, Calibishie seashore, 27 February 1965, W.W. Wirth (USNM). **Montserrat**: 11 ♂, 5 ♀, Rendezvous Bay Beach, rocks in intertidal zone, 16°48.489'N, 62°12.296'W, 23 June 2017, J.B. Runyon. **St. Kitts**: 1 ♂, North Friars Bay, 17°16.59'N, 62°40.33'W, 24 May 2017, J.B. Runyon (MTEC, USNM).

###### Distribution.

Lesser Antilles (Antigua, Dominica, Montserrat, St. Kitts).

###### Remarks.

Montserrat specimens were obtained by sweeping partially shaded nearly vertical walls in the splash zone of large rock outcrops at the south end of Rendezvous Bay Beach.

#### Genus *Thinophilus* Wahlberg

##### Thinophilus ochrifacies

Taxon classificationAnimaliaDipteraDolichopodidae

Van Duzee

4187C81E-EF3F-5B0B-A412-DA9ABA8D7430

Thinophilus ochrifacies Van Duzee, 1924a: 101.

###### Material examined.

**Anguilla**: 2 ♀, Sombrero, 18°35.17'N, 63°25.63'W, small freshwater pool, 12–13 November 1999, M.A. Ivie & J.B. Runyon. **Montserrat**: 4 ♂, 1 ♀, Fox’s Bay Beach, 16°43.59'N, 62°14.17'W, 23 June 2017, J.B. Runyon. **St. Kitts**: 1 ♂, South Frigate Bay, 17°16.869'N, 62°41.201'W, 24 May 2017, J.B. Runyon. **St. Lucia**: 7 ♂, 6 ♀, Savannes, Mangrove Reserve, 0–5 m, 13°45.97'N, 60°54.88'W, 3 May 2009, J.B. Runyon; 4 ♂, 3 ♀, Fond Bay at beach, 0–5 m, 13°49.89'N, 60°53.65'W, 8 May 2009, J.B. Runyon (MTEC, USNM).

###### Distribution.

Nova Scotia, Canada south to Mexico and the West Indies (Robinson 1975).

###### Remarks.

This species is restricted to coastal areas. Adults were found in Montserrat on open mud at edges of a drying freshwater pool at the back of Fox’s Bay Beach.

## Discussion

### Summary of Montserrat fauna

The list of Montserrat Dolichopodidae includes 63 species in 27 genera (Table 2). Two-thirds of Montserrat’s species (41 spp. or 65%) are endemic to the Lesser Antilles, 10% (6 spp.) are restricted to the wider West Indies, and ca. 25% (15 spp.) are widespread natives that also occur on the mainland. Six species are known only from Montserrat and could be single island endemics (*Amblypsilopusmarskeae*, *Chrysotusmontserratensis*, *C.interfrons*, *Medeteraiviei*, *M.montserratensis*, and *Sympycnusmontserratensis*). There is no evidence that any dolichopodid species was introduced by human activities, and all appear to naturally occur on Montserrat.

How many dolichopodid species occur on Montserrat? The species list provided here is undoubtedly incomplete. Based on the Chao 1 estimator (Chao 1984), which takes into account the number of species represented by precisely one (eight species), and the number represented by precisely two (four species) individuals, it is estimated there are an additional eight species to be discovered, and when fully known Montserrat’s fauna will contain approximately 71 species of Dolichopodidae. The rarefaction curve showing accumulation of unique species levels off but does not reach an asymptote (Fig. 3), suggesting more species will be discovered if sampling effort is further increased. Extrapolation of this curve estimates that doubling the original effort (i.e., collecting another 1500 specimens) would yield ca. six additional species. However, focused collecting in habitats inadequately sampled to date (e.g., high elevations) or likely to support the most diverse dolichopodid communities (e.g., deep ghauts, permanent streams) will more efficiently allow collection of undiscovered species. An important caveat is that less than half the island was sampled due to the volcano exclusion zone and surveying intact habitats on the southern half of Montserrat, e.g., the sizeable remaining higher-elevation forests in the Roaches area, could add even more species.

### Comparison of Dominica and Montserrat faunas

Dominica and Montserrat are the only two islands in the Lesser Antilles with reasonably well-sampled and described dolichopodid faunas. The 63 species on Montserrat (ca. 100 km^2^) is greater than half of the 119 species known from Dominica (ca. 750 km^2^). For species-area relationship (Preston 1962) this gives a preliminary value of 15.1 for c and 0.311 for z for Dolichopodidae in the volcanic islands of the Lesser Antilles. Clearly, a better understanding of the dolichopodid faunas of other islands in the Lesser Antilles is needed to accurately assess the species-area relationship of the region. Using Darlington’s rule of thumb (MacArthur and Wilson 1967) that a ten-fold increase in area results in a doubling of species richness, Montserrat has more species than predicted relative to Dominica, and this is further supported by the fact that less than half of Montserrat’s area was sampled (due to the exclusion zone). However, the respective faunas are not completely known, and it seems likely that Dominica has more species remaining to be discovered than Montserrat.

The vast majority of species on Montserrat also occur on Dominica (ca. 87%), indicating that Dolichopodidae, in general, are effective at dispersing. Montserrat has 27 of the 33 genera occurring on Dominica (missing *Discopygiella* Robinson, *Dominicomyia* Robinson, *Haromyia* Runyon, *Micromedetera* Robinson, *Pelastoneurus* Loew, and *Pseudosympycnus* Robinson). Twenty-two species are currently known to occur only on Dominica and Montserrat. These latter species also likely occur in Guadeloupe (which has no published records of dolichopodids) and possibly other neighboring islands.

Among the dolichopodid species or genera that are present on Dominica but missing from Montserrat, some can be explained by habitat diversity, especially wet habitats. For example, the general lack of standing fresh water (e.g., lakes or swamps) on Montserrat could explain the absence of *Pelastoneurus* (three species on Dominica) and some species of *Thrypticus* (possibly due to absence of their aquatic host plants). Moreover, several species on Dominica appear restricted to larger permanent rivers (e.g., *Micromedetera*), a habitat absent on Montserrat. Elevation also plays a role since Dominica’s maximum elevation (ca. 1,450 m) and amount of high elevation habitat is greater than Montserrat’s (maximum elevation ca. 1,050 m) and some Dominica species not on Montserrat seem restricted to these higher habitats (e.g., *Pseudosympycnus*). Montserrat’s highest intact habitat is Katy Hill (741 m) with the highest elevations on the island, the peaks of the Soufrière Hills, destroyed by volcanic activity – if higher elevation species occurred there, they are now almost certainly gone. The volcanic nature and small size of Montserrat could also result in periodic extinction events, perhaps for the whole island, requiring recolonization and leading to lower island endemicity. A component underrepresented on Montserrat is the so-called ‘micro-dolichopodids’, species in several genera with body size ca. 1.0 mm (Runyon and Robinson 2010). These species can be difficult to collect due to their small size, elusive habits, and microhabitat specialization (Robinson 1969, 1975, Runyon and Pollet 2018). For example, it seems very likely that additional species of the micro-dolichopidid genus *Enlinia* occur on Montserrat as two or more species occur on West Indian islands of similar or smaller size (Runyon, unpublished data). Targeted net collecting focusing on areas with permanent or semi-permanent water will likely lead to discovery of more micro-dolichopodid species on Montserrat.

### Comparison of collecting methods

Five collecting methods were used in this survey: Malaise traps, pan traps, ultraviolet light traps, canopy fogging, and targeted net collecting. All methods collected dolichopodid specimens, but most productive were net collecting (40 spp.), pan traps (33 spp.), and Malaise traps (29 spp.). Canopy fogging and ultraviolet light traps each collected ten species. Twenty-eight species were caught by only one collecting method, 20 species by two methods, nine by three methods, three by four methods, and three species were caught by all five collecting methods. Of the species unique to one method, net collecting (14 unique spp.), Malaise traps (8 unique spp.), and pan traps (6 unique spp.) caught the most. Canopy fogging and ultraviolet light traps caught no unique species, suggesting that inventorying dolichopodids with net collecting, pan and Malaise traps is sufficient. However, trap types were not deployed equally across time and habitats which likely influenced their relative effectiveness.

The relatively large number of species unique to net collecting can be largely explained by the habitat preferences of these species and the difficulty in using passive traps in such habitats. For example, four coastal/littoral species (*Chimerothalassiusrunyoni*, *Chrysotusangustifrons*, *Cymatopusbredini*, and *Thinophilusochrifacies*) were only obtained by net collecting in areas where trapping was not possible or attempted. Moreover, passive trapping caught just three specimens (all female) of the two beach-inhabiting species of *Asyndetus*, whereas dozens of specimens (including males) of both species were readily obtained with a net. Other examples of species caught only by net include those restricted to rocks in streams (*Enliniapatellitarsis*, *Peloropeodesfrater*, *Sympycnuspentachaetus*, and *Tachytrechusperornatus*) or the highest elevations (e.g., *Symbolialinearis* and *Sympycnusmontserratensis*), habitats in which it is difficult to place and maintain traps. Another contributing factor is the 2001–2005 survey focused primarily on Coleoptera and traps were not specifically placed in areas to target dolichopodids. This underscores the importance of targeted net collecting or careful trap placement during inventories, e.g., to avoid missing habitat-specific species of Diptera.

### Threats to the Montserrat fauna

Although the Soufrière Hills volcano has been quiet in recent years, additional volcanic activity remains the primary threat to Montserrat dolichopodids. Since volcanic activity began in 1995, approximately 60% of the forest cover on Montserrat has been lost (Young 2008). Additional volcanic activity could destroy remaining intact habitats and ash fall events can have significant negative effects on arthropods (Marske et al. 2007). Ash fall can severely negatively affect water quality and associated riparian areas – habitats that many species of dolichopodids depend on – and smaller waterways, like those on Montserrat, are most affected (Miserendino et al. 2012).

The Centre Hills contains the largest remaining tract of forest in Montserrat (Fig. 1), and most species of Dolichopodidae were found there. Most of the mid to upper elevations of the Centre Hills were protected in 2000 under the Protected Forest Order and Forest Reserve Order of the Forestry, Wildlife, National Parks and Protected Areas Act (Young 2008). However, most of the lower elevations of the Centre Hills and dry/littoral forests remain unprotected. Three of the six species of dolichopodids endemic to Montserrat appear restricted to these lower elevation habitats (*Amblypsilopusmarskeae*, *Chrysotusinterfrons*, and *Medeteraiviei*), and protecting areas representative of these habitats could ensure conservation of these unique species. Protection of these drier habitats was among the highest priority conservation recommendations resulting from the Centre Hills Biodiversity Assessment (Young 2008).

**Figure 33.**
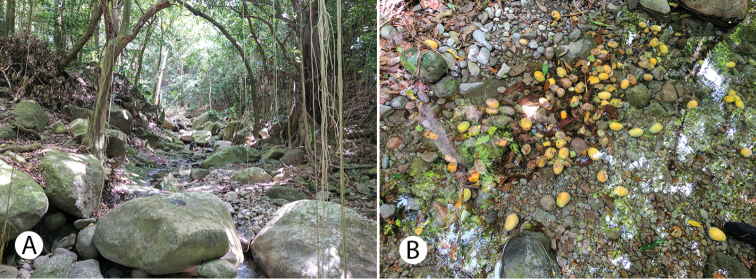
The presence of large numbers of mango trees seemed to negatively affect the abundance and diversity of dolichopodids along some Montserrat streams **A** Fogarty Ghaut seems an ideal place for Dolichopodidae, but few were found **B** large numbers of mango fruit and leaves filled the stream and stream sides in Fogarty Ghaut, suggesting that chemicals from this material could be killing aquatic or semi-aquatic insects. Photographs by Justin Runyon.

Of final note is nonnative mango (*Mangiferaindica* L.) and its possible negative effects on dolichopodids and other aquatic or semi-aquatic insects. Mango is native to South Asia and cultivated widely in the tropics, including Montserrat. The skin of the fruit, leaves, and bark contain chemical compounds that can cause contact dermatitis in humans (Oka et al. 2004) and be toxic to insects. For example, aqueous extracts of *M.indica* are known to kill Diptera larvae (e.g., mosquitoes; Rahuman et al. 2009; Zuharah et al. 2014) and have been used to control aphid, beetle, and moth pests in cowpea crops in Africa (Kossou et al. 2001). Mango trees are very abundant along some lower elevation waterways and fruits and leaves fall in large numbers into these streams and streamside habitats (Fig. 33B). Dolichopodids and other aquatic insects were largely absent at several sites on Montserrat invaded by large numbers of mango trees (e.g., very few individuals of just two or three species found at Fairy Walk River and Fogarty Ghaut) that are seemingly ideal habitats that should support a diverse and abundant dolichopodid community (Fig. 33A). Species that were abundant along streams with lots of mangos were those that are not strictly aquatic or semi-aquatic (e.g., *Medetera* spp., whose larvae live under tree bark). This phenomenon was also observed on St. Kitts (Runyon, personal observation). More research is needed to determine if mangoes are responsible. If so, limiting spread or reducing the numbers of large mango trees might be an effective way to improve habitat for insects (upon which other organisms depend, including the endemic and critically endangered Montserrat oriole). However, control of mangos would need to be balanced with the important habitat large trees can provide for the endemic orchid *Epidendrummontserratense* Nir (Young 2008), agriculture, and the livelihoods of those that collect fruits to eat or sale.

## Supplementary Material

XML Treatment for Chimerothalassius runyoni

XML Treatment for Amblypsilopus luteus

XML Treatment for Amblypsilopus marskeae

XML Treatment for Condylostylus albiciliatus

XML Treatment for Condylostylus longicornis

XML Treatment for Condylostylus nigripilosus

XML Treatment for Condylostylus quadricolor

XML Treatment for Coeloglutus concavus

XML Treatment for Dactylomyia decora

XML Treatment for Neurigona fuscicosta

XML Treatment for Viridigona thoracica

XML Treatment for Cryptopygiella musaphila

XML Treatment for Medetera crassicauda

XML Treatment for Medetera dominicensis

XML Treatment for Medetera iviei

XML Treatment for Medetera montserratensis

XML Treatment for Medetera pseudonigripes

XML Treatment for Systenus ladonnae

XML Treatment for Thrypticus abdominalis

XML Treatment for Thrypticus aequalis

XML Treatment for Thrypticus mediofuscus

XML Treatment for Thrypticus parvulus

XML Treatment for Thrypticus violaceus

XML Treatment for Xanthina rubromarginata

XML Treatment for Enlinia patellitarsis

XML Treatment for Harmstonia simplex

XML Treatment for Micromorphus albipes

XML Treatment for Peloropeodes frater

XML Treatment for Achradocera apicalis

XML Treatment for Asyndetus interruptus

XML Treatment for Asyndetus fratellus

XML Treatment for Chrysotus acutus

XML Treatment for Chrysotus albihirtipes

XML Treatment for Chrysotus angustifrons

XML Treatment for Chrysotus antillensis

XML Treatment for Chrysotus brevicornis

XML Treatment for Chrysotus callichromoides

XML Treatment for Chrysotus callichromus

XML Treatment for Chrysotus hirsutus

XML Treatment for Chrysotus interfrons

XML Treatment for Chrysotus integer

XML Treatment for Chrysotus lamellicaudatus

XML Treatment for Chrysotus mediocaudatus

XML Treatment for Chrysotus microtatus

XML Treatment for Chrysotus montserratensis

XML Treatment for Chrysotus orichalceus

XML Treatment for Chrysotus parvulus

XML Treatment for Chrysotus milvadu

XML Treatment for Chrysotus proximus

XML Treatment for Chrysotus pseudoniger

XML Treatment for Chrysotus spectabilis

XML Treatment for Chrysotus spinipes

XML Treatment for Chrysotus xiphostoma

XML Treatment for Diaphorus contiguus

XML Treatment for Diaphorus robinsoni

XML Treatment for Symbolia linearis

XML Treatment for Plagioneurus univittatus

XML Treatment for Sympycnus montserratensis

XML Treatment for Sympycnus pentachaetus

XML Treatment for Paraclius megalocerus

XML Treatment for Paraclius

XML Treatment for Tachytrechus perornatus

XML Treatment for Cymatopus bredini

XML Treatment for Thinophilus ochrifacies

## Footnotes

Modified from Robinson 1975 and Bickel 2009.
